# Koumine Suppresses IL-1β Secretion and Attenuates Inflammation Associated With Blocking ROS/NF-κB/NLRP3 Axis in Macrophages

**DOI:** 10.3389/fphar.2020.622074

**Published:** 2021-01-18

**Authors:** Yufei Luo, Bojun Xiong, Haiping Liu, Zehong Chen, Huihui Huang, Changxi Yu, Jian Yang

**Affiliations:** ^1^ Department of Pharmacology, School of Pharmacy, Fujian Medical University, Fuzhou, China; ^2^ Experimental Teaching Center, School of Basic Medical Sciences, Fujian Medical University, Fuzhou, China; ^3^ Fujian Key Laboratory of Drug Target Discovery and Structural and Functional Research, School of Pharmacy, Fujian Medical University, Fuzhou, China

**Keywords:** koumine, NLRP3 inflammasome, NF-κ B, reactive oxygen species, macrophages

## Abstract

Koumine (KM), one of the primary constituents of Gelsemium elegans, has been used for the treatment of inflammatory diseases such as rheumatoid arthritis, but whether KM impacts the activation of the NOD-like receptor protein 3 (NLRP3) inflammasome remains unknown. This study aimed to explore the inhibitory effect of KM on NLRP3 inflammasome activation and the underlying mechanisms both *in vitro* using macrophages stimulated with LPS plus ATP, nigericin or monosodium urate (MSU) crystals and *in vivo* using an MSU-induced peritonitis model. We found that KM dose-dependently inhibited IL-1β secretion in macrophages after NLRP3 inflammasome activators stimulation. Furthermore, KM treatment efficiently attenuated the infiltration of neutrophils and suppressed IL-1β production in mice with MSU-induced peritonitis. These results indicated that KM inhibited NLRP3 inflammasome activation, and consistent with this finding, KM effectively inhibited caspase-1 activation, mature IL-1β secretion, NLRP3 formation and pro-IL-1β expression in LPS-primed macrophages treated with ATP, nigericin or MSU. The mechanistic study showed that, KM exerted a potent inhibitory effect on the NLRP3 priming step, which decreased the phosphorylation of IκBα and p65, the nuclear localization of p65, and the secretion of TNF-α and IL-6. Moreover, the assembly of NLRP3 was also interrupted by KM. KM blocked apoptosis-associated speck-like protein containing a CARD (ASC) speck formation and its oligomerization and hampered the NLRP3-ASC interaction. This suppression was attributed to the ability of KM to inhibit the production of reactive oxygen species (ROS). In support of this finding, the inhibitory effect of KM on ROS production was completely counteracted by H_2_O_2_, an ROS promoter. Our results provide the first indication that KM exerts an inhibitory effect on NLRP3 inflammasome activation associated with blocking the ROS/NF-κB/NLRP3 signal axis. KM might have potential clinical application in the treatment of NLRP3 inflammasome-related diseases.

## Introduction

Inflammation plays a key role in triggering host defense responses by clearing pathogens. However, uncontrolled inflammation may cause tissue damage and lead to serious disorders by massive inflammatory cytokines production, especially the IL-1β. The activation of NOD-like receptor protein 3 (NLRP3) inflammasome is the major mechanism in IL-1β maturation and secretion (Pedraza-Alva et al., 2015). Inflammasomes, which are multiprotein complexes, are key mediators of inflammatory responses and serve as signaling platforms to orchestrate host defense in response to pathogen-associated molecular patterns (PAMPs) and danger-associated molecular patterns (DAMPs) released by infectious agents or noninfectious damage (Martinon et al., 2002). The NLRP3 inflammasome has been widely studied and contains the sensor protein NLRP3, an adaptor protein named apoptosis-associated speck-like protein containing a caspase-containing domain (ASC), and effector protein the proforma of caspase-1 (pro-caspase-1) (Broz and Dixit, 2016; Mangan et al., 2018). The classical activation of the NLRP3 inflammasome requires two signals (Guo et al., 2015): for signal 1 (also called priming), toll-like receptors (TLR) agonists such as LPS activate the NF-κB pathway and induce the expression of NLRP3 and pro-IL-1β; and for signal 2, PAMPs or DAMPs, such as extracellular adenosine triphosphates (ATP), nigericin and monosodium urate (MSU) crystals, disrupt cellular physiology, which initiates the assembly of the inflammasome protein complex. The assembled NLRP3 inflammasome leads to the activation of caspase-1, which autocleaves pro-caspase-1 into p20 and p10 subunits and then cleaves pro-IL-1β to generate mature IL-1β (Huang et al., 2018). Excessive and persistent activation of the NLRP3 inflammasome leads to loss of the control of IL-1β processing and contributes to several immune diseases, such as rheumatoid arthritis, systemic lupus erythematosus, gout and colitis (Martinon et al., 2006; Yang et al., 2015; Guo et al., 2018; Mai et al., 2019). Thus, disturbing the molecular compounds of the NLRP3 inflammasome and inhibiting NLRP3 inflammasome activity constitute a promising approach for the treatment of inflammatory disorders.

The exact molecular events leading to the activation of NLRP3 remain elusive. Various molecular mechanisms have been proposed to explain the activation of the NLRP3 inflammasome, and these include reactive oxygen species (ROS) generation, pore formation, intracellular ionic fluxes and lysosomal destabilization (Petrilli et al., 2007; Heid et al., 2013; Elliott and Sutterwala, 2015; Gurung et al., 2015). Among these mechanisms, ROS, which are unstable and highly reactive molecules produced by the reduction of oxygen mainly during mitochondrial oxidative phosphorylation (Harijith et al., 2014), are considered a common trigger of NLRP3 inflammasome activation (Harijith et al., 2014). ROS provide a priming signal for NLRP3 inflammasome activation by activating the NF-κB signaling pathway and increasing NLRP3 and proIL-1β expression (Elliott and Sutterwala, 2015; Long et al., 2020). Moreover, ROS provide an activation signal for the assembly and activation of the NLRP3 inflammasome. Treatment with NLRP3 agonists triggers ROS production and promotes the dissociation of oxidoreductase thioredoxin/thioredoxin-interacting protein (TXNIP), which leads to the binding of TXNIP to NLRP3 and thus NLRP3 activation (Zhou et al., 2010; Zhou et al., 2011; Abais et al., 2015).

Koumine (KM) is a type of alkaloid derived from Gelsemium elegans, which is used as a traditional medicinal plant in China and other Asian countries (Jin et al., 2014). Recent studies have demonstrated that KM has diverse pharmacological activities, including anti-psoriatic, anti-inflammatory, anti-tumor, analgesic, antioxidant and anxiolytic activities (Xu et al., 2012; Chen et al., 2017; Jin et al., 2018b; Jin et al., 2019; Yuan et al., 2019a; Yuan et al., 2019b; Wu et al., 2020). Numerous studies have shown that KM exerts anti-inflammatory effects by inhibiting the production of reactive oxygen species (ROS), regulating the Nrf2/NF-κB signaling pathway and decreasing the production of IL-1β, IL-6, TNF-α and nitric oxide (Yuan et al., 2016a; Wu et al., 2020). Our studies also found that KM exerts an anti-inflammatory effect in inflammatory pain and rheumatoid arthritis by inhibiting IL-1β and TNF-α production (Xu et al., 2012; Yang et al., 2016; Jin et al., 2019). Thus, because KM can inhibit ROS production and NF-κB activation, which are both upstream of NLRP3 inflammasome activation, we hypothesized that KM would exert anti-inflammatory effects by inhibiting ROS-induced activation of NF-κB and the NLRP3 inflammasome. The aim of this study was to investigate the potential role of KM in NLRP3 inflammasome activation and explore the molecular mechanism related to the ROS/NF-κB/NLRP3 pathway.

## Materials and Methods

### Animals

C57BL/6 male mice (20 ± 2 g) were purchased from the Shanghai Laboratory Animal Center (Shanghai, China; certificate number SCXK2017-0005). After a one-week acclimatization period, the mice were maintained at 24 ± 2°C under a 12-h light/12-h dark cycle and given free access to food and water. The animal experiments were performed at the Experimental Animal Center of Fujian Medical University and were conducted in accordance with the Guide for the Care and Use of Laboratory Animals (NIH Publications No. 8023, revised 1978) and with prior approval from the ethics committee at Fujian Medical University (approval number: 2019-0057).

### Chemicals and Antibodies

**FIGURE 1:**
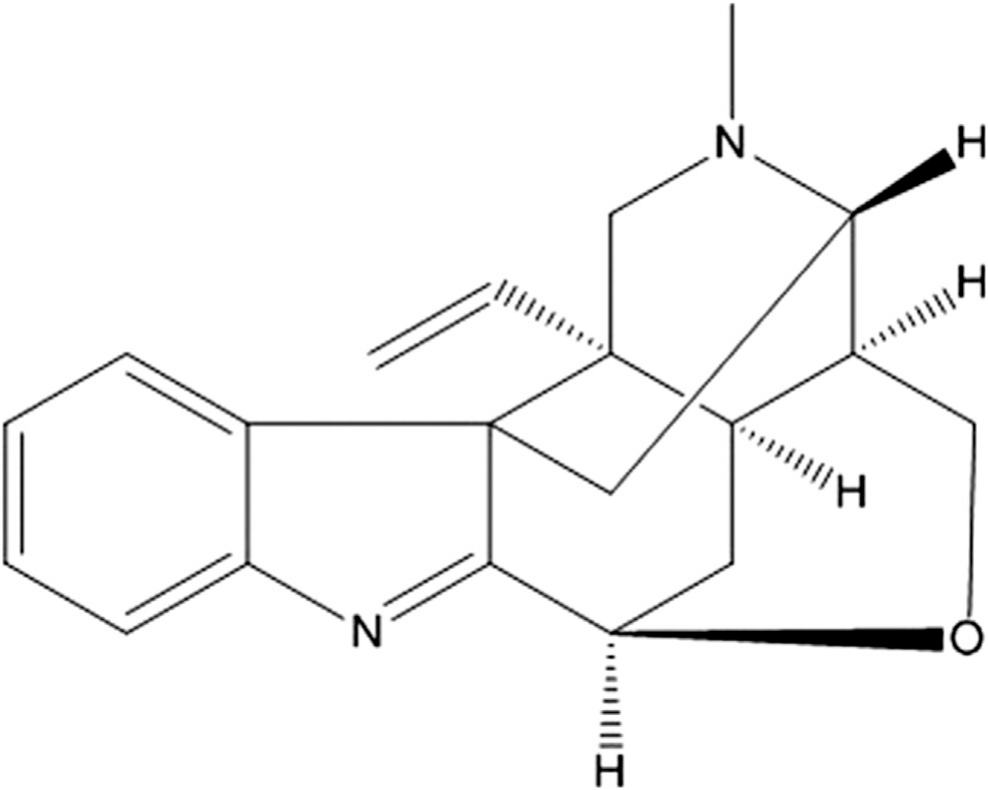
The chemical structures of koumine (KM). The molecular formula is C_20_H_22_N_2_O. The molecular weight is 306.40. The CAS registry number is 1358-76-5.

Koumine (PubChem CID: 91895267, KM, Figure 1, purity >99%; HPLC) was isolated from *G. elegans* Benth. via pH-zone-refining countercurrent chromatography, as described previously (Su et al., 2011). KM was dissolved in PBS to a concentration of 20 mM, and each solution was then serially diluted to specific concentrations. ATP and MSU were purchased from Sigma (St. Louis, MO, United States). Nigericin, MCC950 and phorbol 12-myristate 13-acetate (PMA) were obtained from Meilunbio® Co., Ltd. (Dalian, China). LPS was purchased from InvivoGen (tlrl-smlps, San Diego, CA, United States). Anti-β-actin (1:5,000) was purchased from Thermo Fisher (Waltham, MA, United States), and anti-NLRP3 (1:1,000, AG-20B-0014) was procured from AdipoGen Life Science (San Diego, CA, United States). Anti-caspase-1 (1:1,000, ab179515) was purchased from Abcam (Cambridge, MA, United States), and anti-mouse ASC (1:1,000, 67824), anti-human ASC (1:1,000, 13833), anti-IL-1β (1:1,000, 12242), anti-p-IκBα (1:1,000, 2859), anti-IκBα (1:1,000, 4814), anti-p-p65 (1:1,000, 3033), anti-p65 (1:1,000, 6956), and anti-rabbit IgG isotype control (8726) were obtained from Cell Signaling Technology (Waltham, MA, United States). Protein A/G plus agarose (SC-2003) was purchased from Santa Cruz Biotechnology (Santa Cruz, CA, United States).

### Cell Culture and Stimulation

The L929 cell line was obtained from Cellcook (Guangzhou, China), and the THP-1 cell line was obtained from Stem Cell Bank, Chinese Academy of Sciences (Shanghai, China). L929 and THP-1 cells were cultured in complete Dulbecco’s modified Eagle’s medium (DMEM) and RPMI 1640 medium (HyClone, South Logan, UT, United States), respectively. Each medium was supplemented with 10% fetal bovine serum (FBS, Gibco, CA, United States) and 1% penicillin-streptomycin (P/S, GA3502, GENVIEW, Tallahassee, FL, United States). The cells were maintained in a humidified incubator at 37°C with 5% CO_2_.

Bone marrow-derived macrophages (BMDMs) were obtained through the differentiation of bone marrow progenitors from the tibia and femur in DMEM containing 30% L929-conditioned medium (BM-medium), 10% FBS, and 1% P/S for 7 days. The culture medium was replaced every other day. The BMDM purity was detected by flow cytometry using CD11b and F4/80 antibodies and was routinely >95%.

To activate the NLRP3 inflammasome, BMDMs or THP-1 cells were plated in 24- or 6-well plates and cultured overnight. The following day, the medium was changed to Opti-MEM. The cells were primed with 300 ng/ml LPS for 3 h and then treated with 5 mM ATP for 1 h, 10 µM nigericin for 1 h, or 150 μg/ml MSU for 6 h. THP-1 cells were differentiated by 100 nM PMA treatment for 3 h and then incubated overnight with fresh medium before treatment with NLRP3 inflammasome activators. The supernatants and cell lysates were collected for ELISA and western blotting assays.

### Flow Cytometry Analysis

Flow cytometry analysis was performed as previously described (Li et al., 2019). After 7 days of differentiation in culture conditions, BMDMs were collected, washed with PBS, resuspended in 100 μl of binding buffer (BD Pharmingen, San Diego, CA, United States), labeled with 5 μl of CD11b-APC (561690, BD Biosciences, San Jose, CA, United States) and 5 μl of F4/80-PE (565410, BD Biosciences, San Jose, CA, United States) and incubated for 15 min in the dark. Binding buffer (400 μl) was added, and the fluorescence was detected with the CytoFLEX platform (Beckman Coulter, Miami, FL, United States). The cells retrieved from the peritoneal lavage fluid were labeled with CD11b-PE (557397, BD Biosciences, San Jose, CA, United States) and Ly6G-FITC (553126, BD Biosciences, San Jose, CA, United States) and then analyzed by flow cytometry. Data were acquired from 10,000 gated events.

### Enzyme-Linked Immunosorbent Assay (ELISA)

IL-1β (88-7013-88), TNF-α (88-7324-88) and IL-6 (88-7064-88) were detected using ELISA kits (Invitrogen, Carlsbad, CA, United States) according to the manufacturer’s instructions.

### Proliferation Assay

The viability of the treated cells was evaluated with the MTT assay. Briefly, THP-1 and BMDMs were seeded in 96-well plates at 1 × 104 per well. After incubation with KM at the indicated concentration for 24 h, 20 μl of 5 mg/ml MTT (ST316, Beyotime, Shanghai, China) was added to each well, and the plate was incubated for another 4 h. The supernatants were removed, and the formazan crystals were dissolved in 150 μl of DMSO. The absorbance was measured at 570 nm.

The proliferation of the treated cells was evaluated with the CCK-8 assay. The cells were seeded in 96-well plates at 1 × 104 per well. After incubation with KM at the indicated concentration for 24 h, 10 μl of CCK-8 (C0039, Beyotime, Shanghai, China) was added to each well, and the plate was incubated for another 4 h. The absorbance at 450 nm was measured.

### MSU-Induced Peritonitis

KM (0.8, 2.4, 7.2 mg/kg) or MCC950 (10 mg/kg) was administered intraperitoneally 1 h prior to MSU injection (1 mg in 500 μl of PBS). After 6 h, the mice were sacrificed, and peritoneal lavage was conducted with 5 ml of cold PBS. The cells collected from the peritoneal fluid were subsequently analyzed by flow cytometry. The concentrations of IL-1β in the supernatants of the peritoneal lavage fluid samples were determined by ELISA.

### Western Blotting

Total protein from the tissues and cells was collected and lyzed in NP-40 buffer (P0013F, Beyotime, Shanghai, China) with 1 mM PMSF. The nuclear and cytosolic proteins were extracted with a Nuclear and Cytoplasmic Protein Extraction Kit (Beyotime, Shanghai, China) according to the manufacturer’s instructions. The protein concentration was measured using a BCA protein assay kit (P0009, Beyotime, Shanghai, China). Equal amounts (30 μg) of protein were separated by 10–15% SDS-PAGE and then transferred to a PVDF membrane. Subsequently, the PVDF membrane was blocked with 5% (w/v) BSA in Tris-buffered saline containing 0.1% Tween-20 (TBST) buffer for 1 h at room temperature and incubated with the corresponding primary antibodies overnight at 4°C. Subsequently, the membrane was washed three times with TBST and incubated with horseradish peroxidase (HRP)-conjugated secondary antibodies for 1 h at room temperature. Finally, the membrane was washed and visualized with the BeyoECL Star reagent (P0018AM, Beyotime, Shanghai, China). The relative optical densities of the bands were quantified using ImageJ gel analysis software.

### Quantitative Real-Time PCR (RT-qPCR)

Total RNA was extracted from cultured cells with the BIOZOL reagent (BSC51M1, BioFlux) and reverse transcribed into cDNA using a Takara Prime Script RT Reagent Kit (RR047A, Takara). RT-qPCR was performed using a TB Green PCR Kit (RR420A, Takara, Kyoto, Japan) with a QuantStudio 6 Flex Real-Time PCR System. The GAPDH gene was used as an endogenous control. The relative gene expression levels were calculated using the 2-ΔΔCT method. The primer sequences used in this study were as follows: NLRP3, 5′-GAG​CTG​GAC​CTC​AGT​GAC​AAT​GC-3′ (forward) and 5′-ACC​AAT​GCG​AGA​TCC​TGA​CAA​CAC-3′ (reverse); pro-caspase-1, 5′-AGA​GGA​TTT​CTT​AAC​GGA​TGC​A-3′ (forward) and 5′-TCA​CAA​GAC​CAG​GCA​TAT​TCT​T-3′ (reverse); ASC, 5′-ACA​ATG​ACT​GTG​CTT​AGA​GAC​A-3′ (forward) and 5′-CAC​AGC​TCC​AGA​CTC​TTC​TTT​A-3′ (reverse); pro-IL-1β, 5′-TCG​CAG​CAG​CAC​ATC​AAC​AAG​AG-3′ (forward) and 5′-TGA​TCA​TGT​CCT​CAT​CCT​GGA​AGG-3′ (reverse); and GAPDH, 5′-CGC​TGA​GTA​CGT​CGT​GGA​GTC-3′ (forward) and 5′-GCT​GAT​GAT​CTT​GAG​GCT​GTT​GTC-3′ (reverse).

### Immunofluorescence Staining

For the analysis of p65 nuclear translocation, BMDMs or PMA-differentiated THP-1 cells were incubated with KM for 1 h and then treated with LPS (300 ng/ml) for 1 h. The cells were washed with PBS, fixed with 4% paraformaldehyde (PFA) for 30 min, and permeabilized with 0.5% Triton X-100 for 10 min. The cells were then blocked with 5% BSA for 1  h, incubated with anti-p65 antibody (1:100) overnight at 4°C, and stained with CoraLite488-conjugated anti-mouse IgG antibody (1:100, Proteintech, Wuhan, China) for 1 h. The cells were subsequently stained with Hoechst 33342 (Sigma, St. Louis, MO, United States) for 15 min. Images were captured with a Leica DMi8 fluorescence microscope.

For assays of NLRP3 and ASC colocalization, BMDMs or PMA-differentiated THP-1 cells on confocal dishes were fixed, permeabilized and blocked. The cells were stained with anti-NLRP3 and anti-ASC antibodies overnight and then with CoraLite594-conjugated anti-mouse IgG and CoraLite488-conjugated anti-rabbit IgG antibodies (1:100, Proteintech, Wuhan, China) for 1 h. The cells were incubated with Hoechst 33342 and imaged with a Leica DMi8 fluorescence microscope.

### ASC Oligomerization and ASC Speck Assay

BMDMs were pretreated with KM for 1 h and then treated as mentioned above to activate the NLRP3 inflammasome. The supernatants were discarded, and the cells were lyzed with 0.5% Triton X-100 for 30 min. The lysates were centrifuged at 6,000 × g and 4°C for 15 min. The pellets were washed, resuspended in 500 μl of PBS and combined with 2 mM disuccinimidyl suberate (DSS, Sigma, St. Louis, MO, United States), and the samples were incubated at room temperature for 30 min with rotation and centrifuged at 6,000 × g and 4°C for 15 min. The cross-linked pellets were resuspended in 30 μl of sample buffer, boiled and then analyzed by immunoblotting.

For the ASC speck assay, BMDMs were stimulated to induce NLRP3 inflammasome activation, fixed with 4% PFA for 30 min and permeabilized with 0.5% Triton X-100 for 10 min. The BMDMs were incubated with anti-ASC antibody overnight at 4°C and then stained with CoraLite488-conjugated anti-mouse IgG antibody (1:100, Proteintech, Wuhan, China) for 1 h. The nuclei were stained with Hoechst 33342 for 15 min.

### Immunoprecipitation (IP) Assay

The cell lysates were incubated with anti-ASC antibody and Protein A/G plus agarose overnight at 4°C. The proteins bound by antibody were pulled down by Protein A/G beads and subjected to immunoblotting analysis.

### Measurement of Intracellular ROS Generation

BMDMs or PMA-differentiated THP-1 cells were cultured in a 6-well plate, treated with various concentrations of KM for 1 h and stimulated with 300 ng/ml LPS for 3 h and then with 2.5 mM ATP for 1 h. After cultivation, the supernatant was removed, and the cells were washed with PBS. The cells were then incubated with 10 μM fluorescent probes and 2,7-dichlorodi-hydrofluorescein diacetate (DCFH-DA, S0033S, Beyotime, Shanghai, China) for 30 min at 37°C and immediately washed 3 times with 1 ml of PBS. Fluorescence images were captured with a multifunctional fluorescence microscope.

For the flow cytometry assay, BMDMs were treated with 200 μM KM with or without 10 mM H_2_O_2_ for 1 h and stimulated with 300 ng/ml LPS for 3 h and then with 2.5 mM ATP for 1 h. The cells were subsequently incubated with DCFH-DA probes, washed with 1 ml of PBS and resuspended in PBS. Flow cytometric analysis was performed with the CytoFLEX platform. At least 10,000 events were analyzed, and the data were analyzed using FlowJo statistical software.

### Statistical Analysis

The data are presented as the mean ± standard deviation (SD). The normality of the distribution of the data and the homogeneity of their variance were assessed, and one-way analysis of variance (ANOVA) with Bonferroni analysis was then used to compare the means from the different groups by GraphPad Prism 7.0 (GraphPad Software, SanDiego, CA, United States). The data were considered statistically significant if *p* < 0.05.

## Results

### KM Attenuates IL-1β Production in Macrophages

**FIGURE 2:**
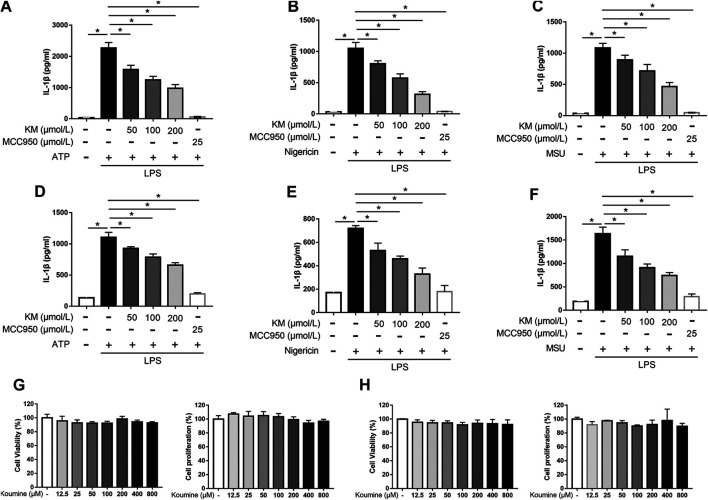
Koumine (KM) attenuates IL-1β production induced by multiple triggers in macrophages. **(A–C)** BMDMs were pretreated with KM or MCC950 for 1 h and then incubated with LPS (300 ng/ml) for 3 h and ATP (5 mM) for 1 h **(A)**, nigericin (10 μM) for 1 h **(B)** or MSU (150 μg/ml) for 6 h **(C)**. **(D–F)** PMA-differentiated THP-1 macrophages were pretreated with KM or MCC950 for 1 h and then incubated with LPS (300 ng/ml) for 3 h and ATP (5 mM) for 1 h **(D)**, nigericin (10 μM) for 30 min **(E)** or MSU (150 μg/ml) for 6 h **(F)**. The release of IL-1β in the culture supernatants was analyzed by ELISA. **(G,H)** BMDMs or PMA-differentiated THP-1 macrophages were incubated with KM for 24 h. The cell viability was detected by the MTT assay, and cell proliferation was detected by the CCK-8 assay. Mean ± SD of three independent experiments are shown. *p < 0.05.

The structure of KM is shown in Figure 1. BMDMs were successfully isolated from bone marrow progenitors and then purified by culture in BM medium for 7 days (Supplementary Figure S1). To investigate whether KM affects NLRP3 inflammasome activation, we induced BMDM macrophages with three stimuli (ATP, nigericin, and MSU) for the indicated times, as previously described (Swanson et al., 2019). The results showed that LPS plus any of the three stimuli promoted a robust increase in IL-1β secretion in the culture supernatants, and NLRP3 inflammasome inhibitor MCC950 potently inhibited IL-1β production (Figures 2A–C). Moreover, the pretreatment of cells with KM suppressed the release of IL-1β in a dose-dependent manner (Figures 2A–C). The inhibitory action of KM on IL-1β secretion from PMA-differentiated THP-1 macrophages was further confirmed (Figures 2D–F). MTT and CCK-8 assays were conducted to exclude the cytotoxicity of KM to macrophages. The results indicated that KM (50, 100, and 200 μM) did not affect the vitality and proliferation of BMDMs (Figure 2G) or PMA-differentiated THP-1 macrophages (Figure 2H), which suggests that the inhibitory effect on IL-1β production was not due to cytotoxicity.

### KM Attenuates IL-1β Production in MSU-Induced Peritonitis

**FIGURE 3:**
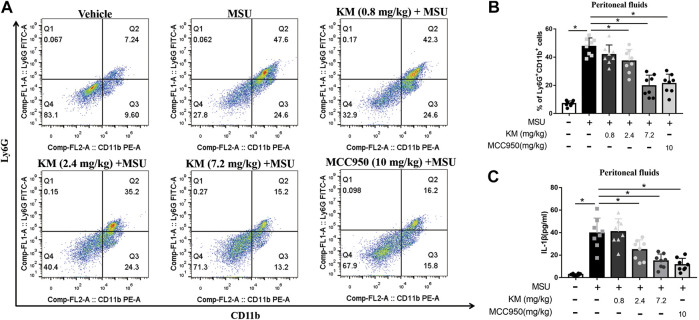
Koumine (KM) attenuates MSU-induced NLRP3 inflammasome activation *in vivo*. C57BL/6 mice were intraperitoneally pretreated with KM (0.8, 2.4, and 7.2 mg/kg), MCC950 (10 mg/kg) or vehicle for 1 h and then given an intraperitoneal injection of MSU crystals (1 mg dissolved in 500 µl of PBS). Six hours later, **(A,B)** the frequency of CD11b+Ly6G+ neutrophils retrieved from the peritoneal lavage fluid was analyzed by flow cytometry. **(C)** The amounts of IL-1β in the lavage fluid were quantified by ELISA. The data are representative of those obtained from eight mice in each treatment group. The graph presents the mean ± SD and each symbol represents an individual mouse. *p < 0.05.

Because KM inhibited IL-1β production upon NLRP3 inflammasome activation *in vitro*, we further examined the activity of KM *in vivo*. A mouse model of MSU-induced peritonitis characterized by massive neutrophil influx and IL-1β production was established to define the suppressive effects of KM on the NLRP3 inflammasome *in vivo* (Martinon et al., 2006; Jiang et al., 2020). Peritoneal exudate cells were collected, and the MSU-induced recruitment of inflammatory cells was analyzed by flow cytometry. The results showed that the MSU-induced recruitment of CD11b^+^/Ly6G^+^ neutrophils was strongly inhibited by KM or MCC950 (Figures 3A,B). We consequently analyzed the MSU-induced production of IL-1β in the lavage fluid and found that the MSU challenge greatly upregulated the IL-1β secretion level in the lavage fluid and that this upregulation was potently suppressed by KM and MCC950 pretreatment (Figure 3C). These results indicate that KM suppresses NLRP3 inflammasome-induced IL-1β production *in vitro*.

### KM Inhibits NLRP3 Inflammasome Activation

To verify whether the inhibition of IL-1β secretion by KM contributed to the inhibition of the NLRP3 inflammasome, we further investigated the effects of KM in BMDMs and PMA-differentiated THP-1 stimulated with activators of the NLRP3 inflammasome. LPS can induce the expression of NLRP3, pro-IL-1β, NLRP3 inflammasome activators (ATP, nigericin, or MSU) further lead to the assembly of the inflammasome protein complex, which cleaves pro-caspase-1 and pro-IL-1β to activate caspase-1 (p20 and p10) and mature IL-1β (p17), respectively (Bauernfeind et al., 2009; Huang et al., 2018).

**FIGURE 4:**
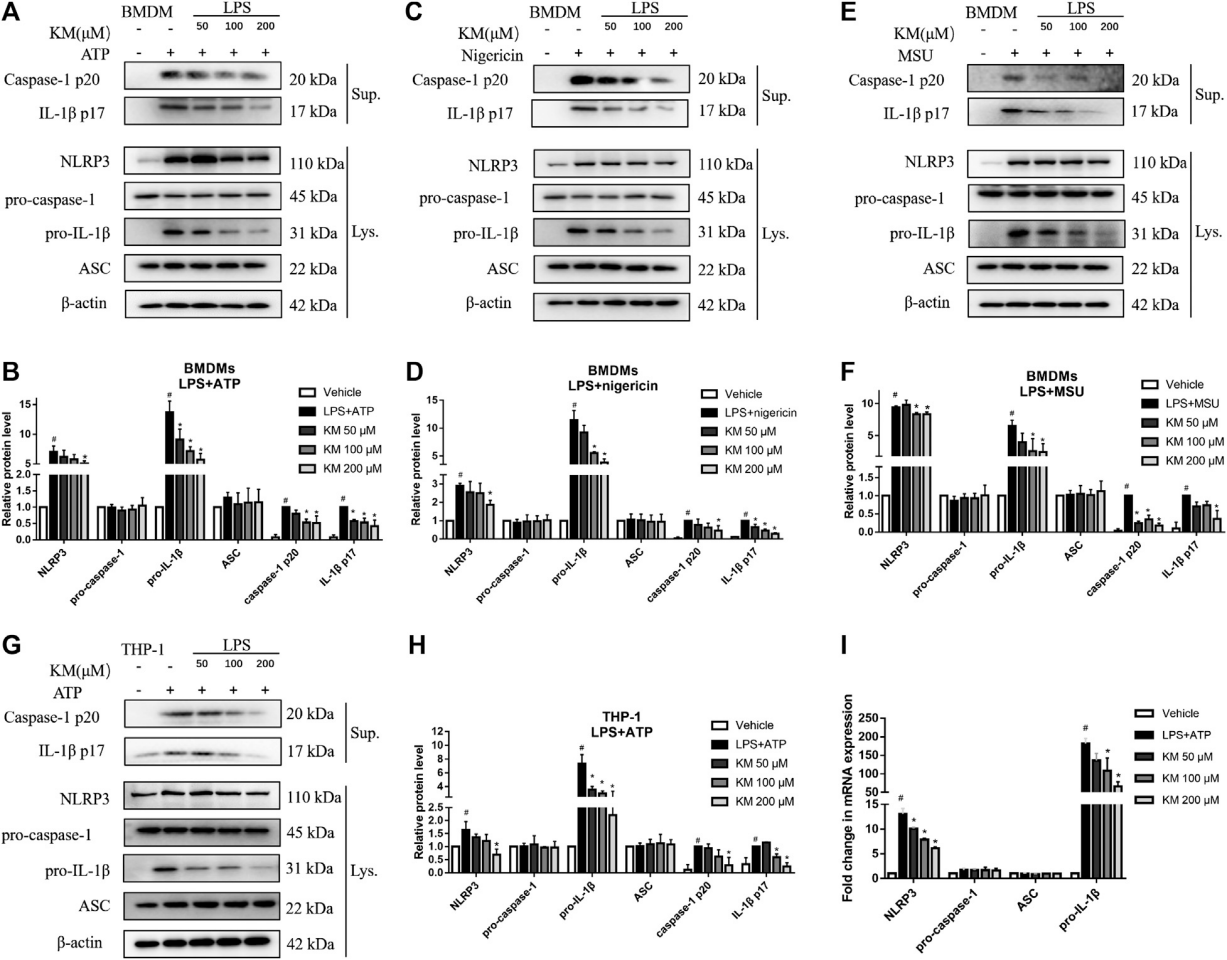
Koumine (KM) inhibits NLRP3 inflammasome activation in macrophages. **(A–F)** BMDMs were pretreated with KM for 1 h and then incubated with LPS (300 ng/ml) for 3 h and ATP (5 mM) for 1 h **(A,B)**, nigericin (10 μM) for 1 h **(C,D)** or MSU (150 μg/ml) for 6 h **(E,F)**. **(G,H)** PMA-differentiated THP-1 macrophages were pretreated with KM for 1 h and then incubated with LPS (300 ng/ml) for 3 h and ATP (5 mM) for 1 h. Supernatants (Sup.) and cell extracts (Lys.) were analyzed by immunoblotting. **(I)** The mRNA levels of NLRP3, pro-caspase-1, ASC, and pro-IL-1β in **(A)** were analyzed by RT-qPCR. Mean ± SD of three independent experiments are shown. #*p* < 0.05 vs. vehicle group, *p < 0.05 vs. LPS plus ATP, nigericin or MSU group.

Immunoblotting and RT-qPCR analysis showed that the expression of NLRP3 and pro-IL-1β was significantly increased in macrophages stimulated with LPS, which were significantly inhibited by KM pretreatment. Meanwhile, MCC950 had no effect on LPS-induced NLRP3 and pro-IL-1β expression (Supplementary Figure S2), suggesting that the inhibition mechanism of KM-induced NLRP3 inflammasome was different from MCC950. We then investigated how KM inhibited NLRP3 inflammasome activation. Immunoblotting results showed that LPS and multiple NLRP3 inflammasome activators stimulated cells express high levels of NLRP3 and pro-IL-1β (Figures 4A–H). KM did not affect the expression of pro-caspase-1 and ASC but markedly decreased the protein levels of NLRP3 and pro-IL-1β (Figures 4A–H). The upregulation and activation of NLRP3 promoted caspase-1 activation and thereby induced the processing of mature IL-1β (Bauernfeind et al., 2009). Upon activators treatment, both active caspase-1 p20 and mature IL-1β p17 were released into the culture supernatant. Interestingly, KM robustly suppressed the release of both active caspase-1 p20 and mature IL-1β p17 (Figures 4A–H). Moreover, the RT-qPCR results showed that KM substantially inhibited the LPS+ATP-induced elevation of the mRNA levels of NLRP3 and pro-IL-1β in BMDMs but has no effect on pro-caspase-1 and ASC (Figure 4I). Taken together, these results indicate that KM inhibits NLRP3 inflammasome activation.

### KM Inhibits the Activation of NF-κB Signaling

**FIGURE 5:**
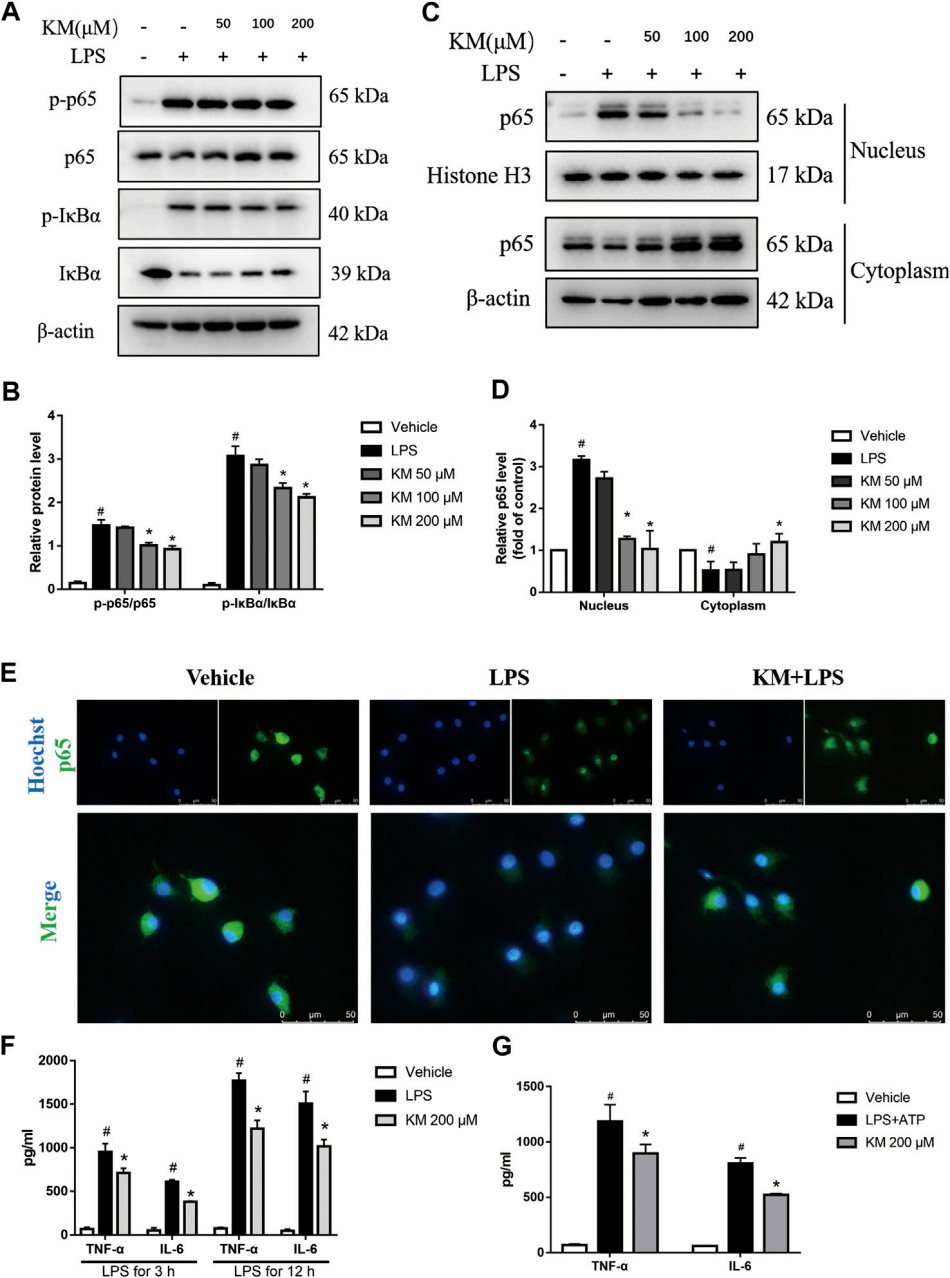
Koumine (KM) prevents NF-κB activation in BMDMs stimulated by LPS. BMDMs were pretreated with various concentrations of KM for 1 h and then incubated with LPS (300 ng/ml) for 1 h. **(A,B)** The protein levels of total p65, IκBα and their phosphorylated forms (p-p65 and p-IκBα) were analyzed by western blotting. **(C,D)** p65 nuclear translocation was determined by Western Blot. Mean ± SD of three independent experiments are shown. #*p* < 0.05 vs. vehicle group, *p < 0.05 vs. LPS group. **(E)** The nuclear location of p65 in BMDM macrophages was visualized by immunofluorescence analysis with an anti-p65 (green) antibody. The nuclei (blue) were stained with Hoechst 33324. KM, 200 μM, Scale bars, 50 μm. **(F)** BMDMs were pretreated with KM for 1 h and then incubated with LPS (300 ng/ml) for 3 or 12 h. The release of TNF-α and IL-6 in the culture supernatants was analyzed by ELISA. Mean ± SD of three independent experiments are shown. #*p* < 0.05 vs. vehicle group, *p < 0.05 vs. LPS group. **(G)** BMDMs were pretreated with KM for 1 h and then incubated with LPS (300 ng/ml) for 3 h and ATP (5 mM) for 1 h. The release of TNF-α and IL-6 in the culture supernatants was analyzed by ELISA. Mean ± SD of three independent experiments are shown. #*p* < 0.05 vs. vehicle group, *p < 0.05 vs. LPS plus ATP group.

Emerging evidence has demonstrated that NF-κB plays a pivotal role in the inflammatory response and NLRP3 inflammasome activation (Liu et al., 2017a; Mahmoud et al., 2019). Moreover, KM is reportedly effective in inhibiting NF-κB signaling (Yuan et al., 2019a; Wu et al., 2020). To investigate whether the KM-induced suppression of NLRP3 inflammasome activation depends on the NF-κB pathway and affects LPS-induced priming, we analyzed the protein levels and nuclear localization of molecules related to the NF-κB pathway. As shown in Figures 5A,B, the pretreatment of BMDMs with KM markedly suppressed the LPS-induced phosphorylation of IκBα and p65. We also found that KM effectively inhibited the nuclear localization of p65 (Figures 5C–E). The inhibitory action of KM on p65 nuclear localization from PMA-differentiated THP-1 macrophages was further confirmed (Supplementary Figure S3). These data supported the previous finding that KM suppressed both the protein and mRNA levels of NLRP3 pro-IL-1β (Figure 4; Supplementary Figure S2). We then further examined the effect of KM on TNF-α and IL-6 expression, which is dependent on NF-κB activation (Wang et al., 2019). ELISA results showed that KM reduced the secretion of TNF-α and IL-6 from BMDMs stimulated with LPS (Figure 5F). Additionally, KM decreased the production of TNF-α and IL-6 (Figure 5G) in BMDMs stimulated with LPS plus ATP as well as the production of IL-1β (Figure 2A). These results suggest that KM decreases LPS-primed NLRP3 expression and inhibits NLRP3 inflammasome activation associated with suppressing NF-κB activation.

### KM Hampers the Assembly of the NLRP3 Inflammasome

**FIGURE 6:**
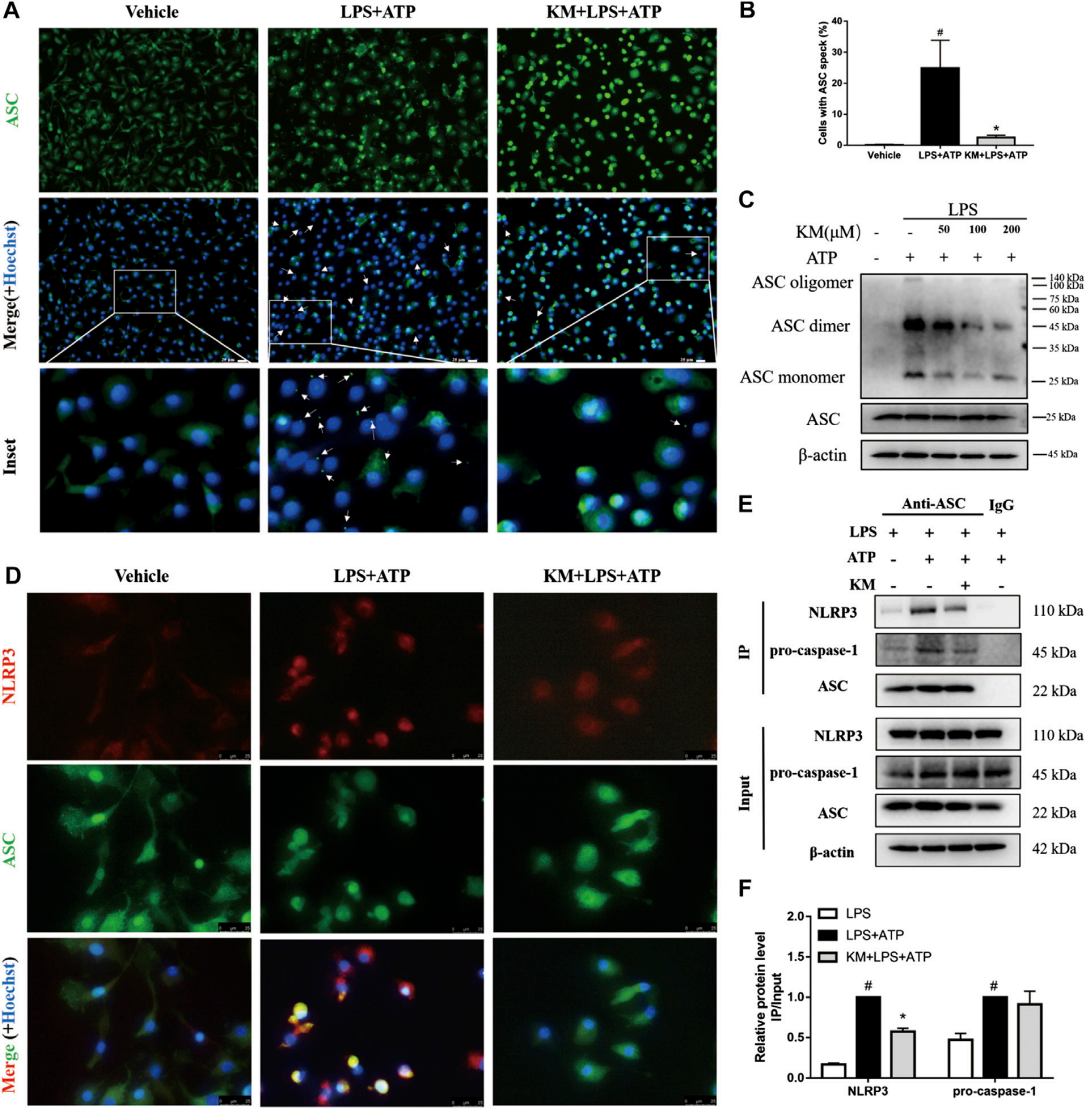
Koumine (KM) blocks NLRP3 inflammasome assembly in BMDMs. BMDMs were pretreated with KM (200 μM) for 1 h and then incubated with LPS (300 ng/ml) for 3 h and ATP (5 mM) for 1 h. **(A)** Representative immunofluorescence images of ASC speck formation are shown. The white arrows indicate ASC specks (green), and the nuclei (blue) were stained with Hoechst 33324. Scale bars, 20 μm. **(B)** Quantification of cells with ASC specks in six random images. **(C)** ASC oligomerization in Triton-X100-insoluble pellets cross-linked with DSS was analyzed by western blotting. **(D)** The interaction between NLRP3 (red) and ASC (green) in BMDMs were assayed by immunofluorescence. Scale bars, 25 μm. **(E,F)** Cell lysates from cultured cells were analyzed by immunoprecipitation using an antibody against ASC and then analyzed by immunoblotting, and IgG was used as the isotype control. Mean ± SD of three independent experiments are shown. #*p* < 0.05 vs. LPS group, *p < 0.05 vs. LPS plus ATP group.

During NLRP3 inflammasome activation, ASC protein oligomerization is critical for subsequent caspase-1 activation (Dick et al., 2016; Pan et al., 2020). Consistent with the inhibitory effects of KM on caspase-1 activation and IL-1β secretion (Figures 2A–F, 4A–H, the immunofluorescence results showed that KM markedly reduced the formation of ASC specks in response to stimulation with LPS plus ATP (Figures 6A,B). Furthermore, we crosslinked ASC using DSS and analyzed ASC oligomerization by immunoblotting (Fernandes-Alnemri and Alnemri, 2008; Green et al., 2018). Similar results were obtained: KM suppressed ASC oligomerization in response to stimulation with LPS plus ATP (Figure 6C), LPS plus nigericin, and LPS plus MSU (Supplementary Figure S4A). We then investigated whether KM inhibited the assembly of the NLRP3 inflammasome. Recruitment of ASC to NLRP3 has been proposed as an essential step for NLRP3 inflammasome assembly (Martinon et al., 2009; He et al., 2018). Immunofluorescence results revealed that KM inhibited the association of ASC with NLRP3 (Figure 6D). The inhibitory action of KM on the association of ASC with NLRP3 from PMA-differentiated THP-1 macrophages was further confirmed (Supplementary Figure S4B). Immunoprecipitation analysis further demonstrated that KM affected the binding between ASC and NLRP3 but slightly affected the binding between ASC and pro-caspase1 (Figures 6E,F). Thus, these results indicate that KM blocks NLRP3-ASC complex formation and further inhibits NLRP3 inflammasome activation.

### KM Inhibits ROS Generation

**FIGURE 7:**
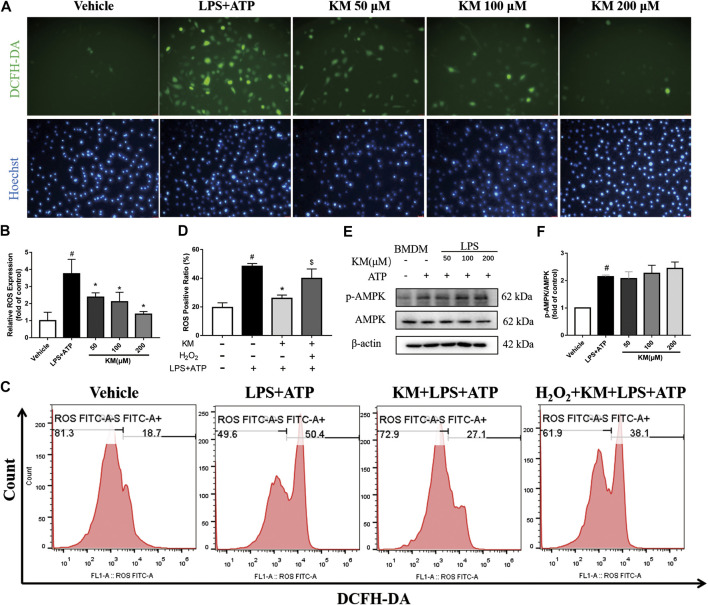
Koumine (KM) inhibits ROS generation triggered by LPS and ATP. **(A,B)** BMDMs were pretreated with various concentrations of KM for 1 h and then incubated with LPS (300 ng/ml) for 3 h and ATP (5 mM) for 1 h. Intracellular ROS were labeled with the DCFH-DA probe and examined by fluorescence microscopy. Scale bars, 25 μm. Mean ± SD of three independent experiments are shown. #*p* < 0.05 vs. vehicle group, *p < 0.05 vs. LPS plus ATP group. **(C,D)** BMDMs were pretreated with KM (200 μM) for 1 h and then incubated with LPS (300 ng/ml) for 3 h and ATP (5 mM) for 1 h. H_2_O_2_ (10 mM) was added to cells at the same time as KM. Intracellular ROS were labeled with a DCFH-DA probe and examined by flow cytometry. #*p* < 0.05 vs. vehicle group, *p < 0.05 vs. LPS plus ATP group, $*p* < 0.05 vs. KM plus LPS and ATP group. **(E,F)** The protein levels of total AMPK and its phosphorylated forms (p-AMPK) were analyzed by western blotting. Mean ± SD of three independent experiments are shown. #*p* < 0.05 vs. vehicle group.

The above-described findings indicated that the activities of both NF-κB and NLRP3 are suppressed by KM, which suggests that an upstream regulator of these two signaling pathways might mediate these effects. ROS are signaling intermediates that can drive both NLRP3 inflammasome and NF-κB activation (Tschopp and Schroder, 2010; Wu et al., 2018; Wang et al., 2020). Herein, we measured production of ROS in marophages stimulated with LPS plus ATP. Both fluorescence microscopy analysis (Figures 7A,B; Supplementary Figure S5) and flow cytometry analysis (Figures 7C,D) showed that high levels of ROS were produced after treatment with LPS plus ATP and that KM significantly reduced the production of ROS. Exogenous H_2_O_2_ can promote ROS (Liu et al., 2017b). We found that the effect of KM on suppressing the release of ROS in response to stimulation with LPS plus ATP was reversed by H_2_O_2_ treatment. To elucidate the mechanism underlying the inhibitory effects of KM on ROS, we investigated whether KM disturbed adenosine monophosphate-activated protein kinase (AMPK) activation because AMPK regulates the production of ROS (Wang et al., 2010). Although KM upregulated the ATP-induced phosphorylation of AMPK slightly, but no statistical difference was found between the LPS+ATP group and KM pretreatment group (Figures 7E,F). These data suggest that KM inhibits NLRP3 inflammasome activation by inhibiting ROS generation may independent of the AMPK pathway.

## Discussion

Although many studies have attempted to explore the therapeutic effect and mechanism of KM in diseases (Qiu et al., 2015; Yang et al., 2016; Yuan et al., 2016b; Chen et al., 2017; Jin et al., 2018a; Jin et al., 2018b; Jin et al., 2019; Yuan et al., 2019a; Yuan et al., 2019b; Yue et al., 2019), its effect on NLRP3 inflammasome activation and the underlying mechanism remain unknown. In this study, we identified KM as a broad-spectrum inhibitor of NLRP3 inflammasome activation that inhibited NLRP3 inflammasome activation both *in vitro* and *in vivo*. KM hampered LPS priming and NLRP3 inflammasome assembly in macrophages. Furthermore, our results showed that this inhibitory action of KM might occur by mediating the elimination of ROS. In summary, our results demonstrate a previously unrecognized novel mechanism through which KM suppresses inflammation.

Inflammation is a tightly regulated process, and the innate immune system must integrate multiple signals to determine whether a response will be initiated. This coordination is particularly apparent in the pathway controlling the proinflammatory cytokine IL-1β (Mishra et al., 2013). The transcription of pro-IL-1β is induced through TLRs, which recognize conserved microbial structures, such as LPS. The subsequent processing of pro-IL-1β into its active form is mediated by inflammasomes, particularly the NLRP3 inflammasome (Lamkanfi and Dixit, 2009). The NLRP3 inflammasome can be activated by a wide range of activators, including ATP, nigericin and MSU, in LPS-primed macrophages (Swanson et al., 2019). We found that KM inhibited the production of IL-1β in BMDMs and PMA-differentiated THP-1 macrophages after simulation with LPS plus ATP, nigericin and MSU. Further study found that KM also robustly inhibited neutrophil infiltration and IL-1β production in an MSU-induced peritonitis model. MSU-induced peritonitis is regarded as a typical animal model of NLRP3-IL-1β axis-dependent inflammation (Martinon et al., 2006). These results suggest that the anti-inflammatory effect of KM might occur through the regulation of NLRP3 inflammasome activation.

The activation of the NLRP3 inflammasome requires two steps: 1) LPS or another TLR agonist first induces NLRP3 and pro-IL-1β synthesis in an NF-κB-dependent manner, and 2) a second signal then leads to NLRP3 inflammasome assembly mediated cleaved caspase-1 and IL-1β secretion (Guo et al., 2018). We observed that KM decreased NLRP3 and pro-IL-1β protein expression and inhibited the production of IL-1β stimulated by ATP, nigericin and MSU in LPS-primed macrophages. Upon LPS stimulation, IkBα is phosphorylated and thus degraded by ubiquitin-dependent proteasomes, which further allows p65 phosphorylation and disassembly from the heterodimer. Subsequently, p65 translocates to the nucleus and upregulates NLRP3 and IL-1β mRNA expression (Afonina et al., 2017). Researchers have found that KM can inhibit NF-κB activation and mediate the expression of proinflammatory cytokines in RAW264.7, IPEC-J2 and hepatocellular carcinoma cells (Yuan et al., 2016b; Yuan et al., 2019a; Wu et al., 2020). Our study confirmed that the pretreatment of BMDMs with KM reduced the phosphorylation of IκBα and p65 and suppressed the translocation of p65 to the nucleus and the secretion of TNF-α and IL-6. These results indicate that the pretreatment of BMDMs with KM inhibits the transcriptional upregulation of NLRP3 and pro-IL-1β by interfering with NF-κB activity. ASC is critical for the assembly and subsequent activation of the NLRP3 inflammasome by yielding ASC dimers and/or oligomers that localize to a detergent-insoluble fraction in the cells (Zhou et al., 2011; Jiang et al., 2020). Immunofluorescence and immunoblotting analyses confirmed that treatment with LPS plus ATP resulted in the formation of ASC specks and oligomerization, and these effects were significantly reduced in the presence of KM. These results showed that KM might act in the assembly step to inhibit NLRP3 inflammasome activation. Consistent with this hypothesis, our further studies showed that KM hampered the formation of NLRP3-ASC complexes and suppressed caspase-1 processing.

An interesting question is how KM inhibits NRP3 inflammasome activation induced by multiple stimuli. Because KM inhibits both LPS-induced NF-κB expression and ATP-induced NRP3 activation, these independent pathways that are targeted by KM might have a common feature. ATP, nigericin and MSU crystals serve as activators of the NLRP3 inflammasome through different mechanisms. Extracellular ATP triggers P2X7-dependent pore formation, which induces potassium efflux and causes NLRP3 activation (Karmakar et al., 2016). Nigericin is a lipophilic ionophore that directly functions as a K+/H+ exchanger upon partitioning into the plasma membrane or intracellular organelles, which induces NLRP3 activation (Katsnelson et al., 2015). MSU crystals lead to lysosomal damage and result in NRP3 activation (Hornung et al., 2008). Recent studies have shown that ROS generation is a common trigger of NLRP3 inflammasome activation (Dostert et al., 2008; Tschopp and Schroder, 2010; Zhou et al., 2011; Sho and Xu, 2019). A number of reports have shown that ROS have the potential to activate NF-κB (Nakajima and Kitamura, 2013; Jiang et al., 2018; Long et al., 2020). Under LPS challenge, TLR4 binds to nicotinamide adenine dinucleotide phosphate (NADPH) oxidase 4 and further promotes the production of ROS-induced NF-κB activation (Park et al., 2004). KM is an antioxidant agent that maintains the mitochondrial membrane potential in porcine intestinal epithelial cells (Yuan et al., 2019b). We confirmed that KM can exert NLRP3 inhibitory activity by eliminating ROS generation in response to stimulation with LPS plus ATP. Our results revealed that ROS production in LPS-primed BMDMs was significantly upregulated by ATP treatment and that this effect was accompanied by obvious upregulation of NLRP3 and pro-IL-1β expression, which was consistent with ASC speck formation and NLRP3 inflammasome assembly. However, KM pretreatment suppressed these transformations induced by LPS plus ATP. In addition, H_2_O_2_, an ROS promoter, rescues the inhibitory effect of KM in ROS production in these cells. These results indicated that the KM-induced suppression of NLRP3 inflammasome activation is related to regulation of the ROS/NF-κB/NLRP3 axis.

Several of our studies have explored the therapeutic effects of KM on various disease models. Jin et al. demonstrated that KM attenuates chronic constriction injury (CCI) induced neuropathic pain by decreasing astrocyte-mediated neuroinflammation and enhancing autophagy in rats (Jin et al., 2018b). Yue et al. revealed that KM exhibits positive efficacy on modulating different subtypes of helper T cells and preventing nonalcoholic fatty liver disease (Yue et al., 2019). Chen et al. compared the effects of KM and diazepam in an open-field test of mice and a vogel conflict test of rats and found that KM exhibits anxiolytic properties without inducing adverse neurological effects (Chen et al., 2017). Yang et al. confirmed that KM inhibits the paw volume, AI score, mechanical allodynia, and IL-1β and TNF-α secretion in both adjuvant-induced and collagen-induced rheumatoid arthritis models (Yang et al., 2016). In this study, we further found that KM potently inhibited IL-1β production and neutrophil influx in MSU peritonitis. Interestingly, all the above-mentioned diseases are accompanied by abnormal activation of the NLRP3 inflammasome (Xu et al., 2016; Guo et al., 2018; Mi et al., 2018; Zhang et al., 2018; Xu et al., 2019; Jiang et al., 2020). Our results support the theory that the aberrant NLRP3 activation is involved in a range of both acute and chronic inflammatory conditions and linked with the development of many diseases, and it is feasible to treat these diseases by modulating NLRP3 inflammasome activation (Mangan et al., 2018). Although the role of KM in the regulation of the NLRP3 inflammasome in these diseases requires further investigation, our results highlight that KM might be used for the treatment of NLRP3-related disorders.

Here, we found that KM showed anti-inflammation effect with a relatively high concentration *in vitro* compared with MCC950, but have comparable effect *in vivo*. This may be due to differences between cell model and animal model, and KM may possess potential anti-inflammatory properties through multiple pathways *in vivo*. MSU recruits a large numbers of activated neutrophils into the peritoneal, resulting in the production of high levels of superoxide by the neutrophil NADPH oxidase. Then, uperoxide rapidly undergoes spontaneous dismutation to hydrogen peroxide and subsequently formation of highly damaging ROS (Martin et al., 2009). We deduced that the KM might also potently inhibits the ROS production in neutrophils despite further studies are needed. Although the ROS inhibitory effect of KM was defined in our study, further investigation is needed to further identify which protein is the initial target of KM. AMPK is an essential mediator of fatty acid metabolism (Steinberg and Kemp, 2009) and inhibits the expression and function of NADPH oxidase to disturb the production of ROS (Wang et al., 2010). AMPK is involved in cellular apoptosis pathways and mediates autophagic activity (Heidrich et al., 2010). Additionally, recent studies have revealed an AMPK-ROS signaling axis that regulates NLRP3 inflammasome activation (Yang et al., 2010; Wen et al., 2011; Bullon et al., 2016). Our previous study showed that KM can enhance autophagy and decrease astrocyte-mediated neuroinflammation in CCI rats (Jin et al., 2018b). We then investigated whether KM inhibits ROS production through a mechanism dependent on AMPK but found that KM could not facilitate ATP-induced AMPK phosphorylation. This result suggested that the effect of KM on ROS production and NLRP3 activation may independent of AMPK. Recent studies have shown that a translocator protein (18 kDa) (TSPO) plays an important role in modulating NOX1-dependent ROS production in the retina (Wolf et al., 2020). TSPO upregulation is concomitant with NLRP3 inflammasome activation in bipolar disorder. Previous studies proposed that the upregulation of TSPO and voltage-dependent anion channel culminates in inhibition of the mitophagy pathway, which leads to a pronounced accumulation of damaged mitochondria and excessive NLRP3-dependent inflammation (Scaini et al., 2019). Interestingly, the TSPO ligand Ro5-4864 can significantly inhibit ATP-induced NLRP3 inflammasome activation (Lee et al., 2016). In addition, our previous studies revealed that the analgesic effect of KM is inhibited by another TSPO ligand, PK11195, in a CCI induced neuropathic pain model (Jin et al., 2018a). A recently research find that uric acid drives intestinal barrier dysfunction through TSPO-mediated NLRP3 inflammasome (Lv et al., 2020). Therefore, further investigation is needed to elucidate whether KM modulates the ROS/NF-κB/NLRP3 signaling pathway via a mechanism dependent on TSPO. In addition to the NLRP3 inflammasome, caspase-1 also plays an important role in NLRP1, NLRP4 and AIM2 inflammasomes (Schroder and Tschopp, 2010). Hence, additional studies are needed to determine whether KM inhibits the activation of other inflammasomes involving caspase-1.

## Conclusion

**FIGURE 8:**
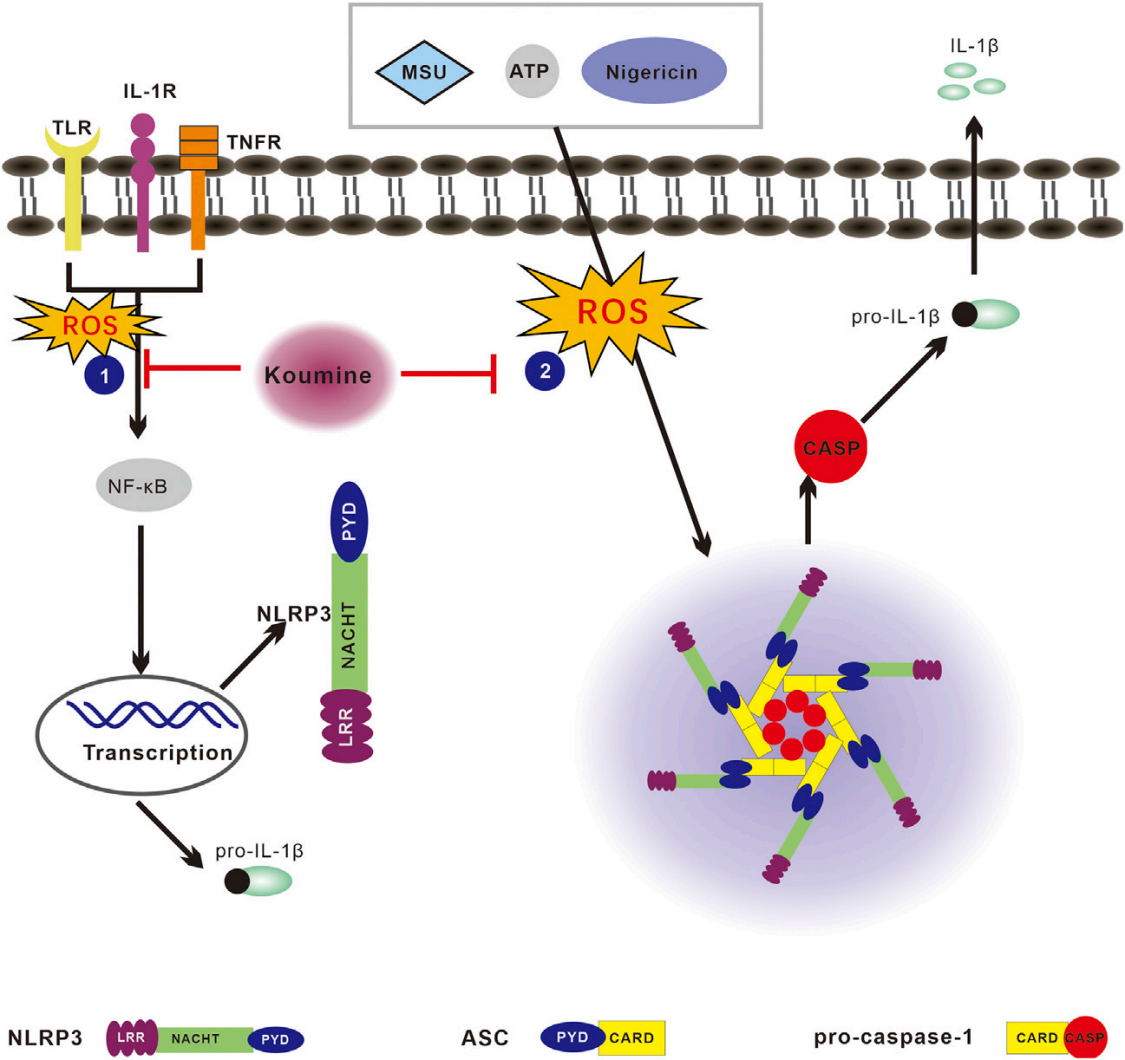
Proposed model of the effect of koumine (KM) on reactive oxygen species (ROS)/NF-κB/nod-like receptor NOD-like receptor protein 3 (NLRP3) signaling. The NLRP3 inflammasome can be activated by multiple stimuli. The priming step or “signal 1” is mediated by TLR agonists such as LPS, which results in NF-κB-dependent upregulation of NLRP3 and pro-IL-1β expression. The assembly step or “signal 2” mediated by stimulation with numerous pathogen-associated molecular patterns (PAMPs) or danger-associated molecular patterns (DAMPs), such as ATP, nigericin and MSU, promotes ASC oligomerization, leading to activation of the NLRP3 inflammasome complex. The stimulation of cells with LPS, ATP, nigericin or MSU leads to ROS production and activates both the priming and assembly steps, which ultimately leads to NLRP3 inflammasome activation. The activated NLRP3 inflammasome then cleaves pro-caspase-1 into activated caspase-1 and thereby promotes mature IL-1β secretion. KM inhibits NLRP3 inflammasome activation associated with regulating the ROS/NF-κB/NLRP3 pathway and consequently suppresses IL-1β production.

In summary, our findings demonstrate that KM inhibits NLRP3 inflammasome activation (Figure 8). KM inhibits LPS priming step through the block of p65 NF-κB activation. It also suppresses the ASC specks formation, and the NLRP3 inflammasome assembly. These effects occur associated with inhibiting ROS/NF-κB/NLRP3 inflammasome axis. This study constitutes the firms study aiming to explore the anti-inflammatory mechanism of KM on the NLRP3 inflammasome. Because the NLRP3 inflammasome is involved in a variety of diseases, KM might be a useful strategy for the treatment of NLRP3-driven diseases.

## Data Availability Statement

The raw data supporting the conclusions of this article will be made available by the authors, without undue reservation.

## Ethics Statement

The animal study was reviewed and approved by the ethics committee of Fujian Medical University.

## Author Contributions

JY, CY, and YL conceived and designed the experiments. YL, BX, HL, ZC, and HH performed the experiments. YL and BX analyzed the data. YL wrote the paper. JY and CY helped to proofread the article. All the authors contributed to the article and approved the submitted version.

## Funding

This work was supported by the National Natural Science Foundation of China (Nos. 81872879, 81773716, and 81973309), the Drug Innovation Major Project of China (No. 2018ZX09711001-003-024), the Industry-University-Research Cooperation Project of Fujian Province (No. 2017Y4007), the Joint Funds for the Innovation of Science and Technology, Fujian Province (Grant number: 2018Y9073, 2016Y9058), and the Startup Fund for Scientific Research, Fujian Medical University (No. 2018QH2014).

## Conflict of Interest

The authors declare that the research was conducted in the absence of any commercial or financial relationships that could be construed as a potential conflict of interest.
