# Supplementary material for: Koumine Suppresses IL-1β Secretion and Attenuates Inflammation Associated With Blocking ROS/NF-κB/NLRP3 Axis in Macrophages
Source: Front Pharmacol. 2021 Jan 18;11:622074. doi: 10.3389/fphar.2020.622074 (PMC7851739; doi:10.3389/fphar.2020.622074)
Supplement: Supplementary file 1 [file datasheet1.pdf]

Supplementary Figures

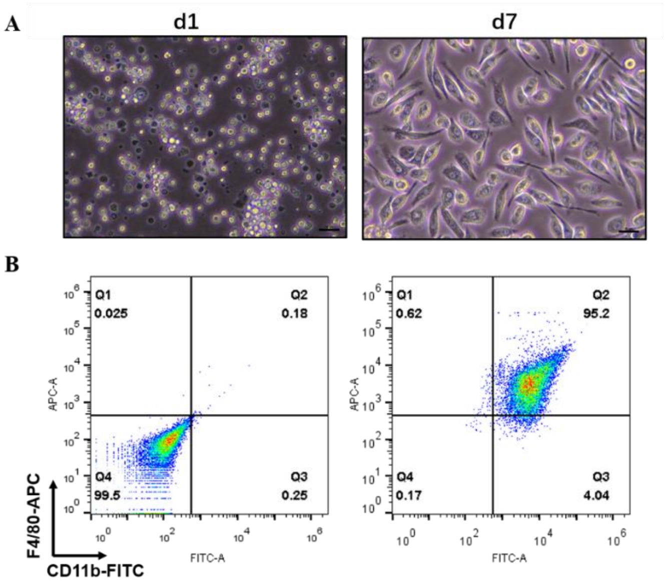

FIGURE S1 Mouse bone marrow cells differentiate into macrophages. Mouse bone marrow cells were induced by incubation in BM-medium for 7 days, and the culture medium was replaced every other day. (A) Micrographs of cells were observed using a bright-field Leica DMi8 imaging system. Scale bar, 50 μm. (B) The specific macrophage markers F4/80 and CD11b were detected by flow cytometry.

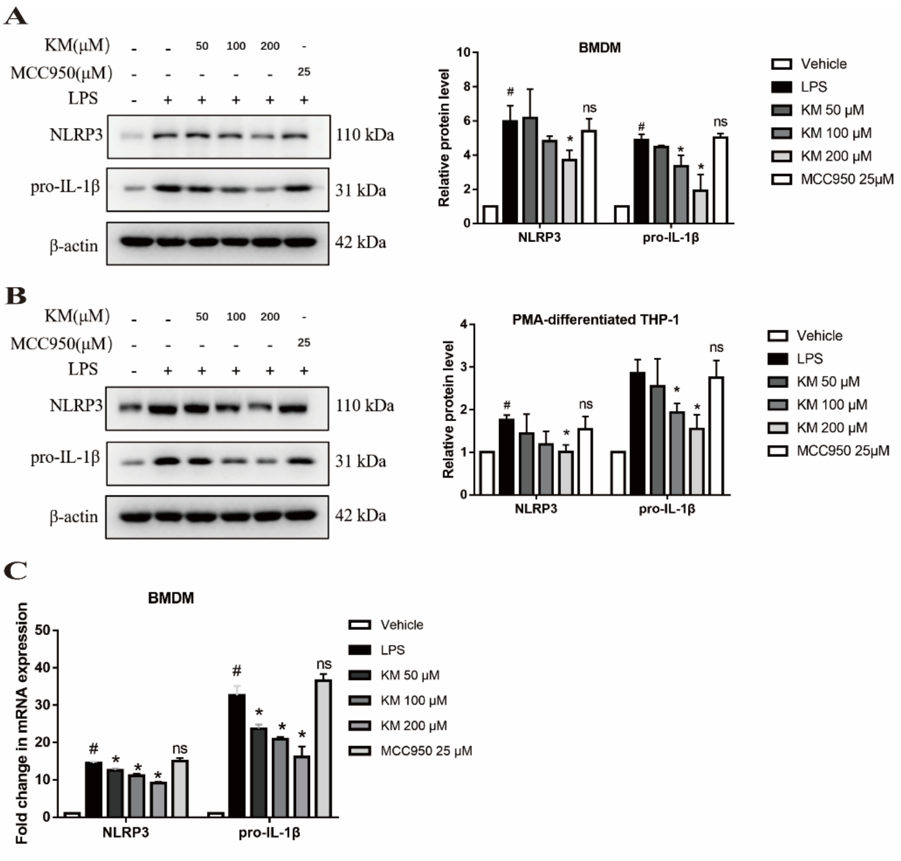

FIGURE S2 Koumine (KM) inhibits the expression of NLRP3 and pro-IL-1β stimulated by LPS in macrophages. (A) BMDMs or (B) PMA-differentiated THP-1 cells were pretreated with KM or MCC950 for 1 h and then incubated with LPS (300 ng/ml) for 3 h. Cell lyses were collected and analyzed by immunoblotting for NLRP3 and pro-IL1β. The mRNA levels of NLRP3 and pro-IL1β in (A) were analyzed by RT-qPCR. Mean ± SD of 3 independent experiments are shown. #*P* < 0.05 versus vehicle group, **P* < 0.05 versus LPS group.

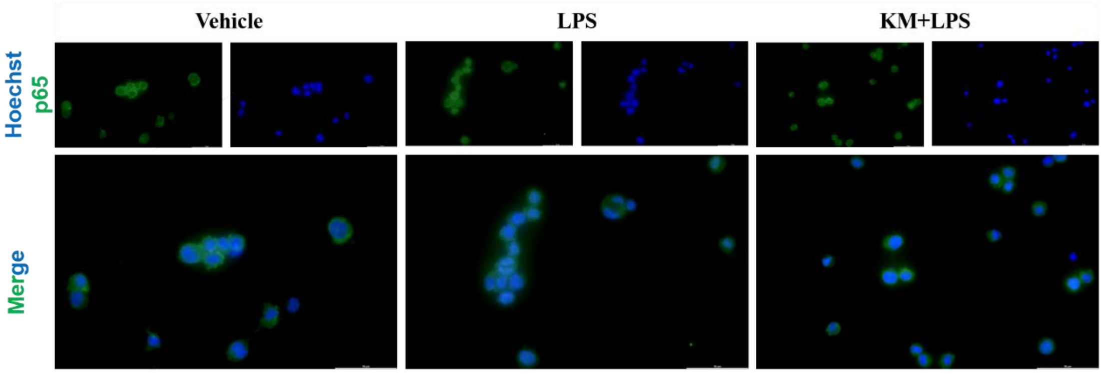

FIGURE S3 Koumine (KM) suppresses the p65 nuclear localization in PMA-differentiated THP-1 macrophages. PMA-differentiated THP-1 macrophages were pretreated with KM (200 μM) for 1 h and then incubated with LPS (300 ng/ml) for 1 h. The nuclear location of p65 in PMA-differentiated THP-1 macrophages were visualized by immunofluorescence analysis with an anti-p65 (green) antibody. The nuclei (blue) were stained with Hoechst 33324. Scale bars, 50 μm.

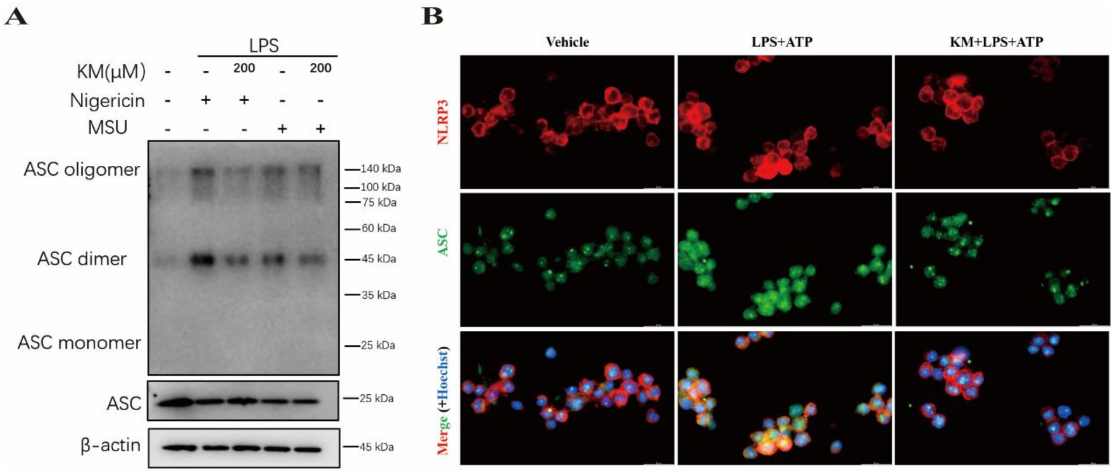

FIGURE S4 Koumine (KM) blocks NLRP3 inflammasome assembly in macrophages. (A) BMDMs were pretreated with KM (200 μM) for 1 h and then incubated with LPS (300 ng/ml) for 3 h, nigericin (10 μM) for 1 h or MSU (150 μg/ml) for 6 h. ASC oligomerization in cross-linked cytosolic pellets were analyzed by immunoblotting. (B) PMA-differentiated THP-1 macrophages were pretreated with KM (200 μM) for 1 h and then incubated with LPS (300 ng/ml) for 3 h and ATP (5 mM) for 1 h. The interaction between NLRP3 (red) and ASC (green) in cells were assayed by immunofluorescence. Scale bas, 25 μm.

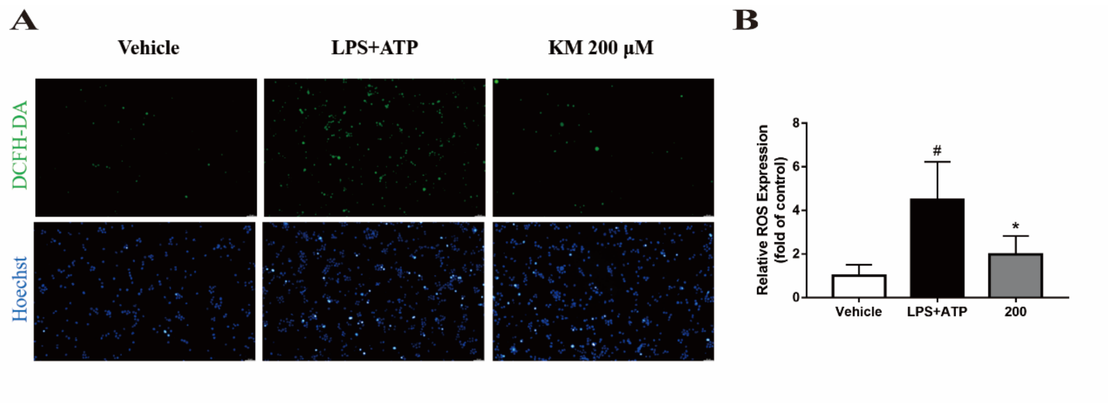

FIGURE S5 Koumine (KM) inhibits ROS generation triggered by LPS and ATP in PMA-differentiated THP-1 macrophages. PMA-differentiated THP-1 macrophages were pretreated with KM (200 μM) for 1 h and then incubated with LPS (300 ng/ml) for 3 h and ATP (5 mM) for 1 h. Intracellular ROS were labeled with the DCFH-DA probe and examined by fluorescence microscopy. Scale bas, 50 μm. Mean ± SD of 3 independent experiments are shown. #*P* < 0.05 versus vehicle group, **P* < 0.05 versus LPS plus ATP group.

The original western blots of figure 4.

FIGURE 4A

Caspase-1 p20	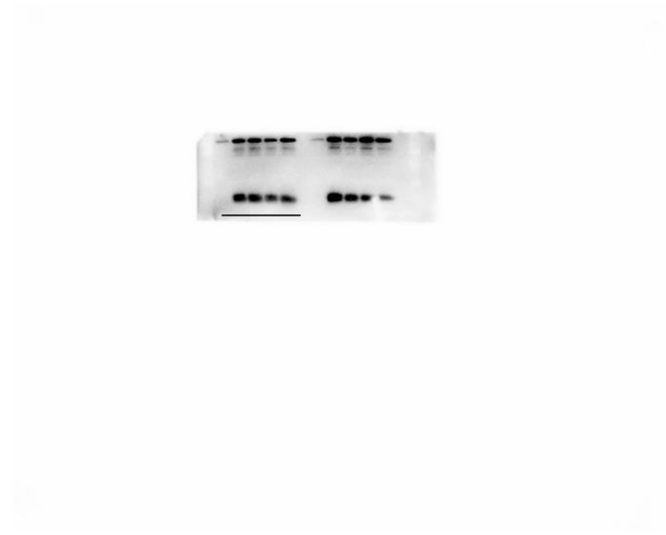
IL-1β p17	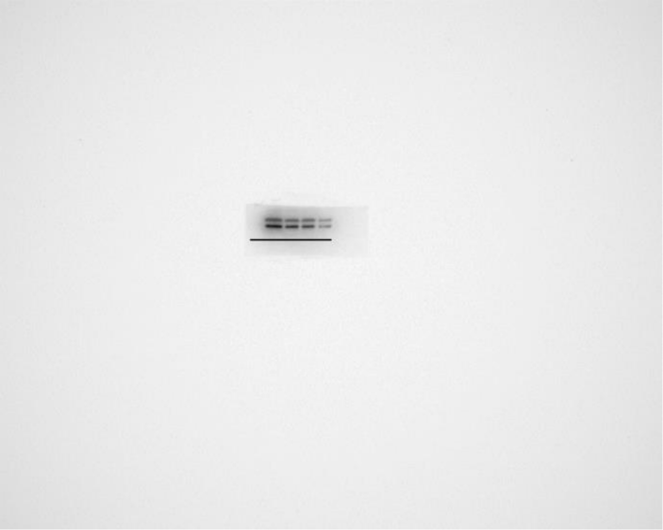
NLRP3	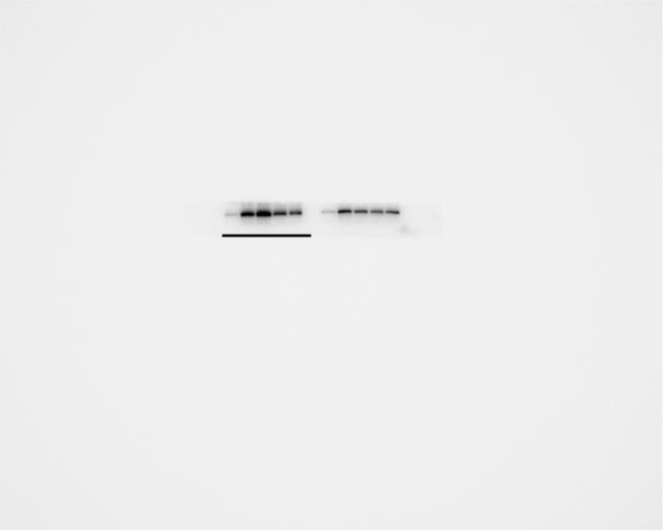

Pro-caspase-1	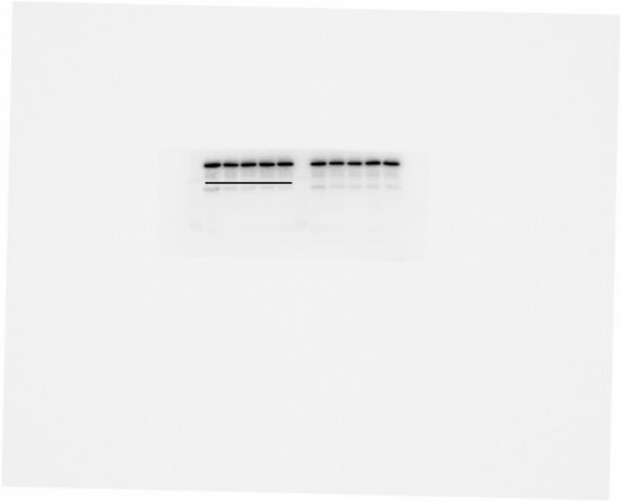
Pro-IL-1β	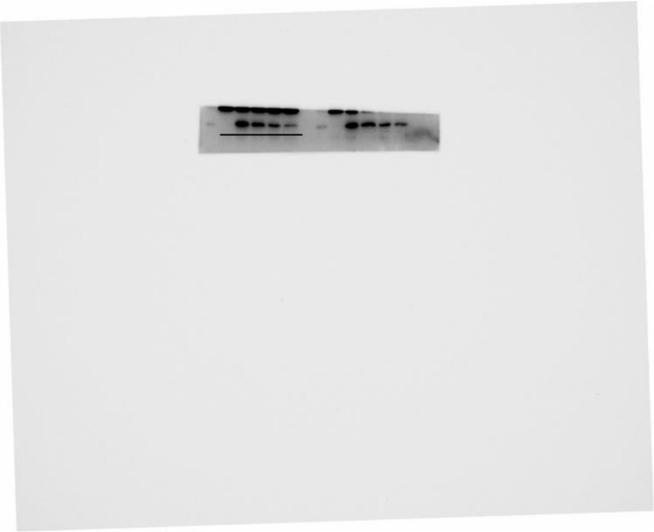
ASC	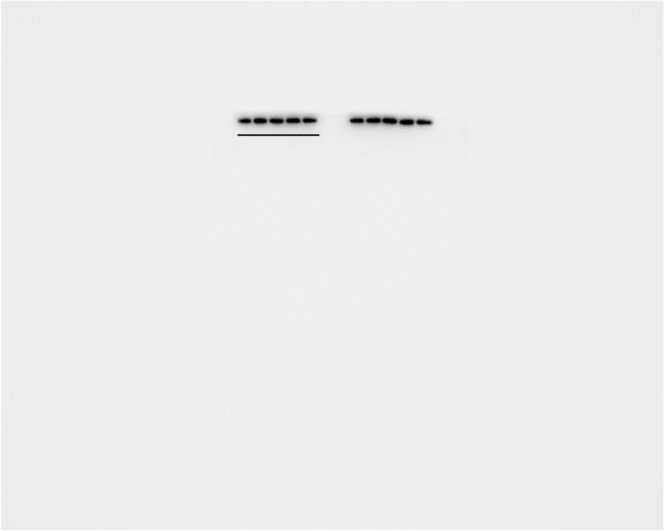

β-actin	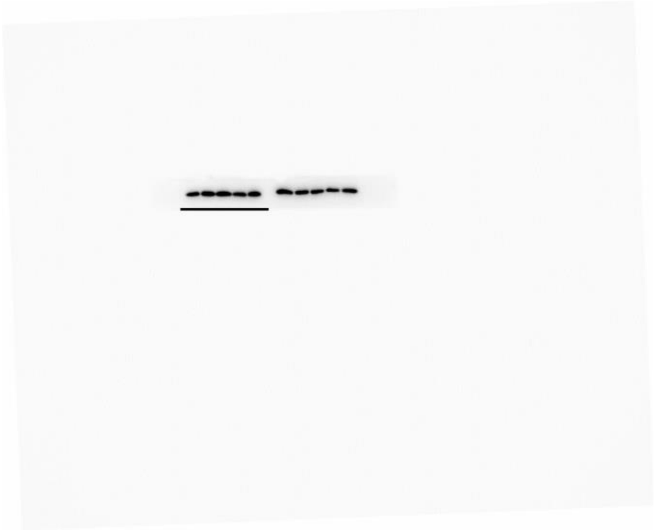

FIGURE 4C

Caspase-1 p20	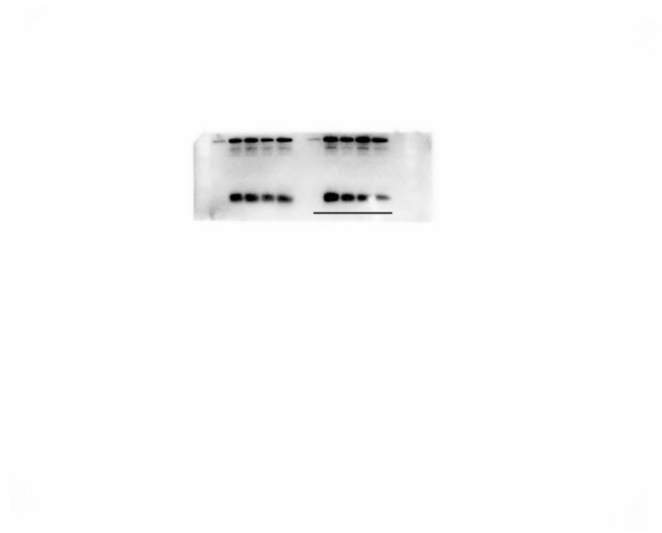
IL-1β p17	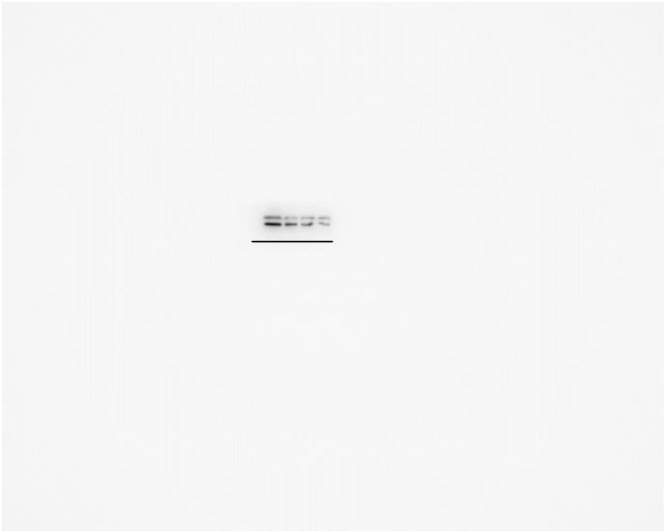

NLRP3	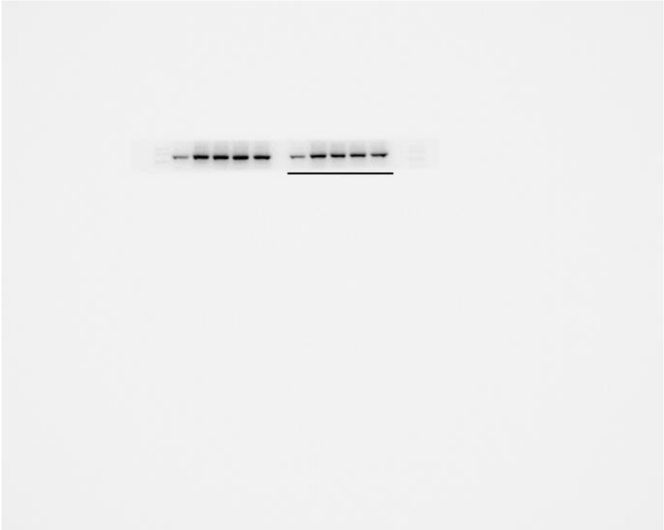
Pro-caspase-1	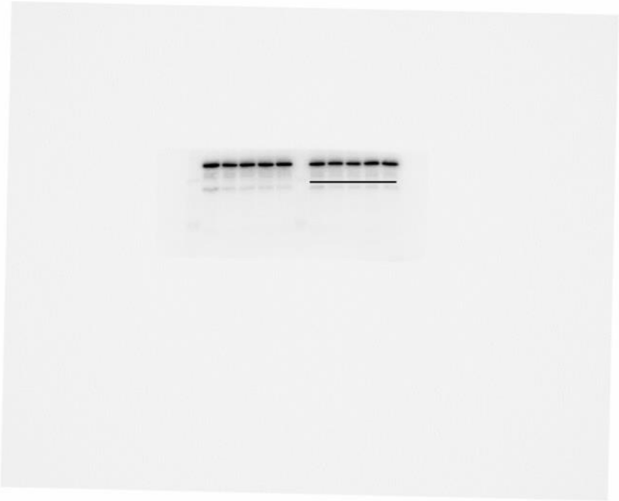
Pro-IL-1β	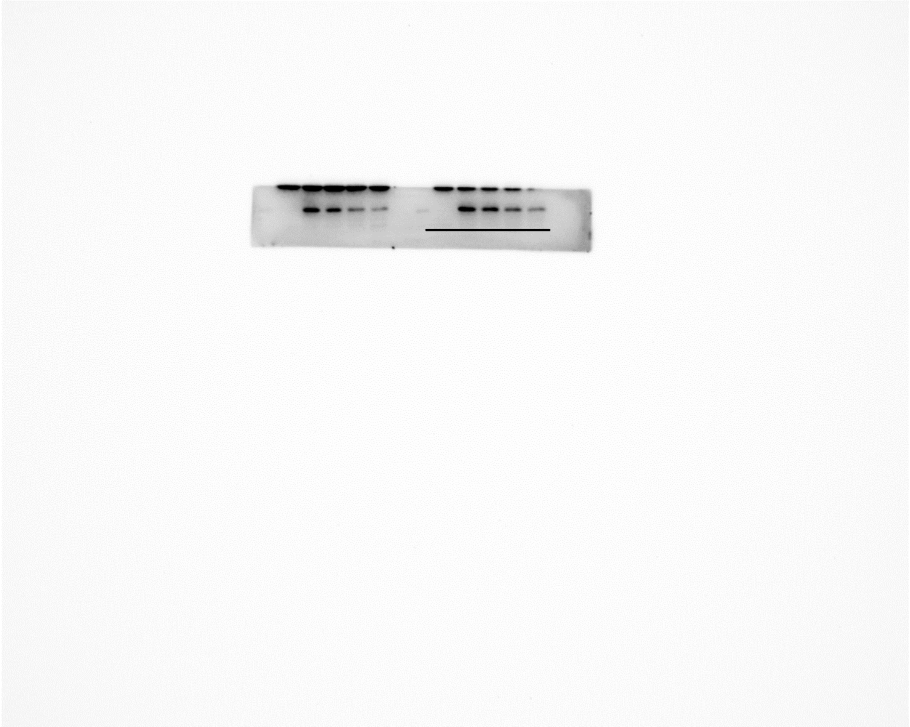

ASC	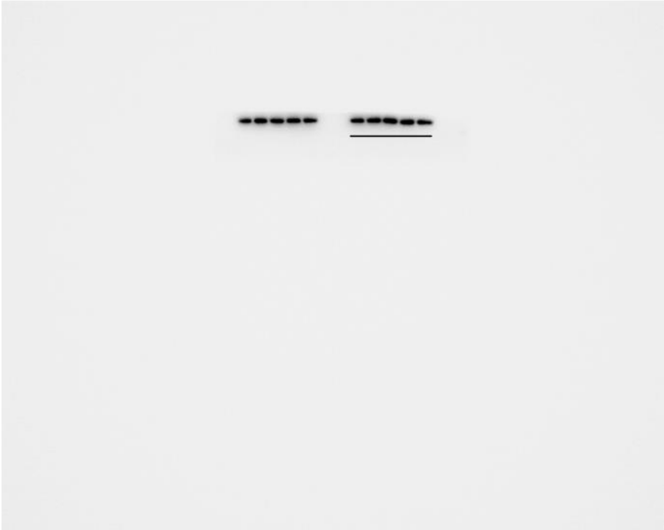
β-actin	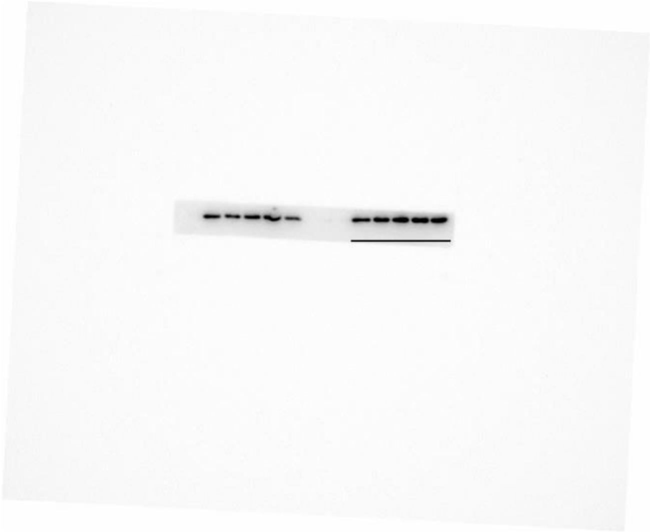

FIGURE 4E

Caspase-1 p20	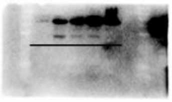

IL-1β p17	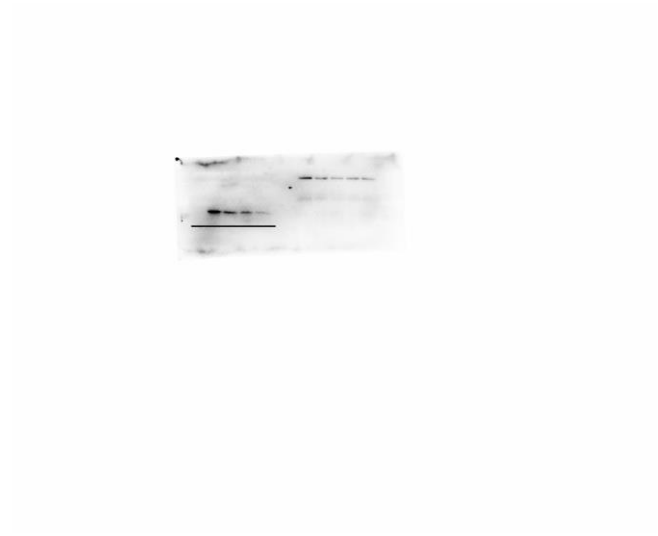
NLRP3	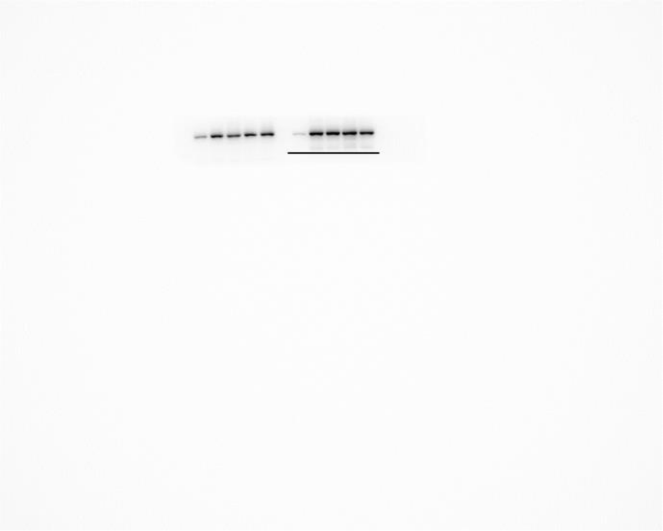
Pro-caspase-1	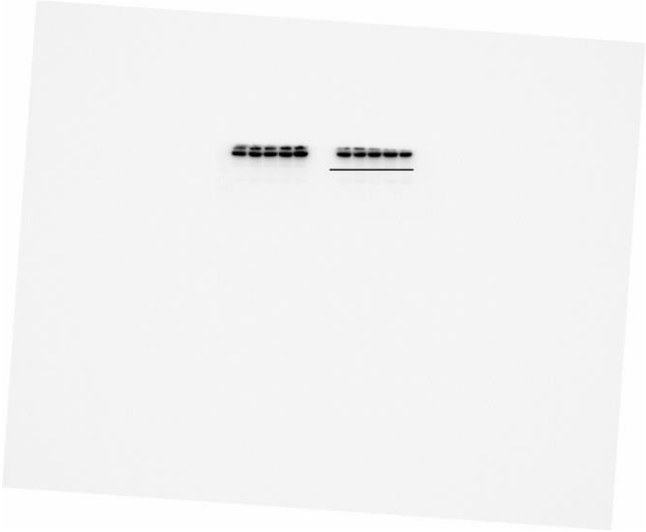

Pro-IL-1β	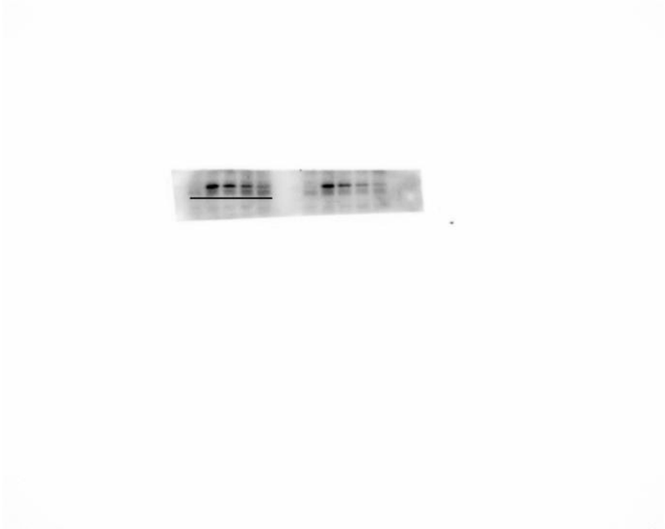
ASC	7<br>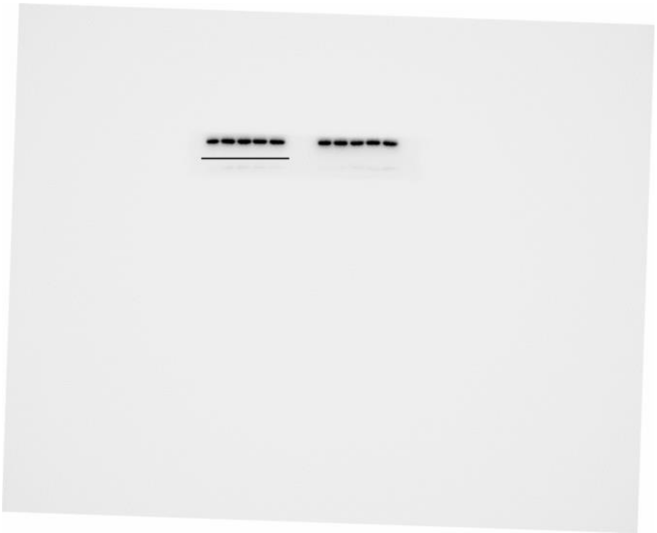
β-actin	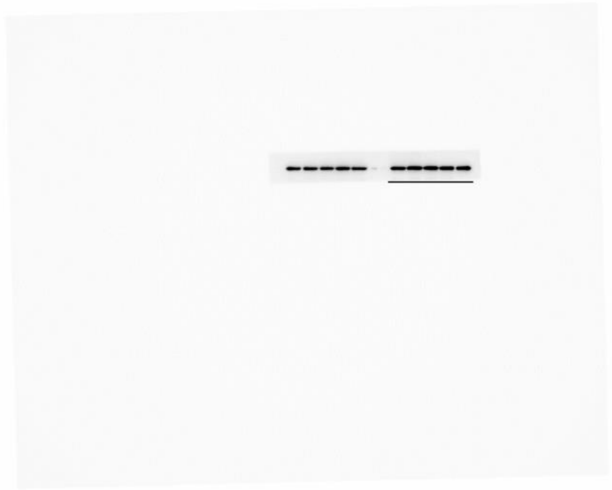

FIGURE 4G

Caspase-1 p20	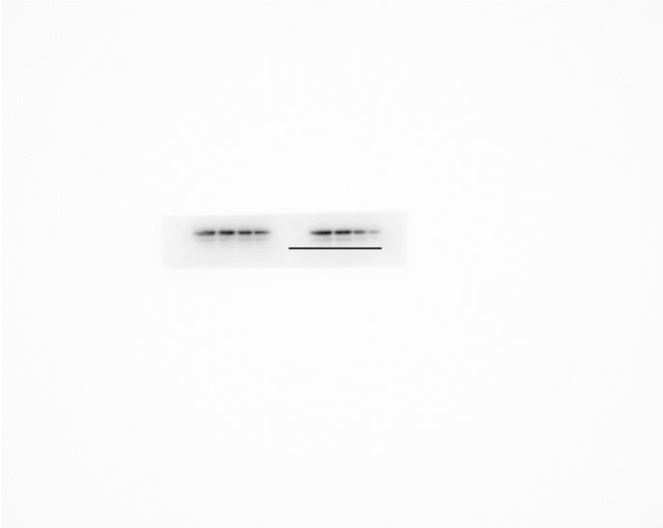
IL-1β p17	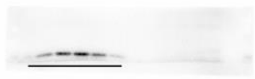
NLRP3	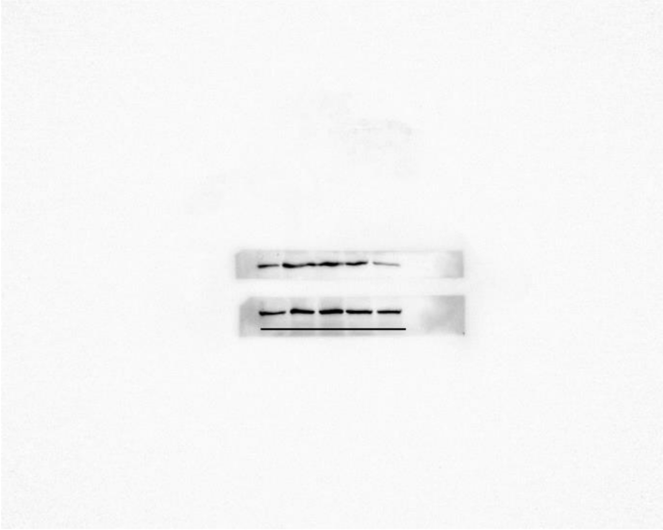

Pro-caspase-1	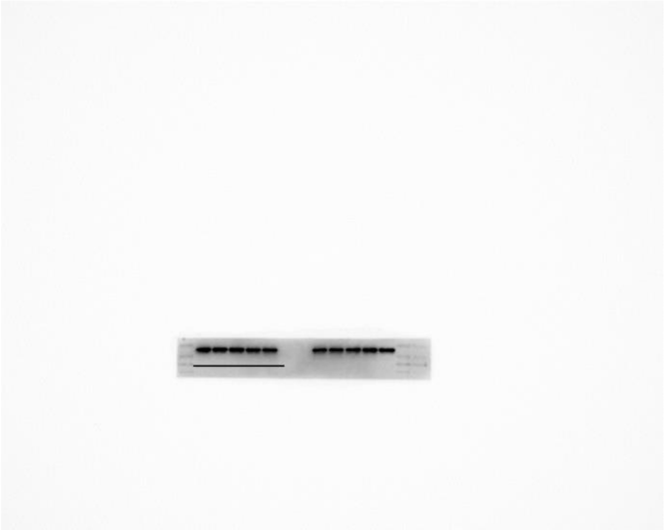
Pro-IL-1β	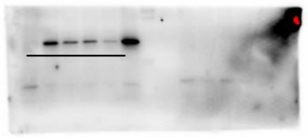
ASC	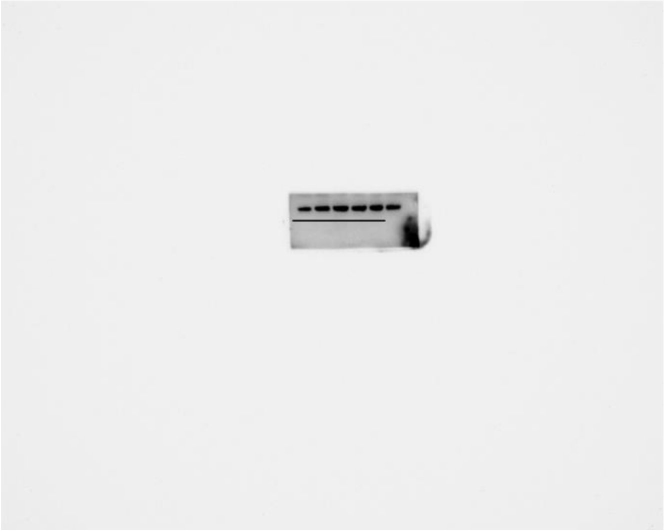

β-actin	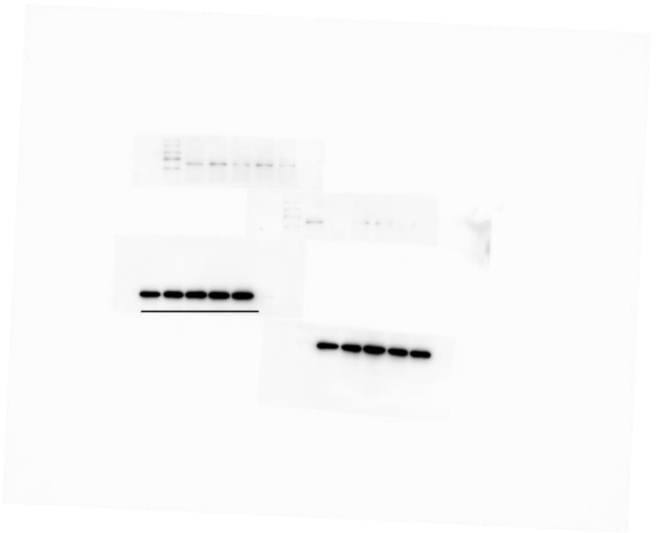

The original western blots of figure 5.

FIGURE 5A

p-p65	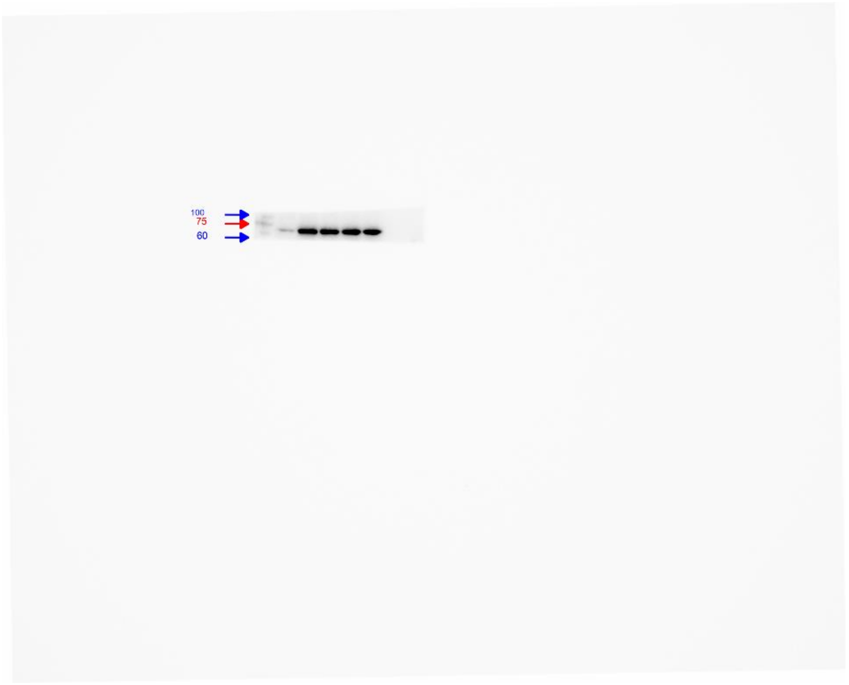

P65	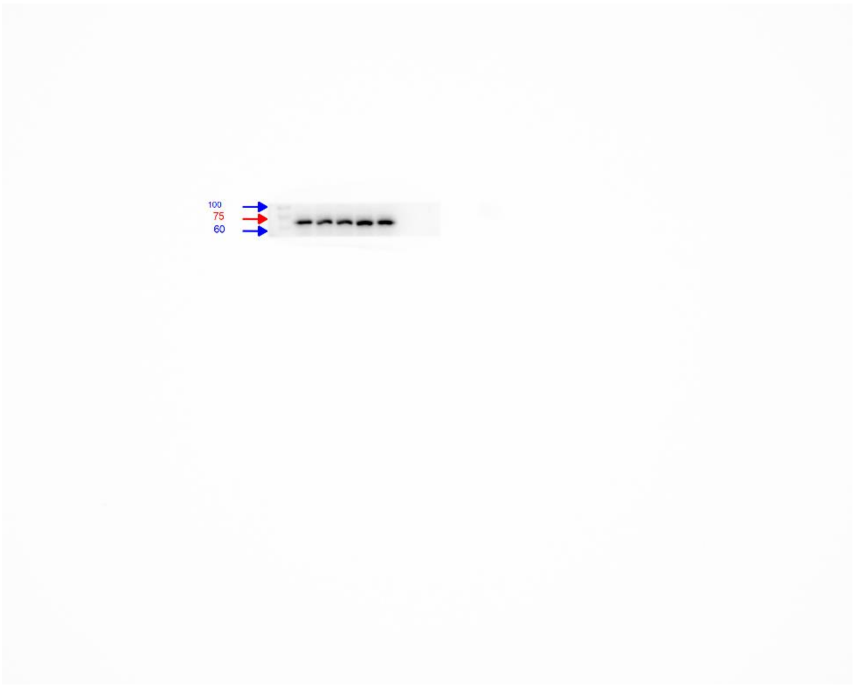
p-IκBα	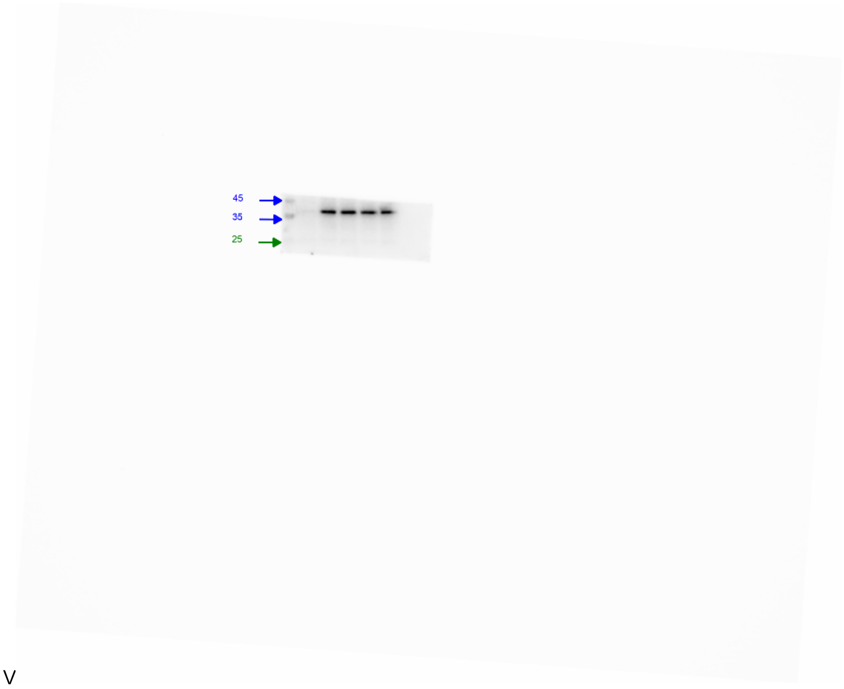<br>V

IκBα	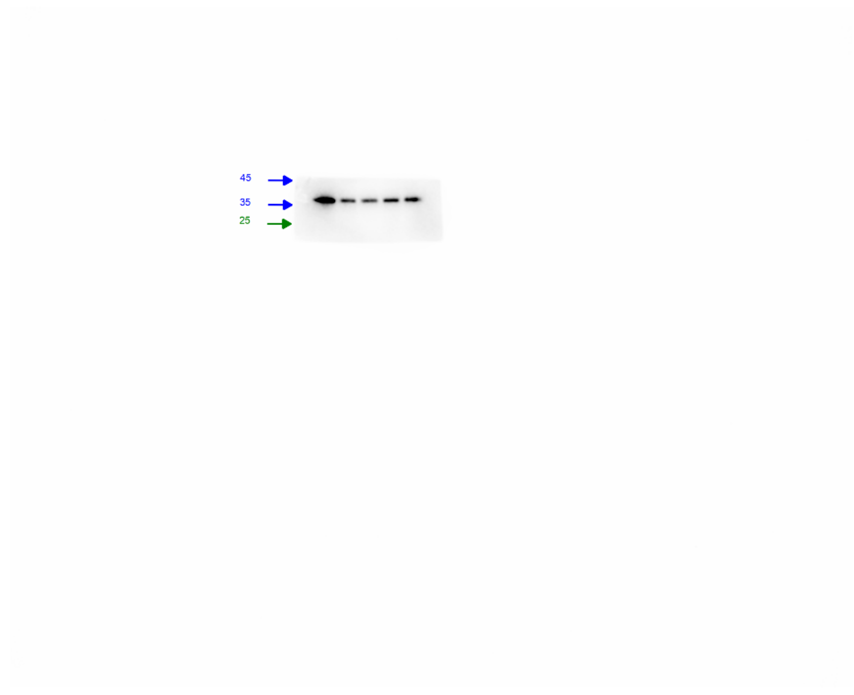
β-actin	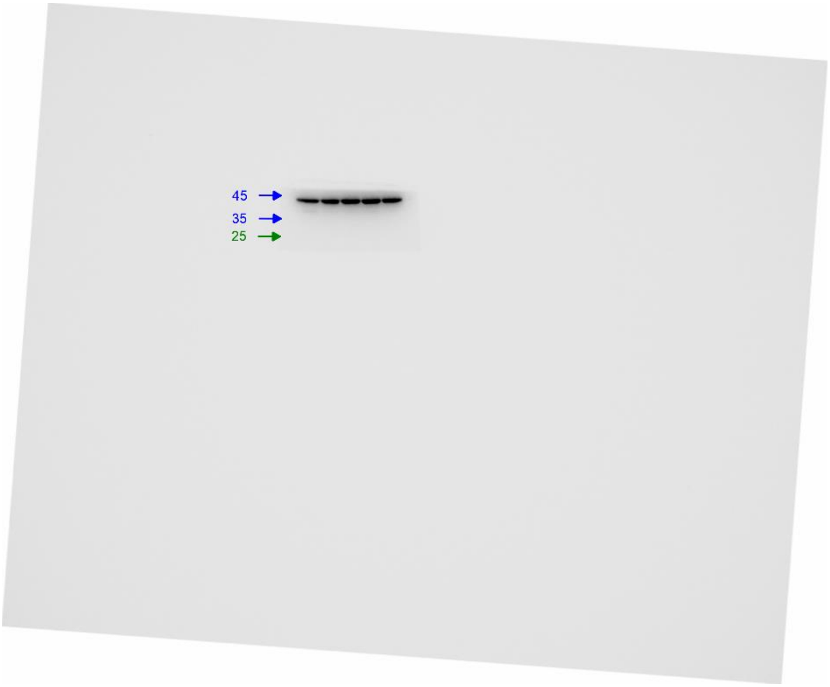

FIGURE 5C

P65	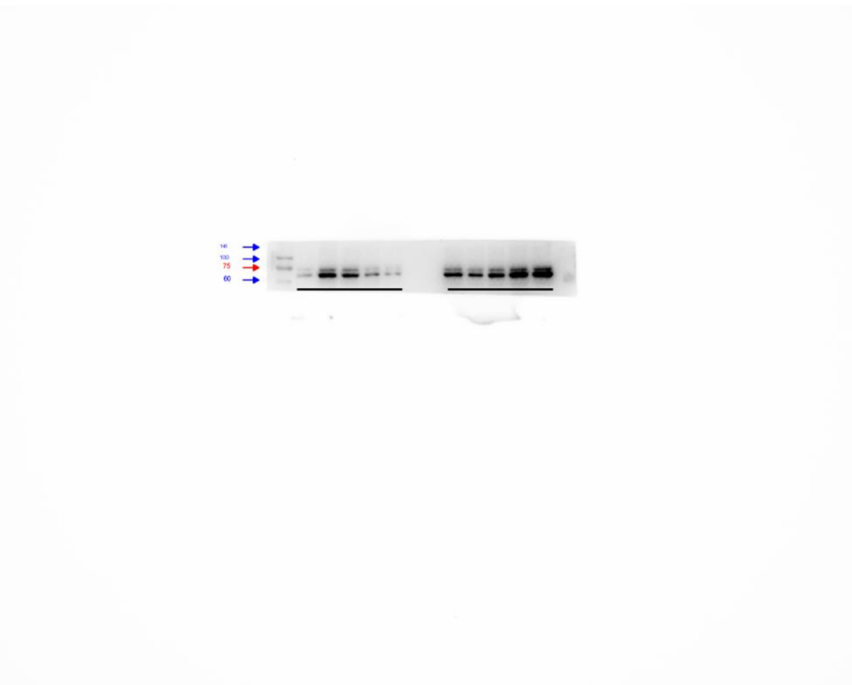
Histone H3	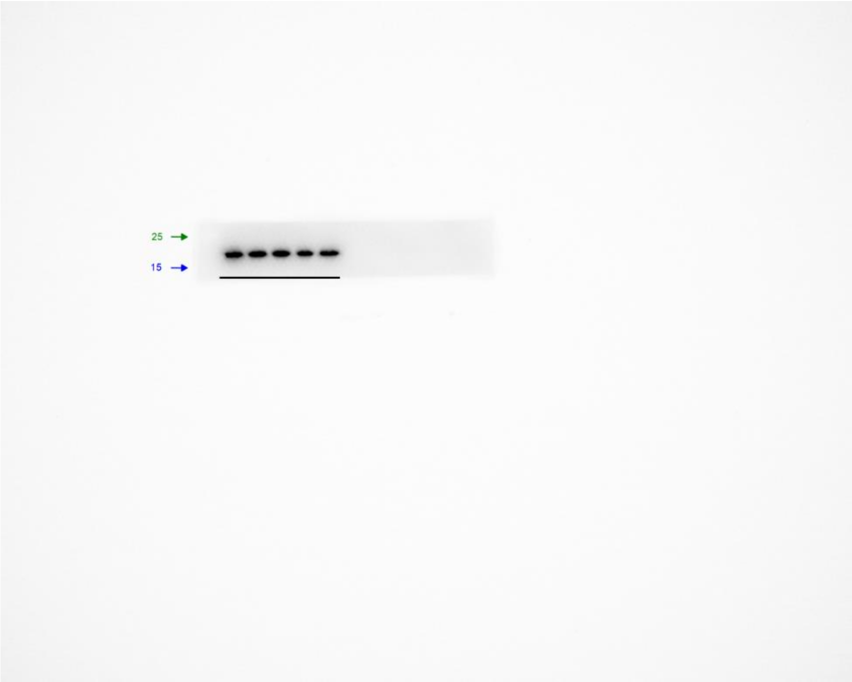

β-actin	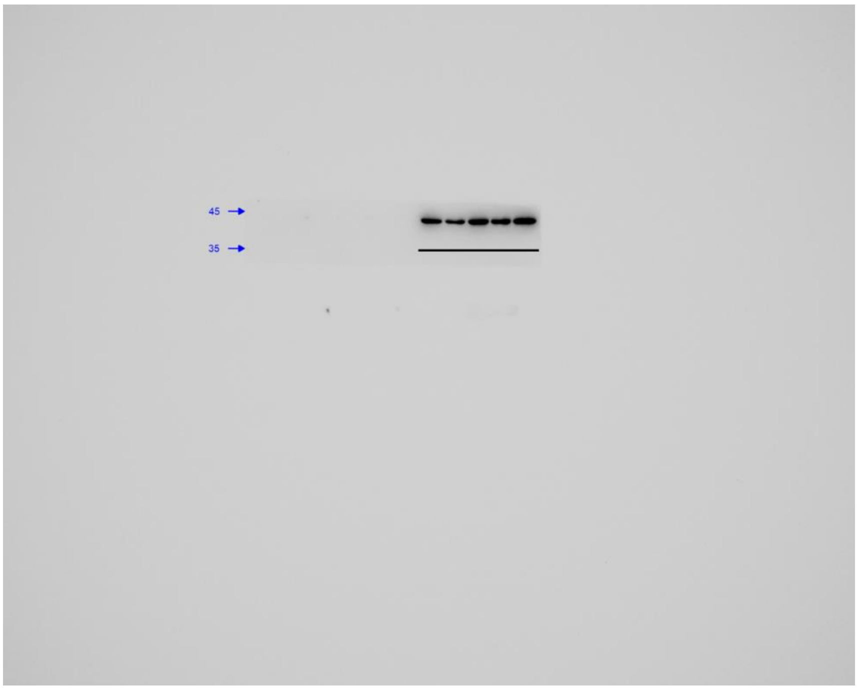

The original figures of immunofluorescent staining in figure 5.

FIGURE 5E

Vehicle<br>p65	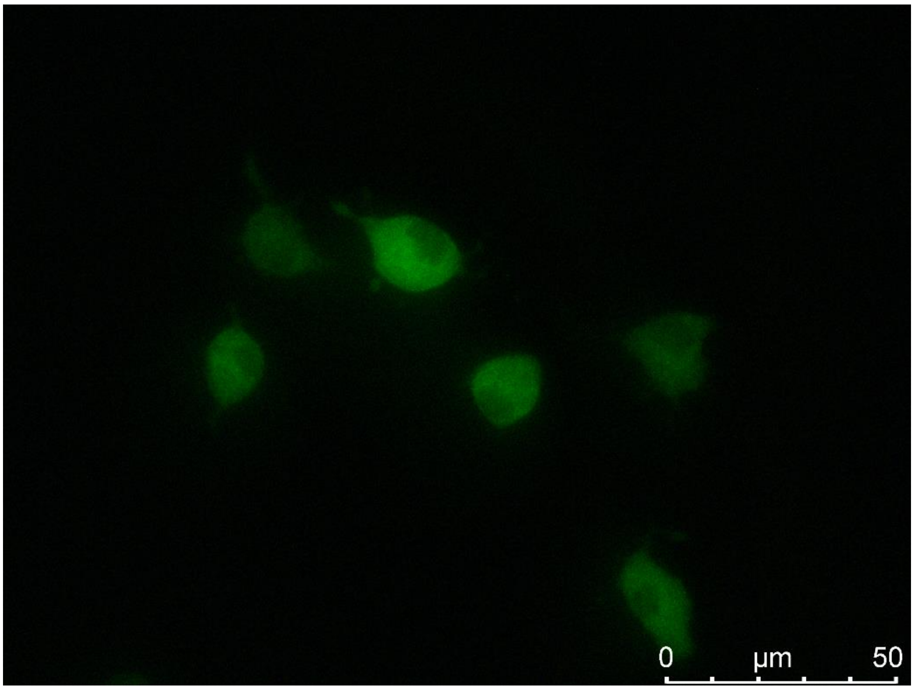

<table>
<tr><td>Vehicle
Hoechst</td><td>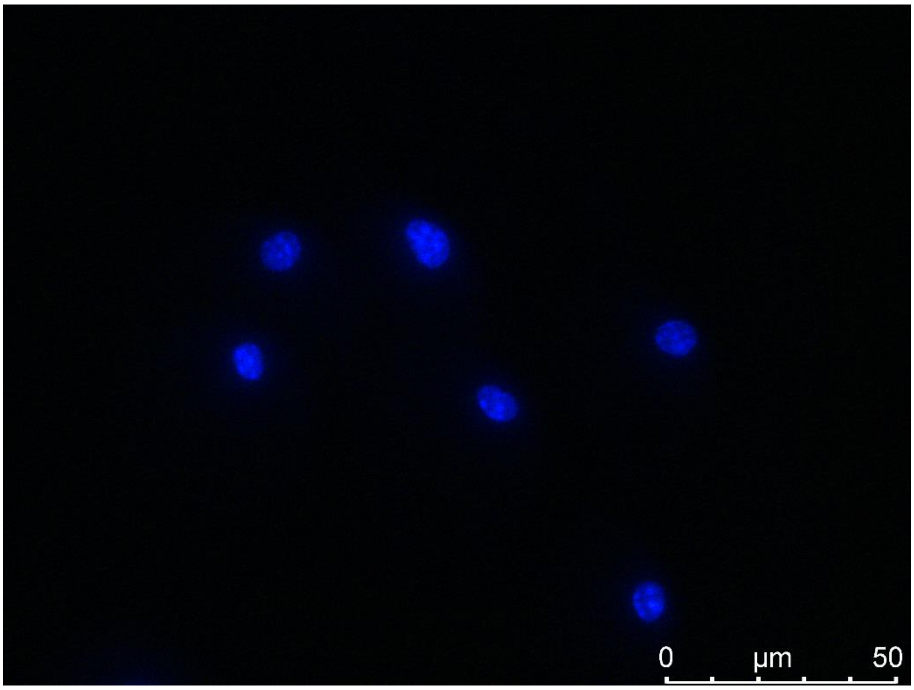
</td></tr>
<tr><td>Vehicle
Merge</td><td>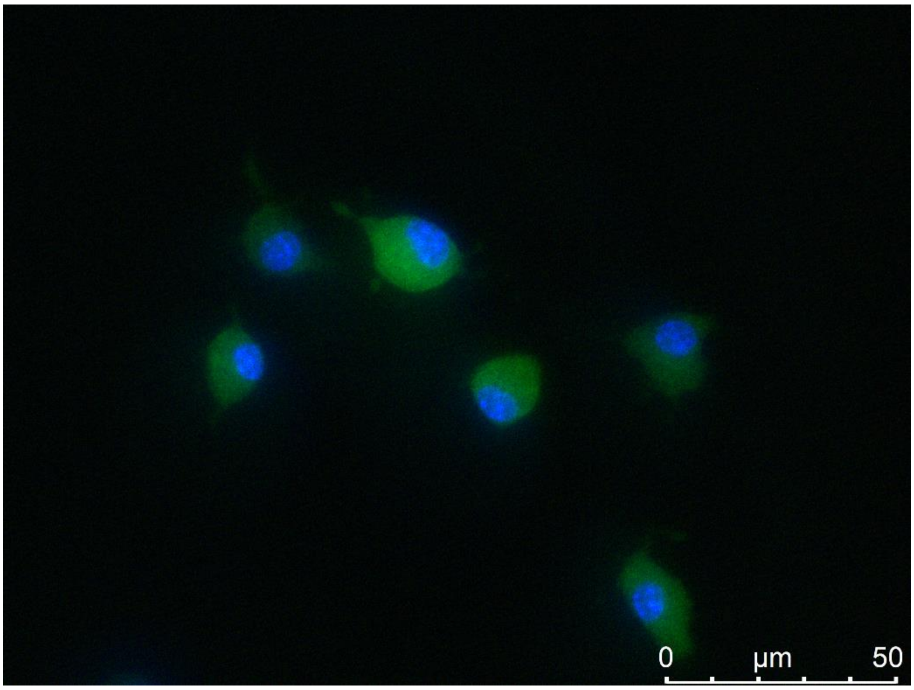
</td></tr>
</table>

LPS P65	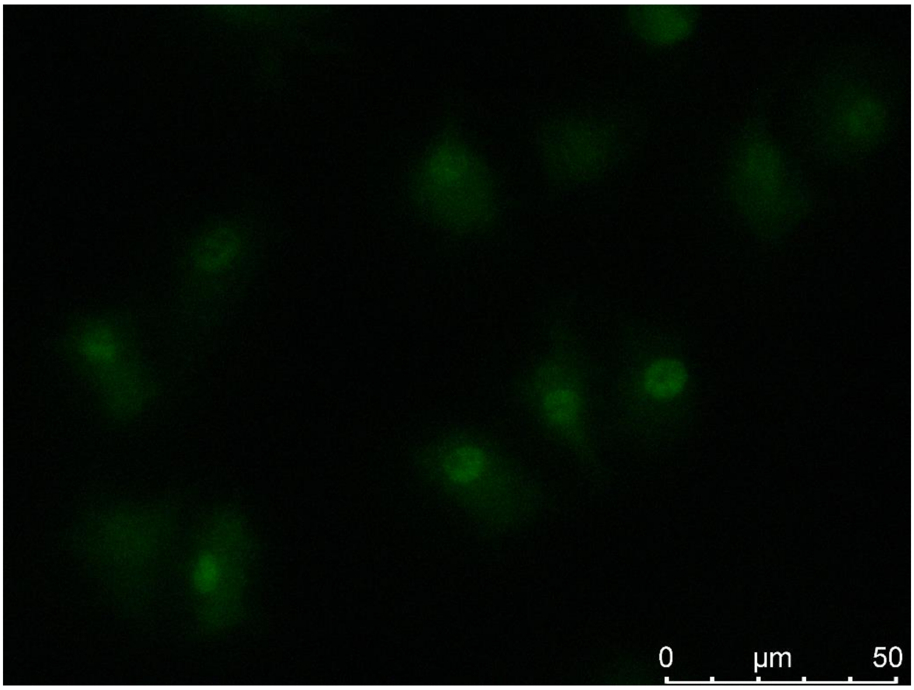
LPS Hoechst	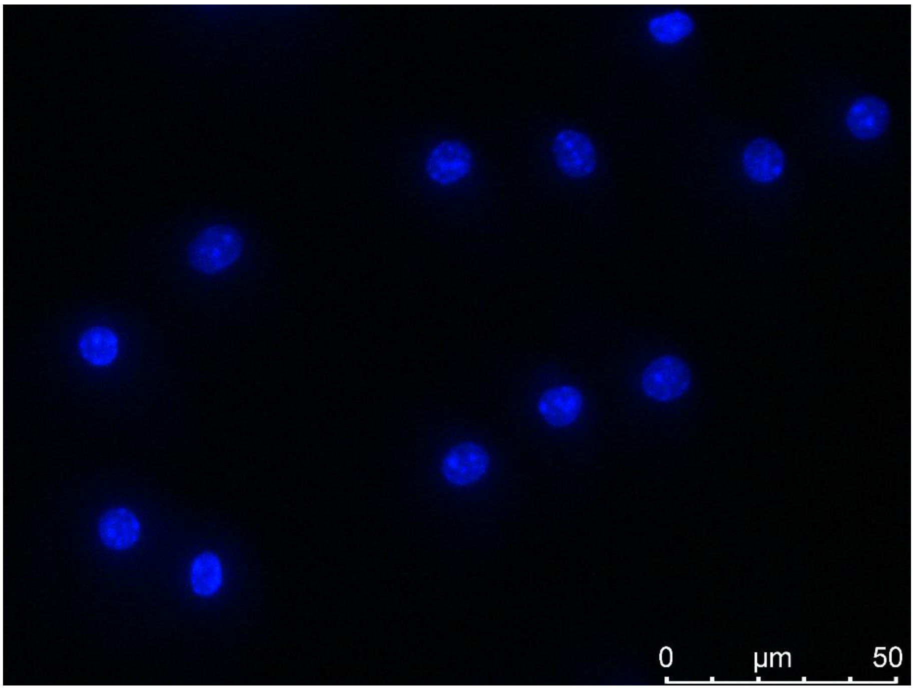

LPS<br>Merge	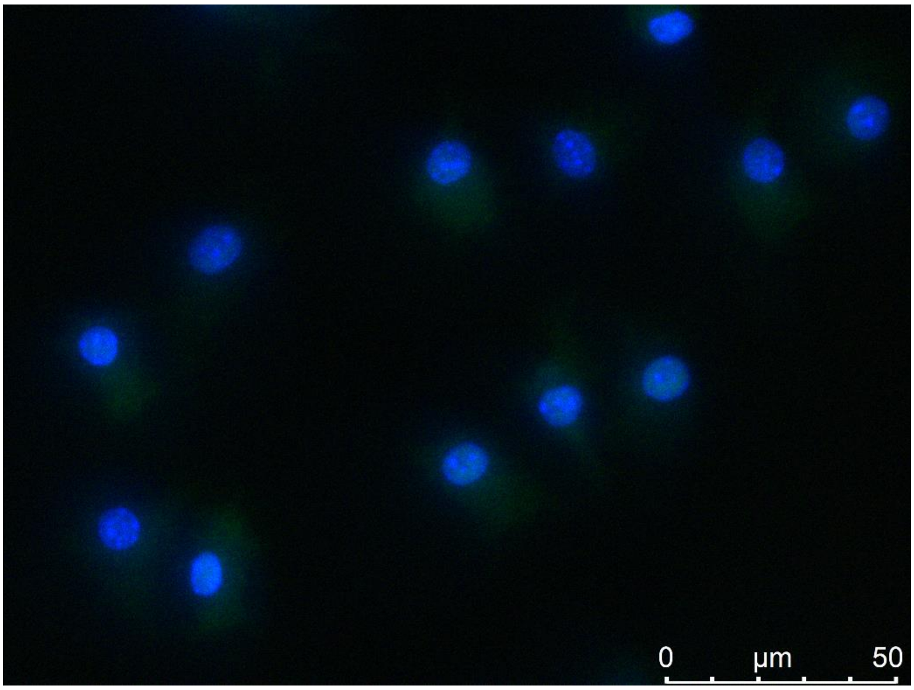
KM+LPS<br>p65	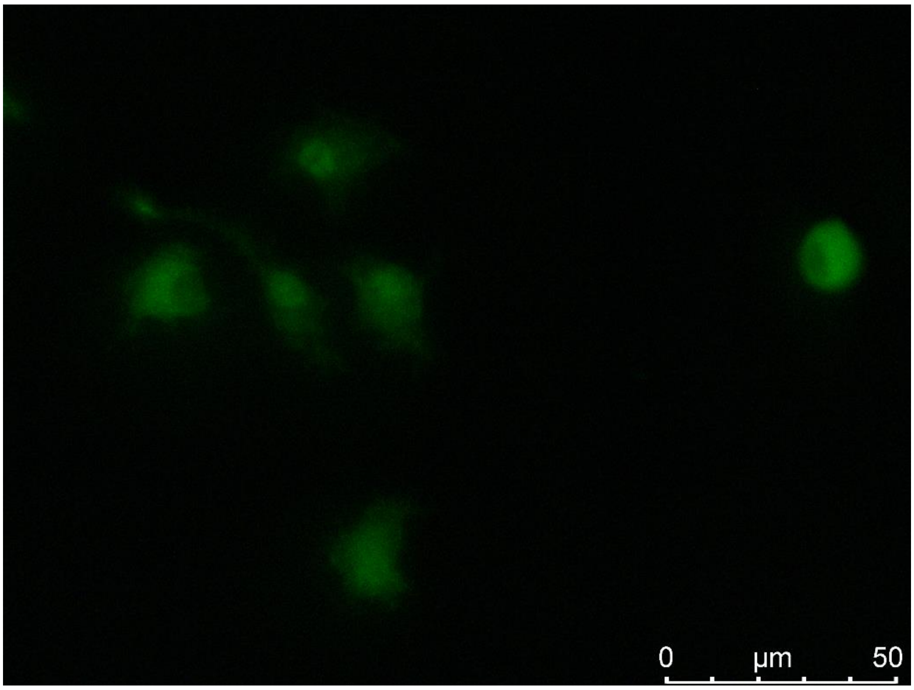

KM+LPS Hoechst	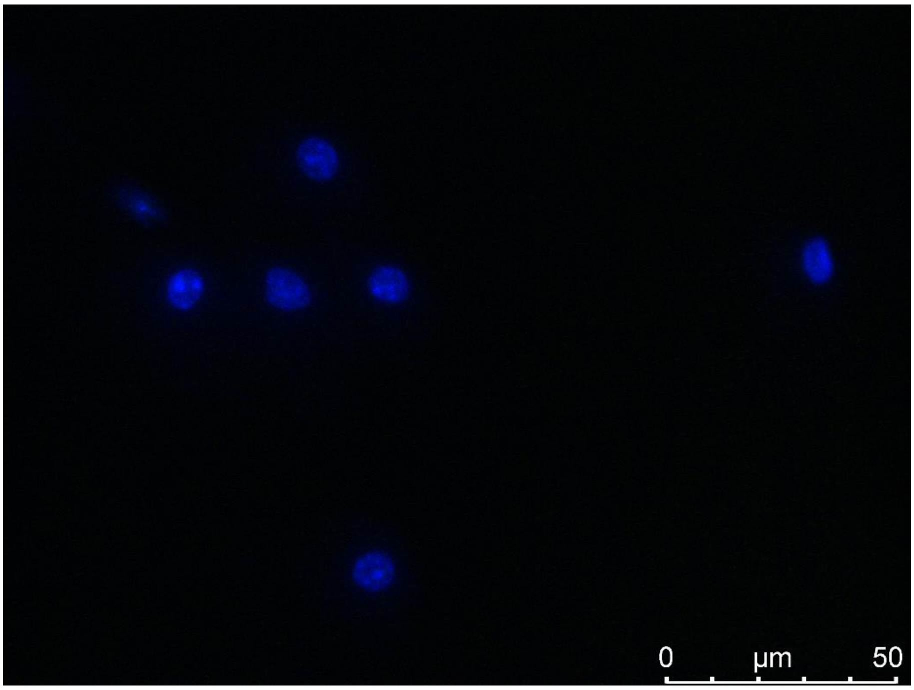
KM+LPS Merge	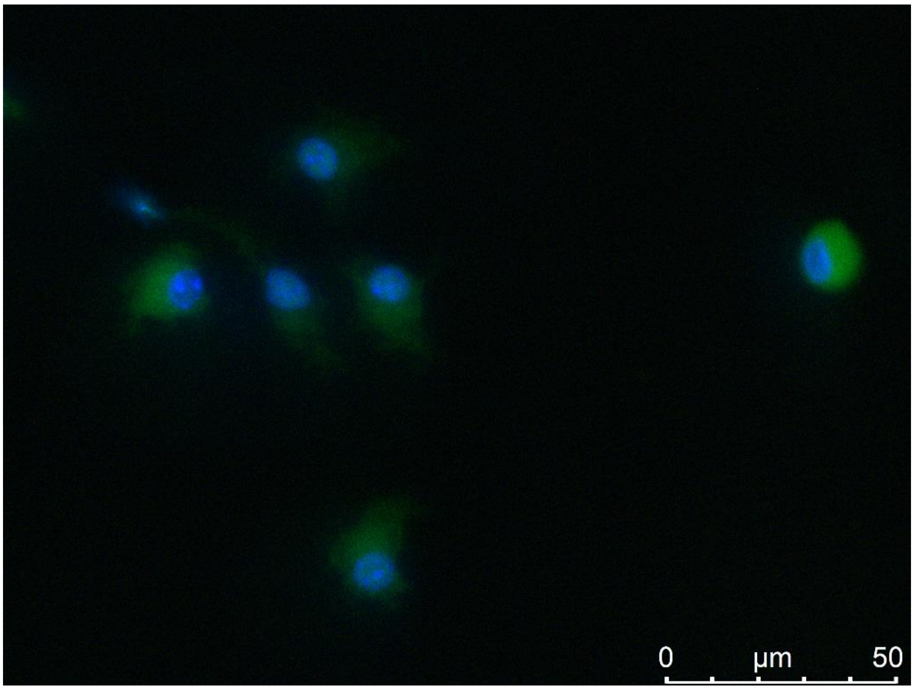

The original figures of immunofluorescent staining in figure 6.

FIGURE 6A

Vehicle ASC	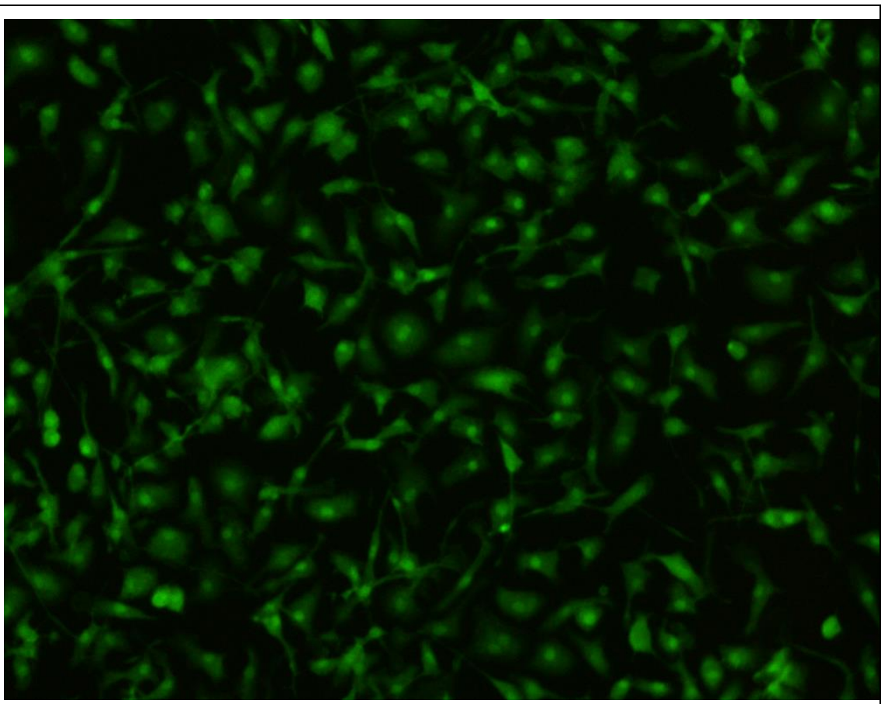
Vehicle Hoechst	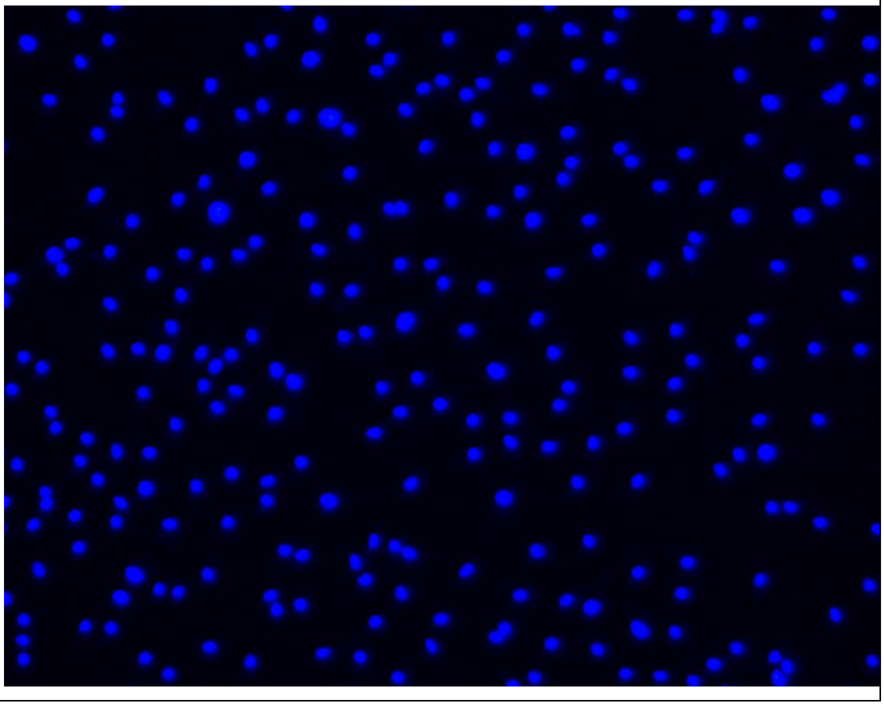

Vehicle Merge	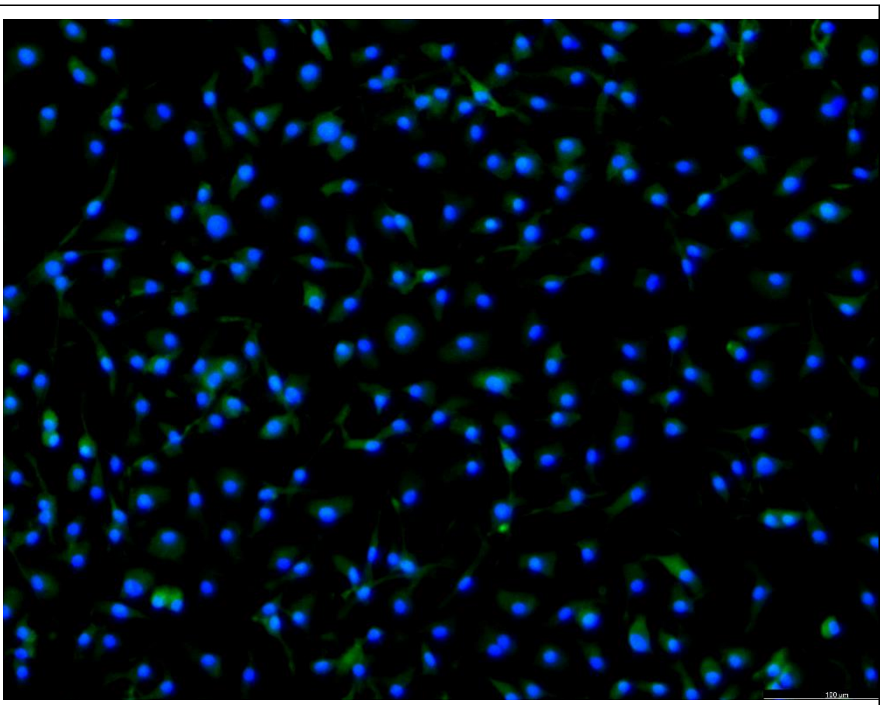
LPS+ATP<br>ASC	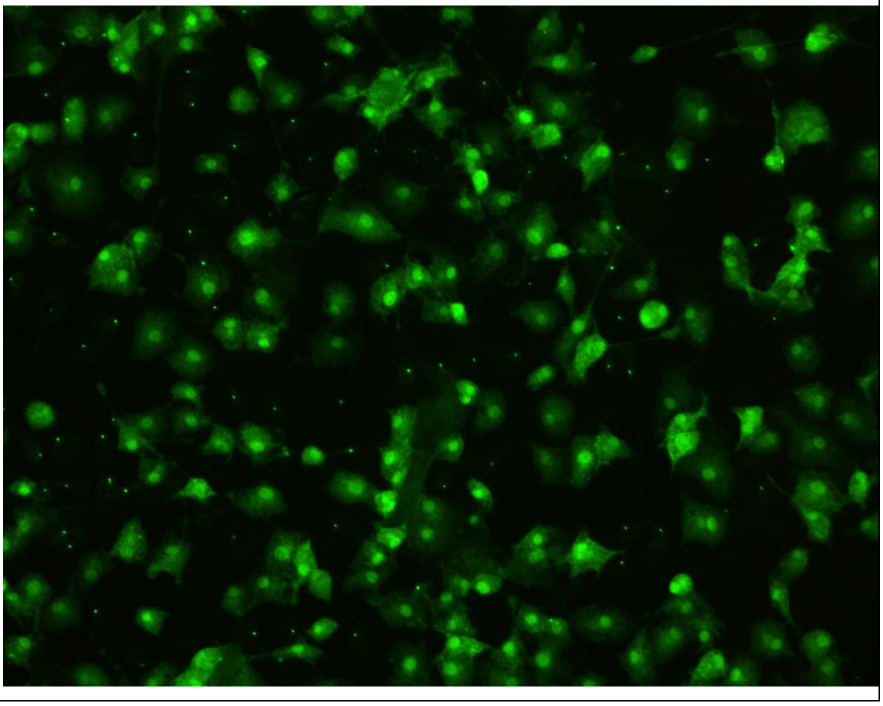

LPS+ATP<br>Hoechst	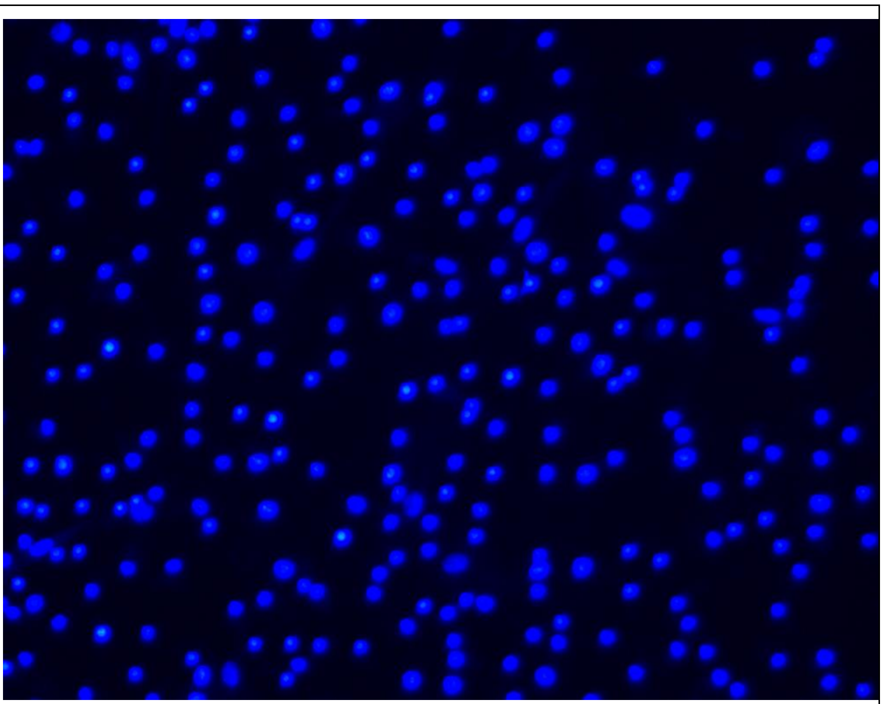
LPS+ATP<br>Merge	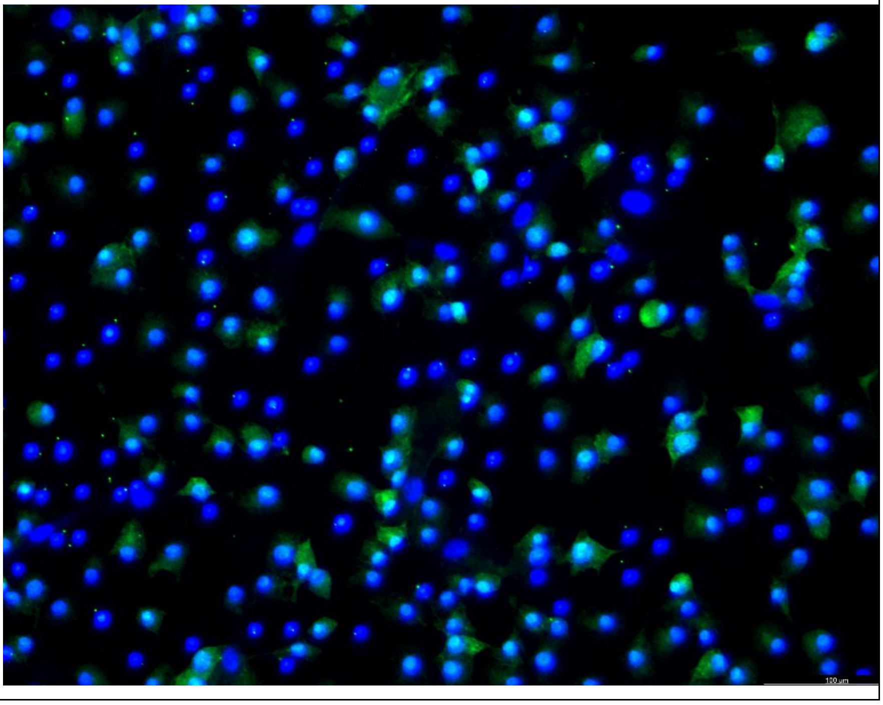

KM+LPS+ATP ASC	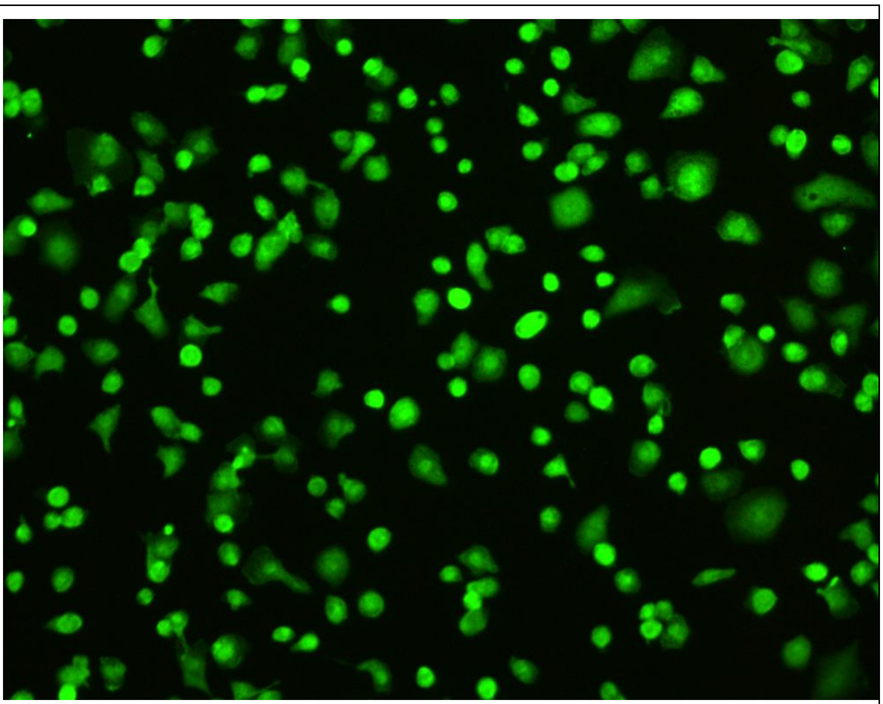
KM+LPS+ATP Hoechst	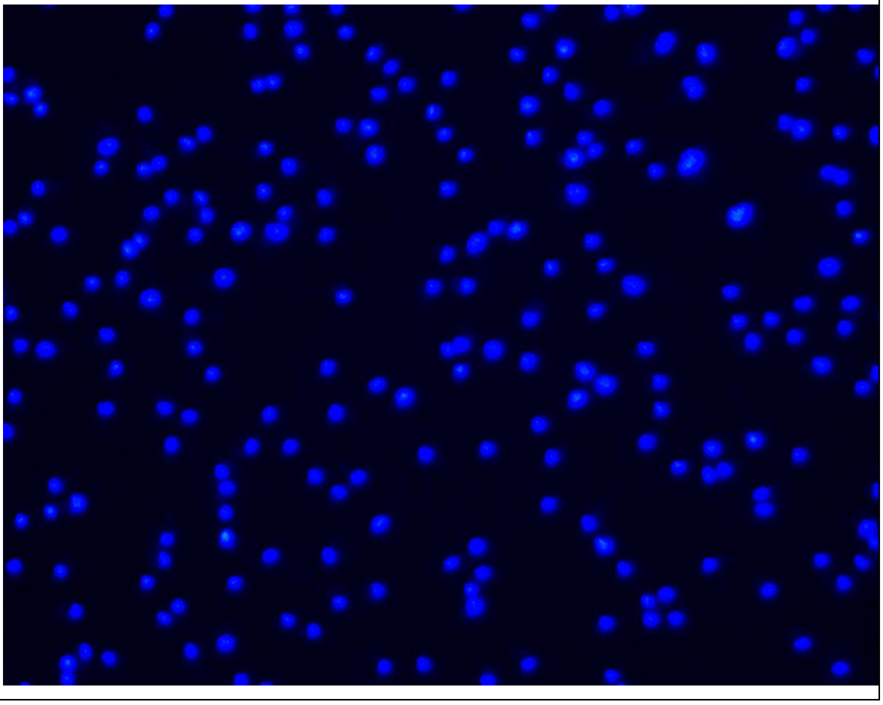

KM+LPS+ATP Merge	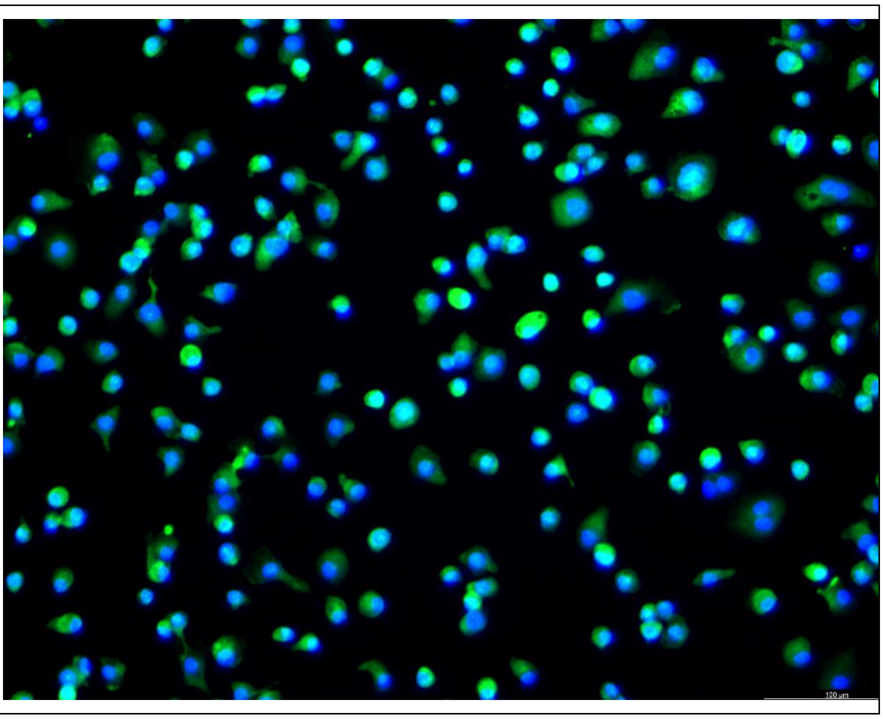

FIGURE 6D

Vehicle NLRP3	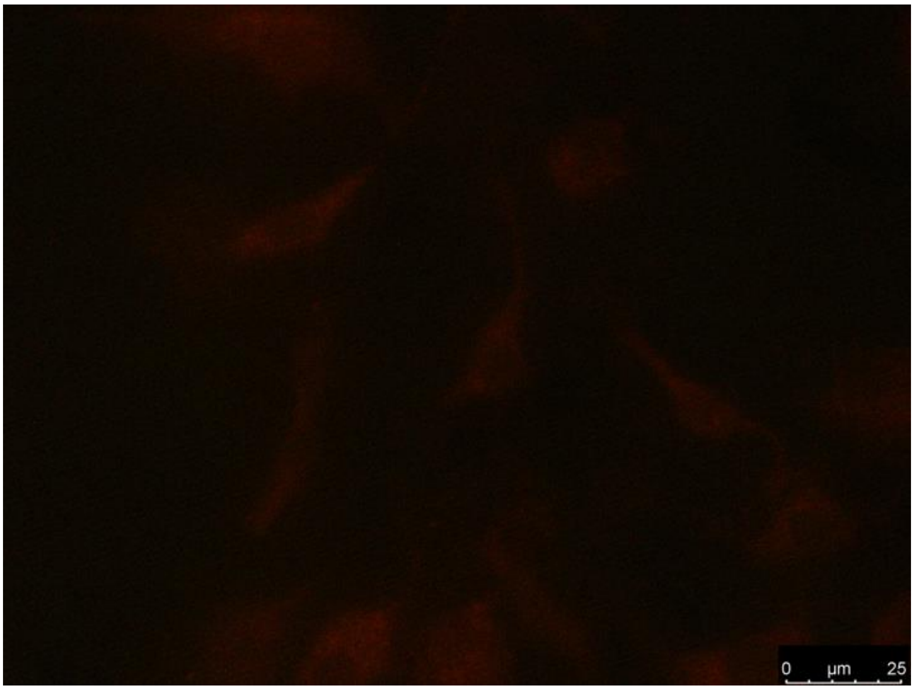

Vehicle<br>ASC	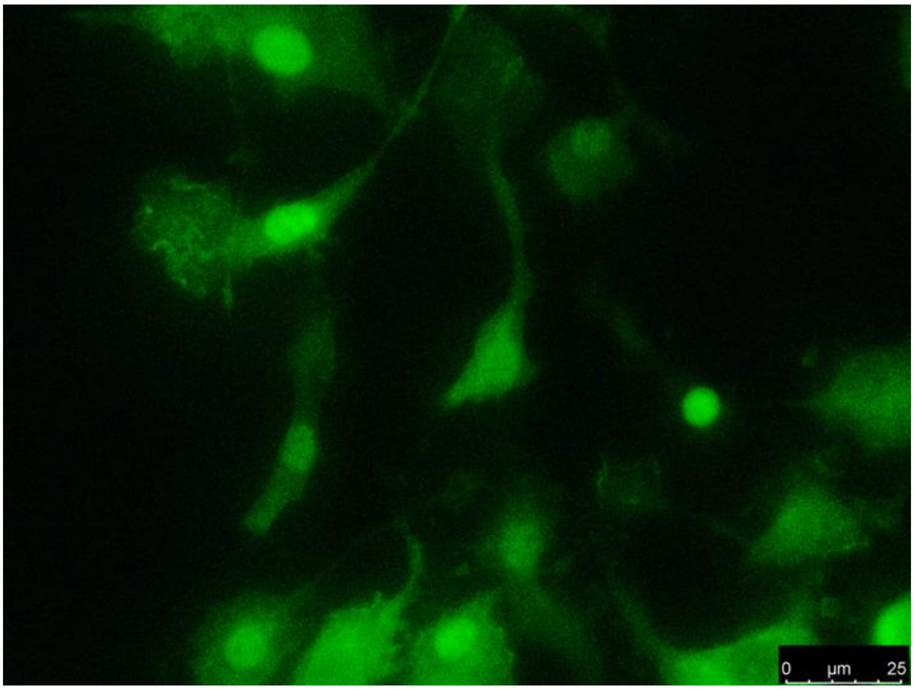
Vehicle<br>Hoechst	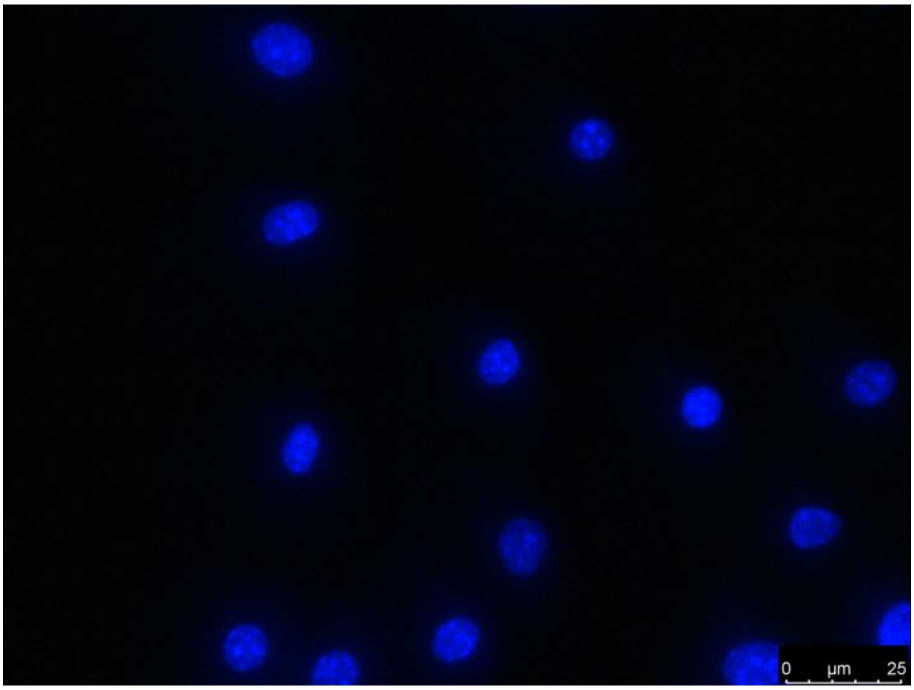

Vehicle<br>Merge	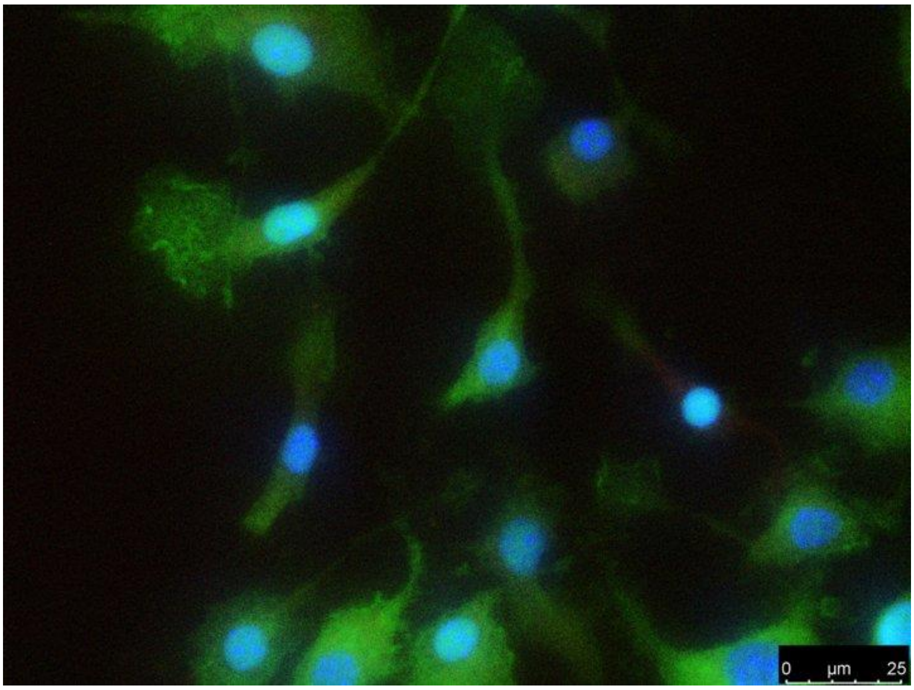
LPS+ATP<br>NLRP3	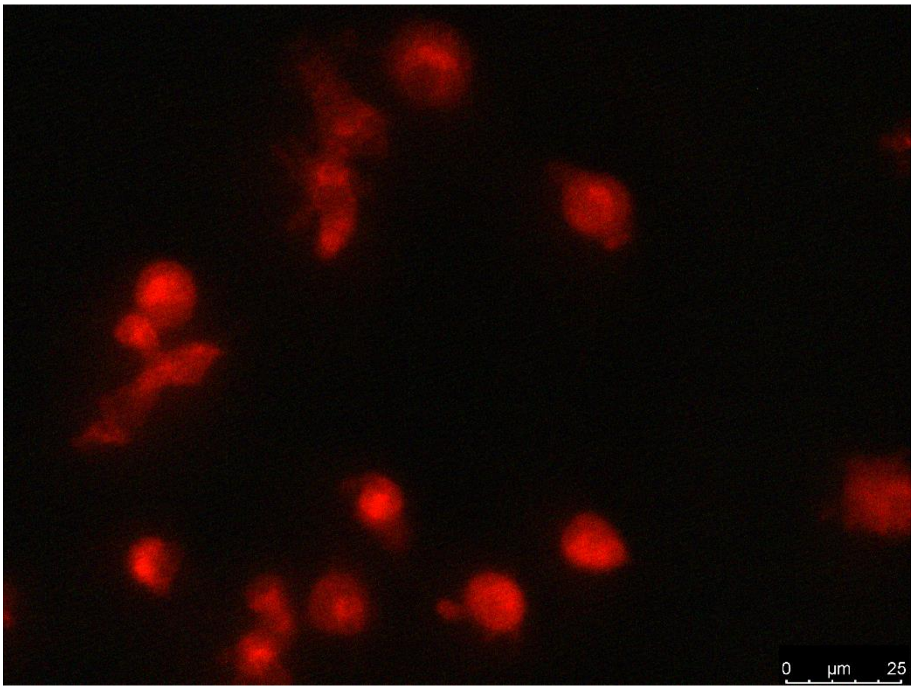

LPS+ATP<br>ASC	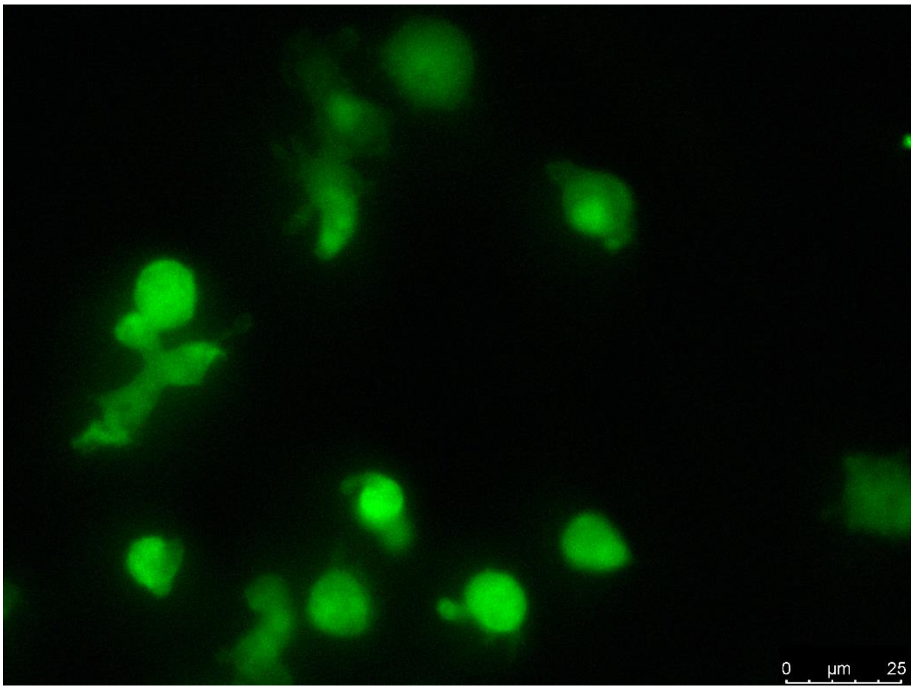
LPS+ATP<br>Hoechst	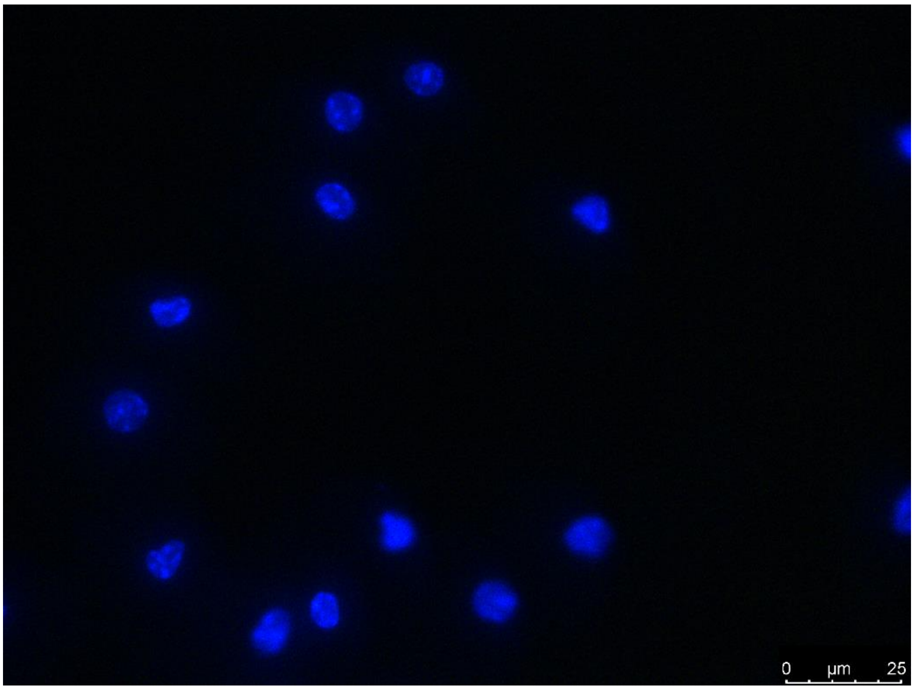

LPS+ATP<br>Merge	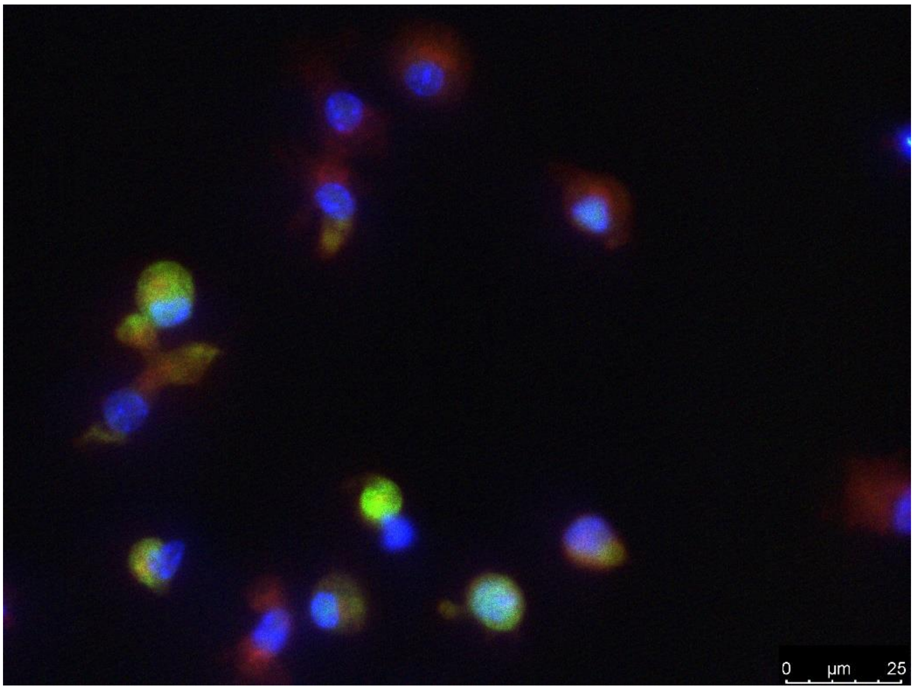
KM+<br>LPS+ATP<br>NLRP3	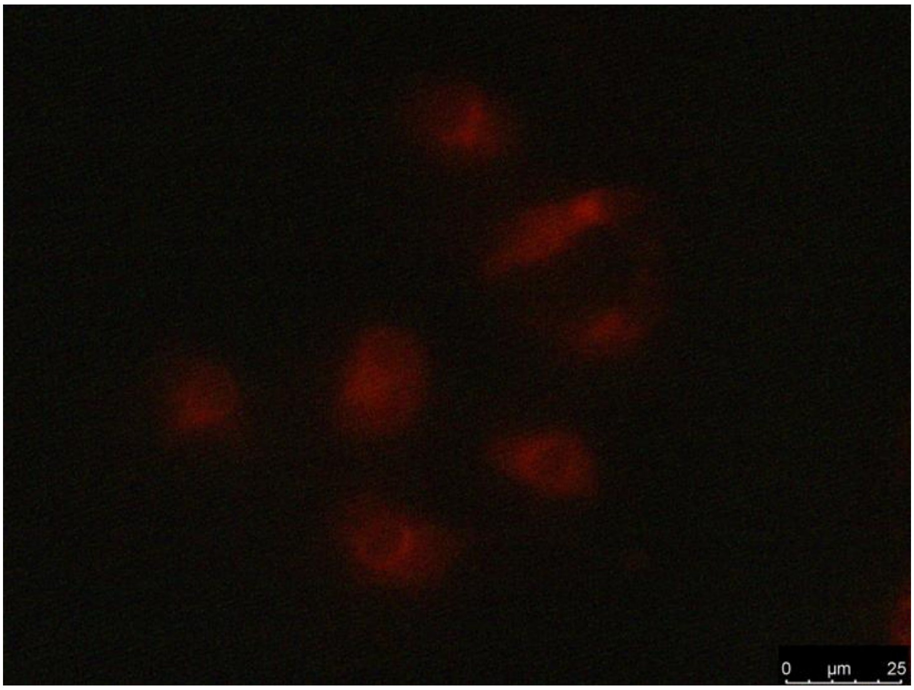

KM+<br>LPS+ATP<br>ASC	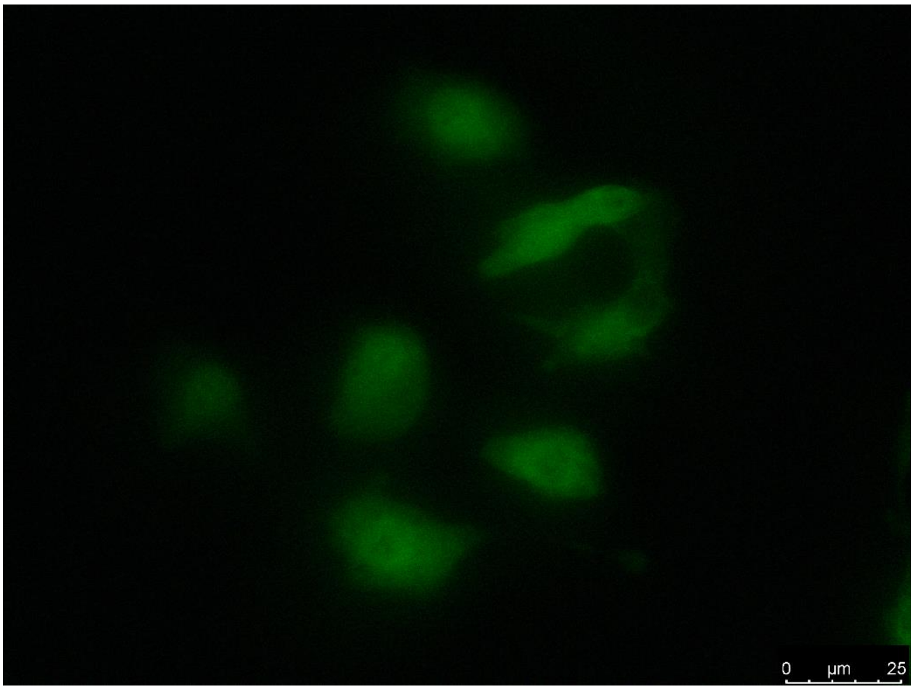
KM+<br>LPS+ATP<br>Hoechst	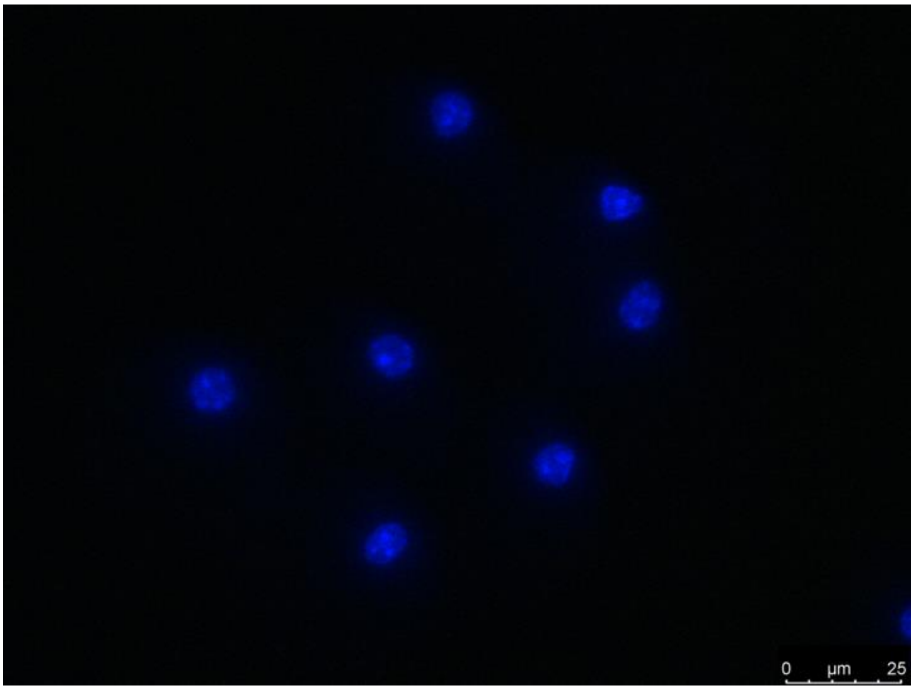

KM+ LPS+ATP Merge	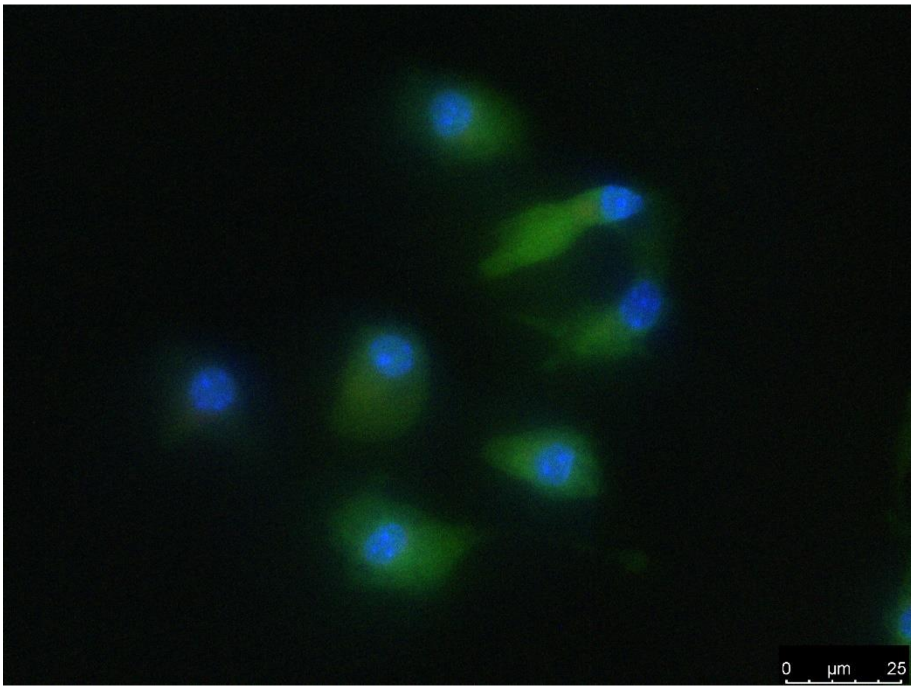

The original western blots of figure 6.

FIGURE 6C

ASC oligomerization	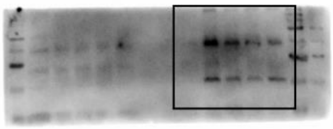

ASC	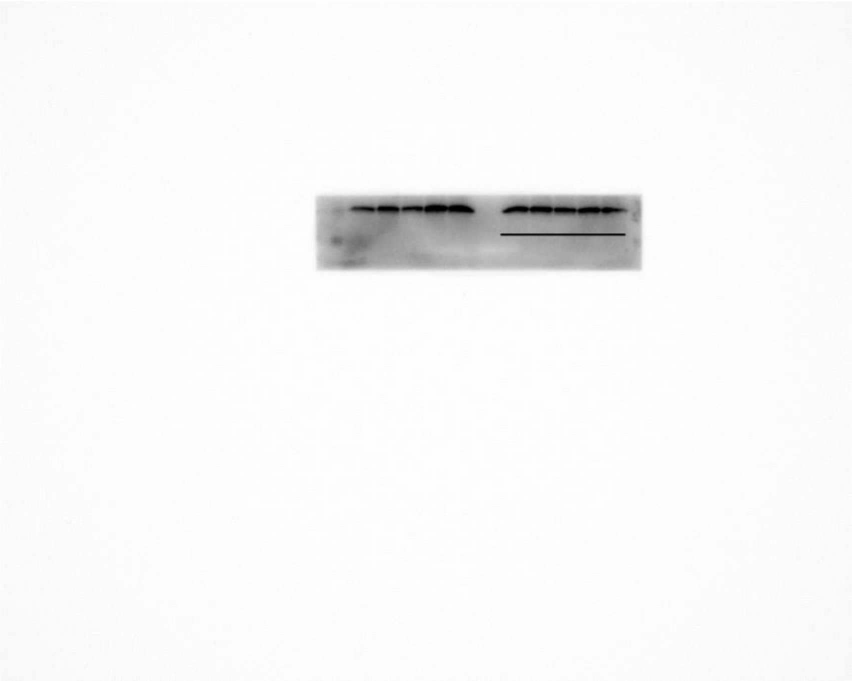
β-actin	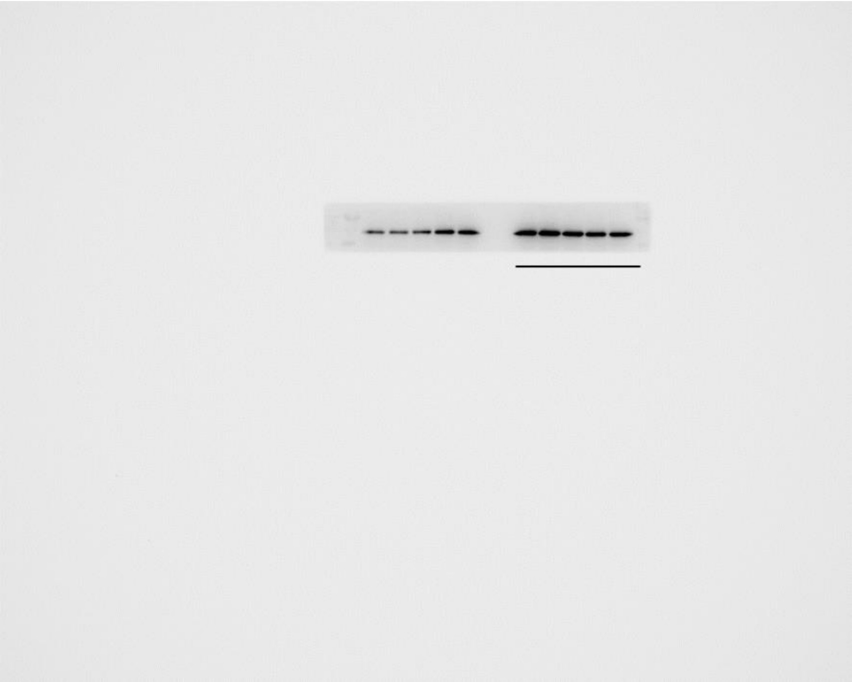

FIGURE 6E

IP-NLRP3	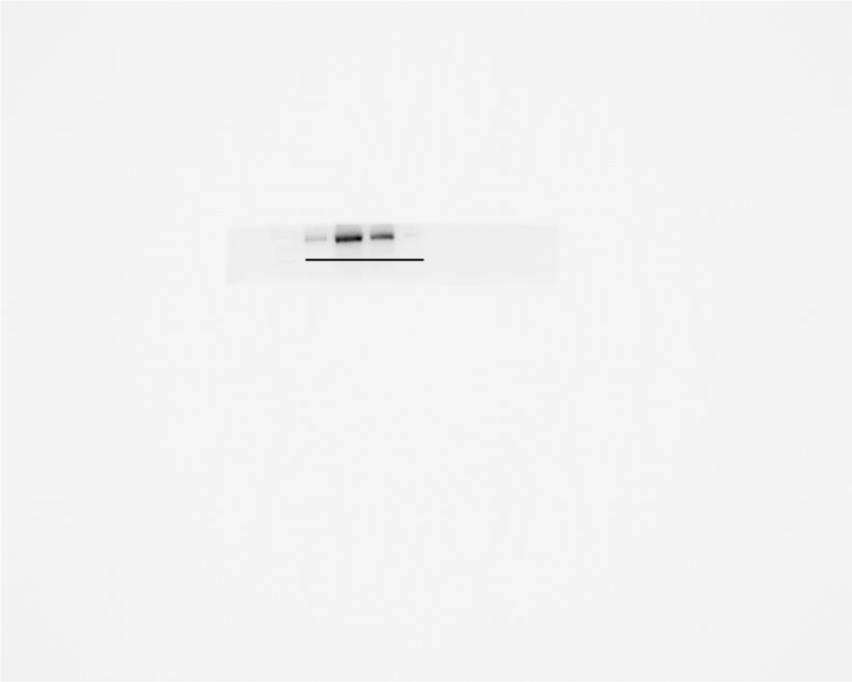
IP-pro-caspase-1	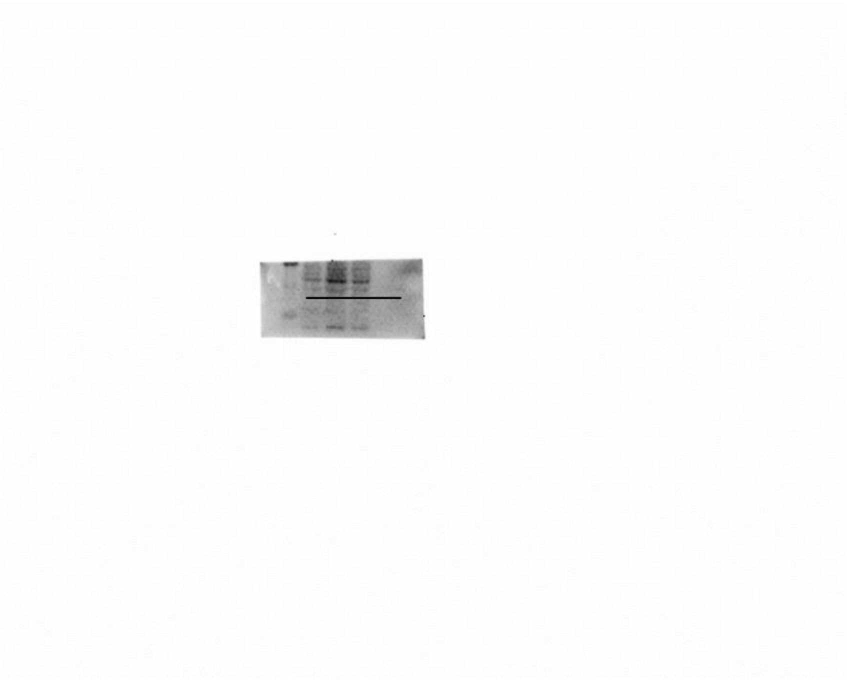

IP-ASC	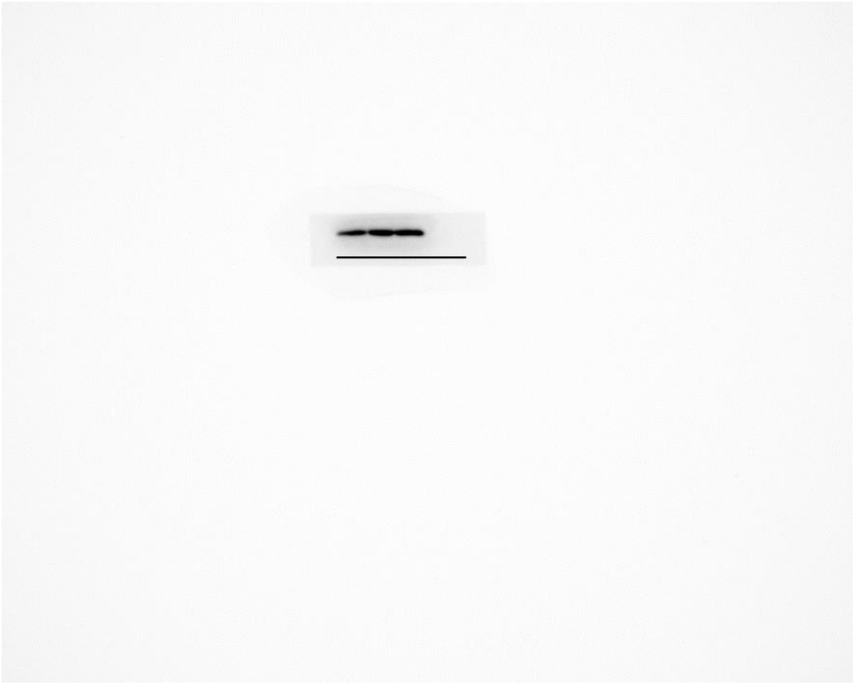
Input-NLRP3	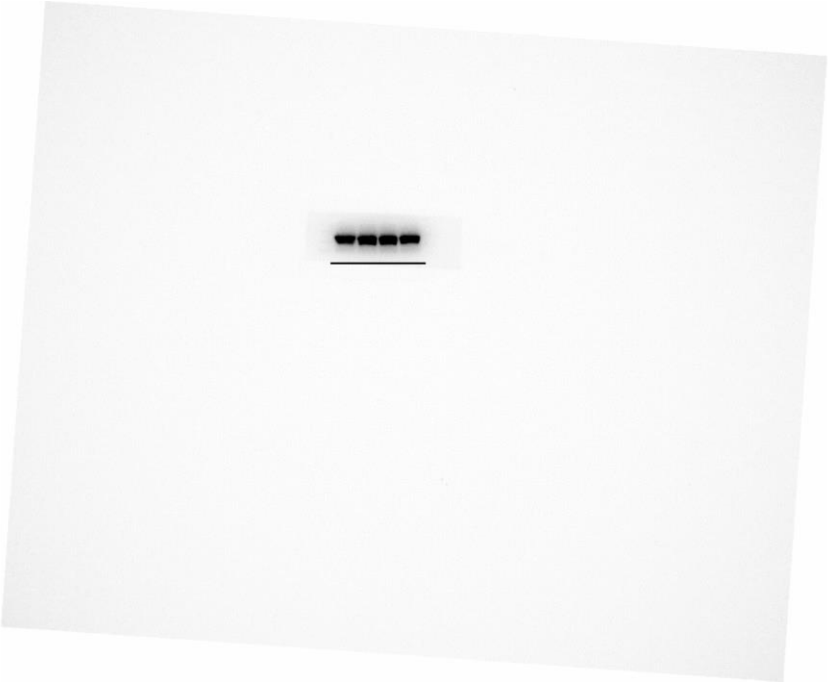

Input-pro-caspase-1	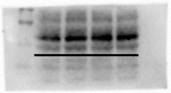
Input-ASC	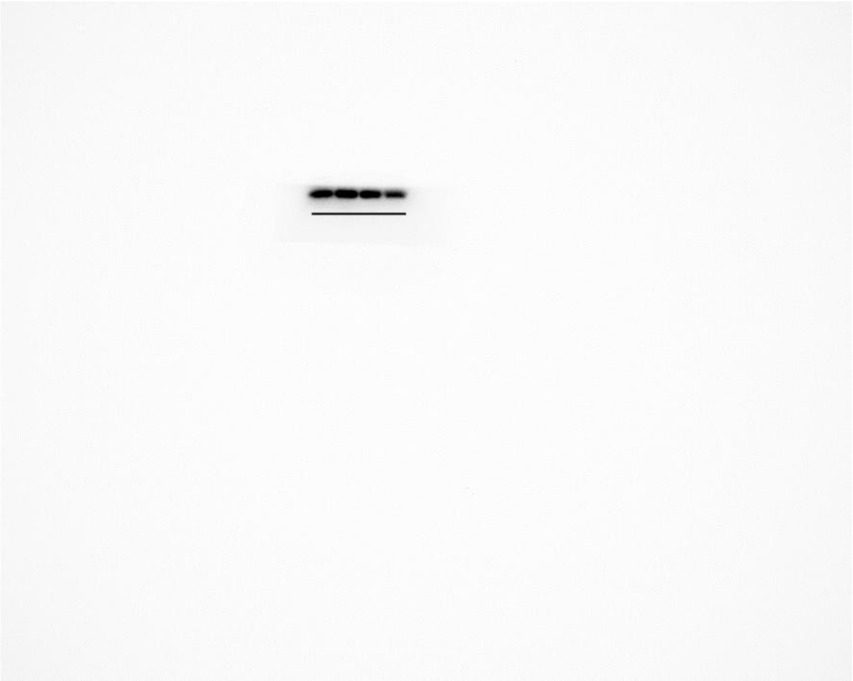

Input-β-actin	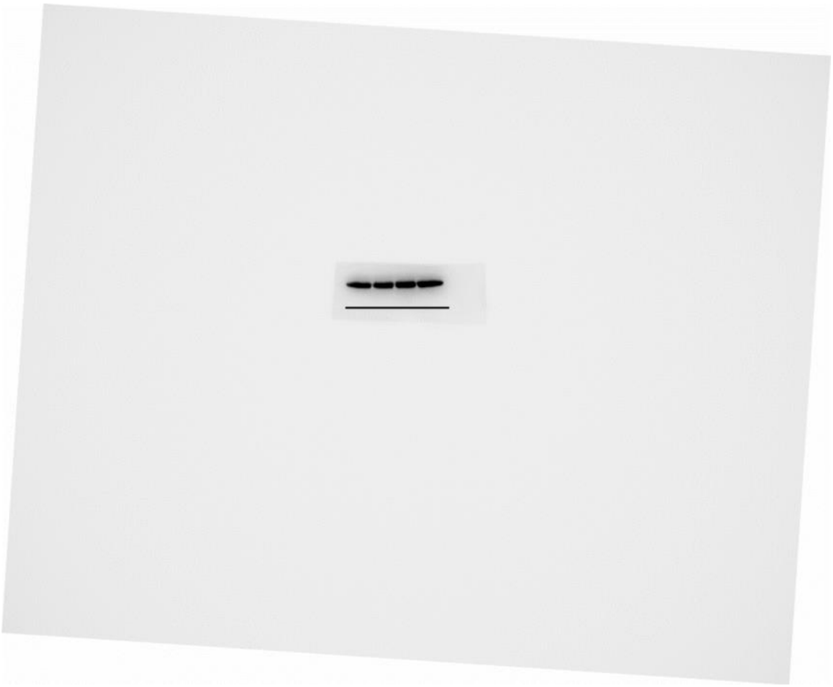

The original figures of DCFH-DA staining in figure 7.

FIGURE 7A

Vehicle-DCFH-DA	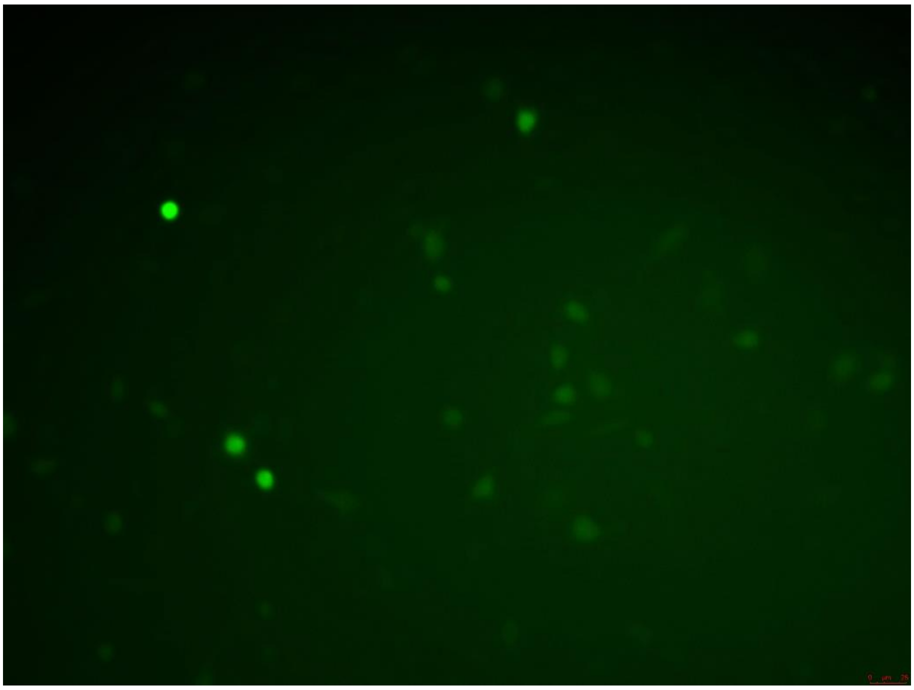

Vehicel-Hoechst	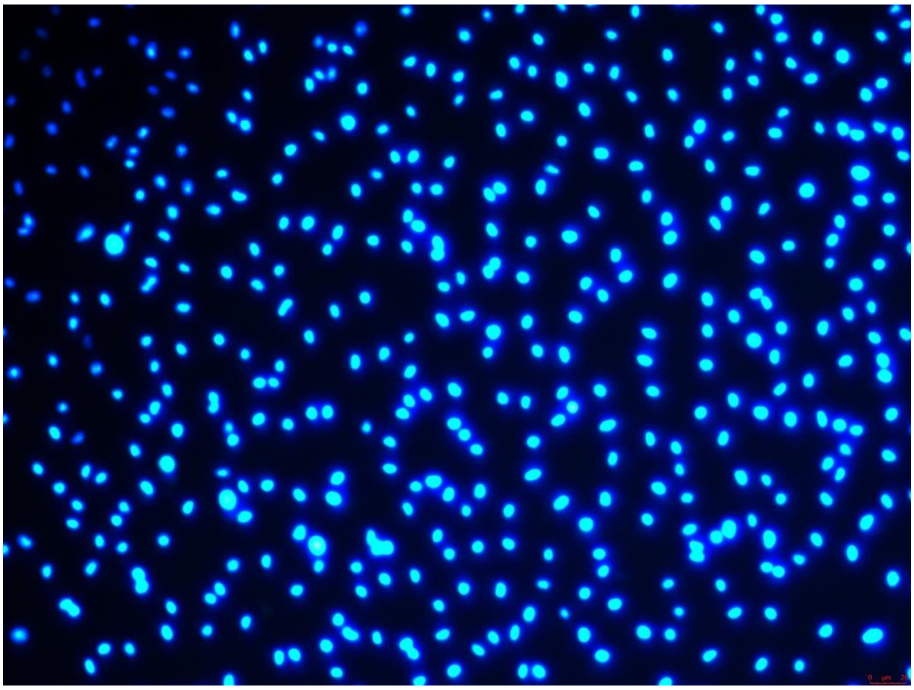
LPS+ATP-DCFH-DA	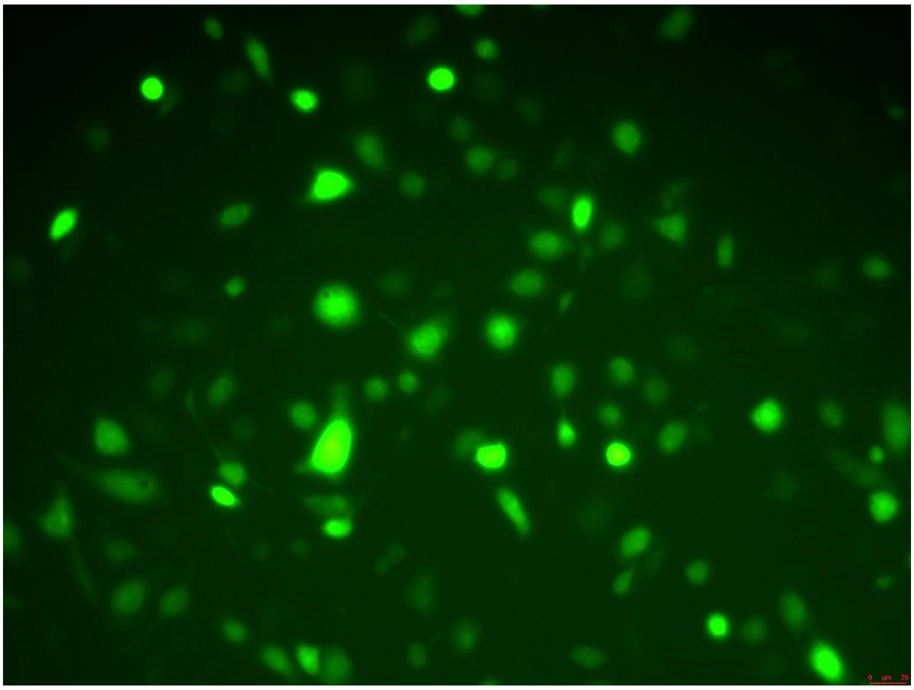

LPS+ATP -Hoechst	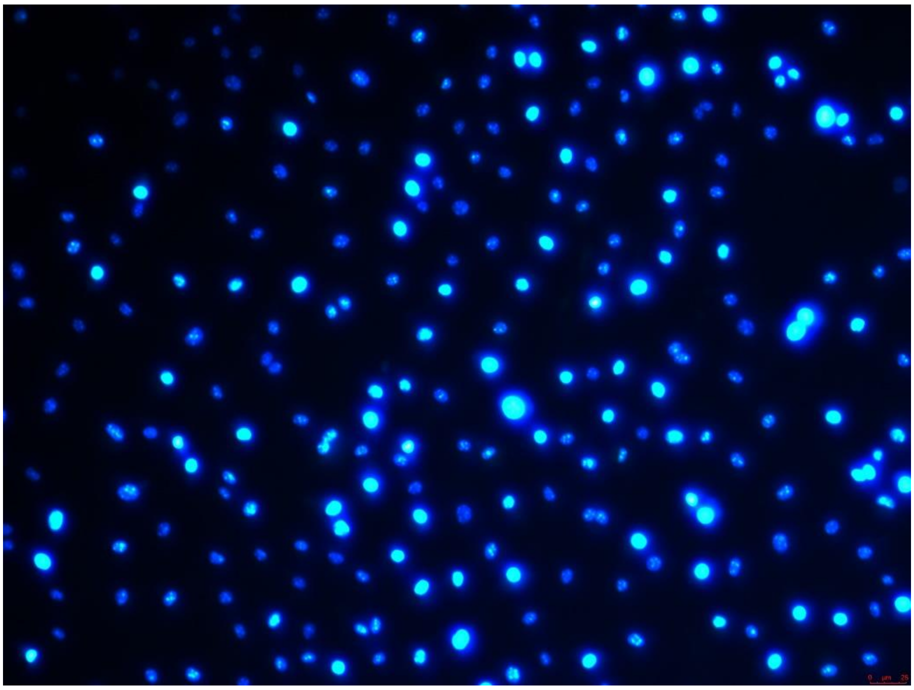
KM 50 μM-DCFH-DA	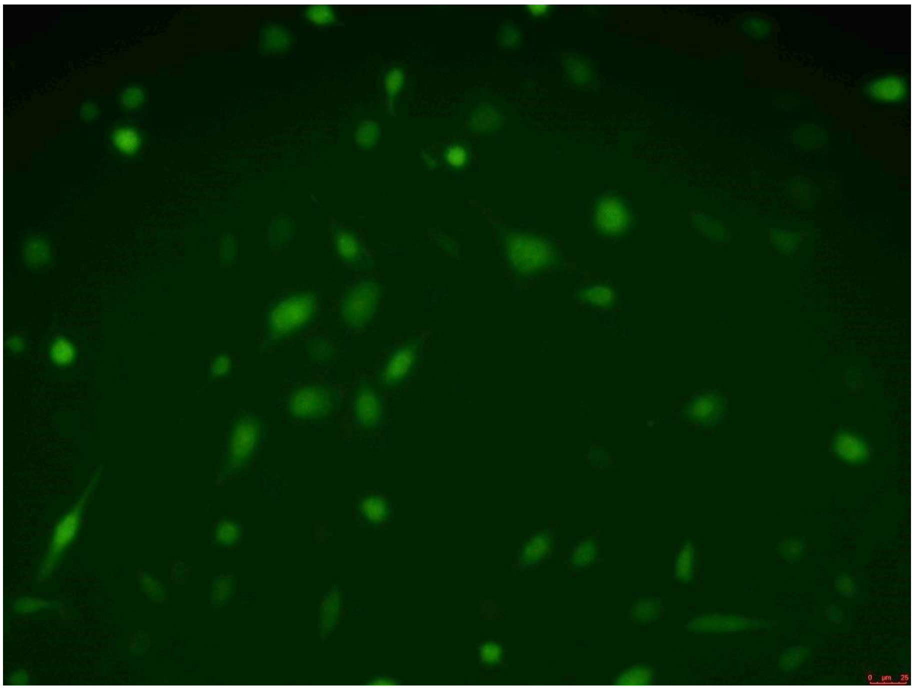

KM 50 μM-Hoechst	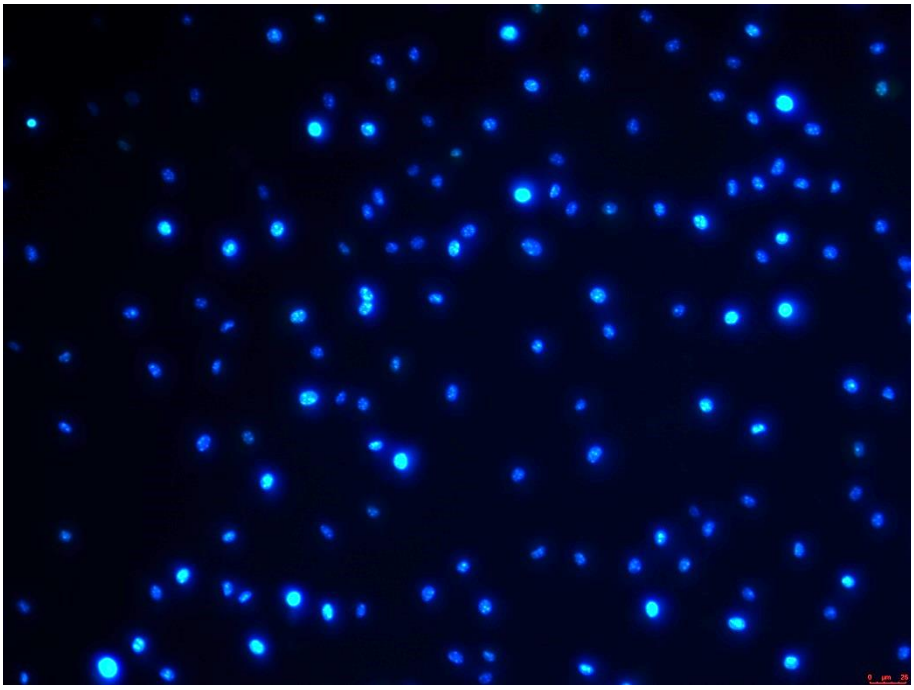
KM 100 μM-DCFH-DA	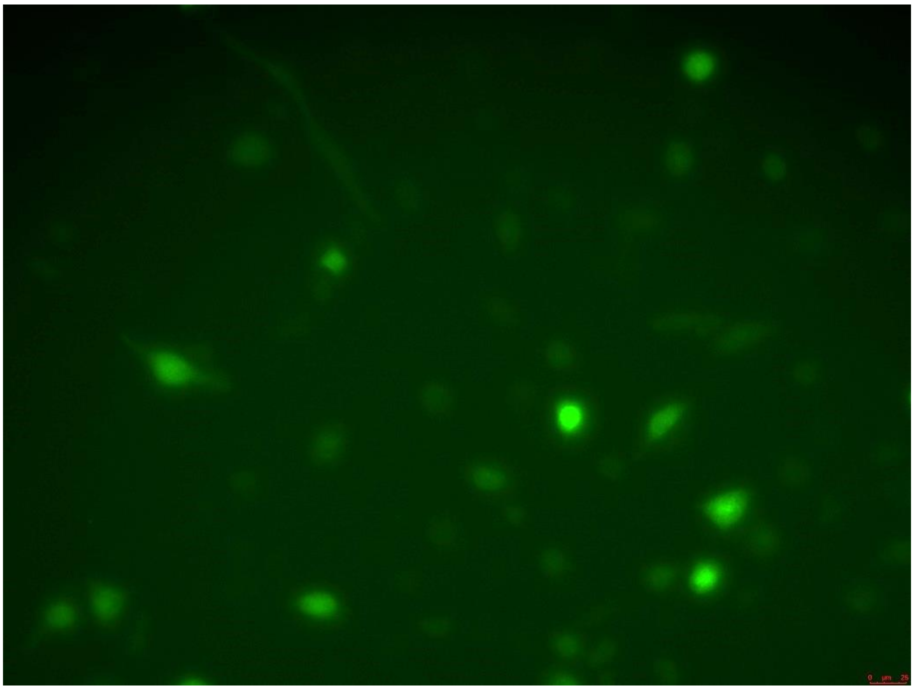

KM 100 µM-Hoechst	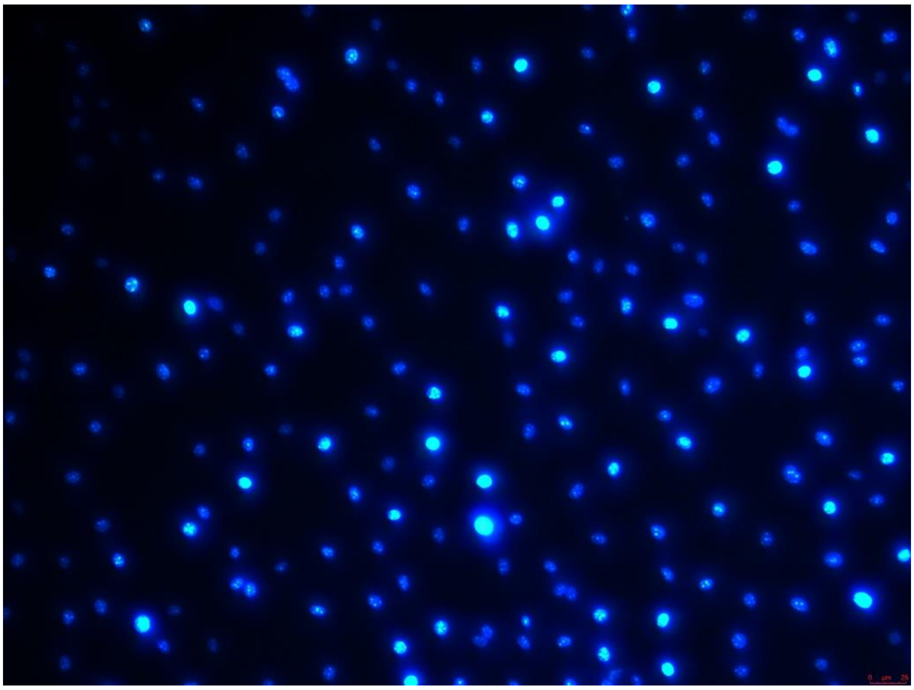
KM 200 µM-DCFH-DA	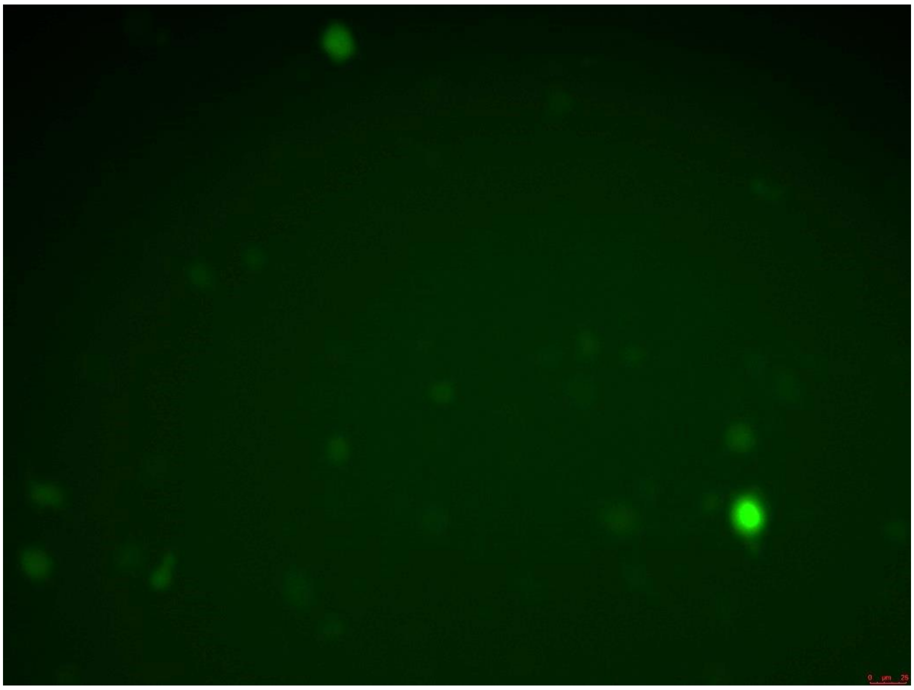

KM 200 μM-Hoechst	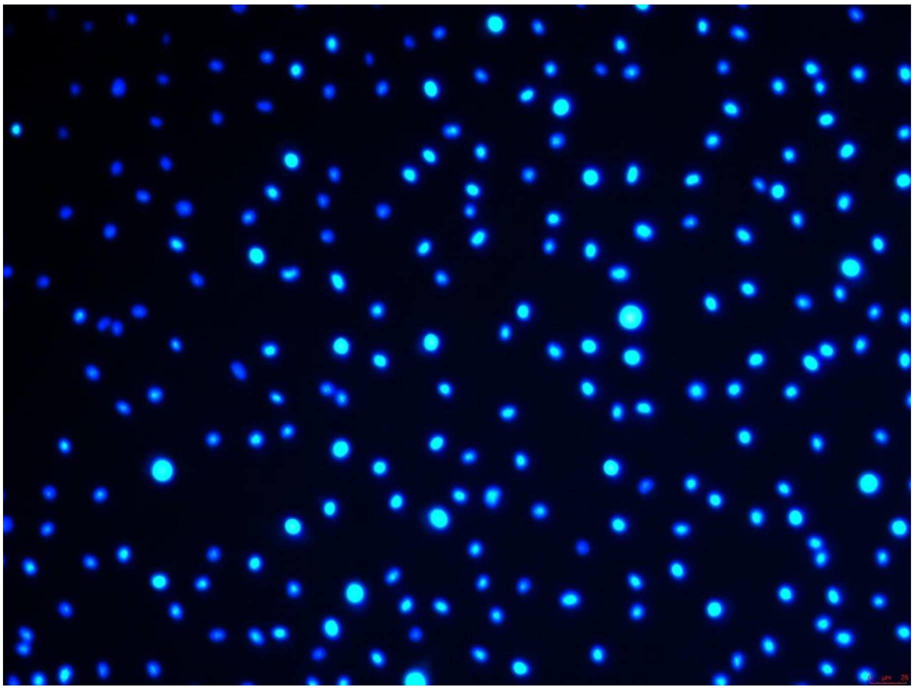

The original western blots of figure 7.

FIGURE 7E

p-AMPK	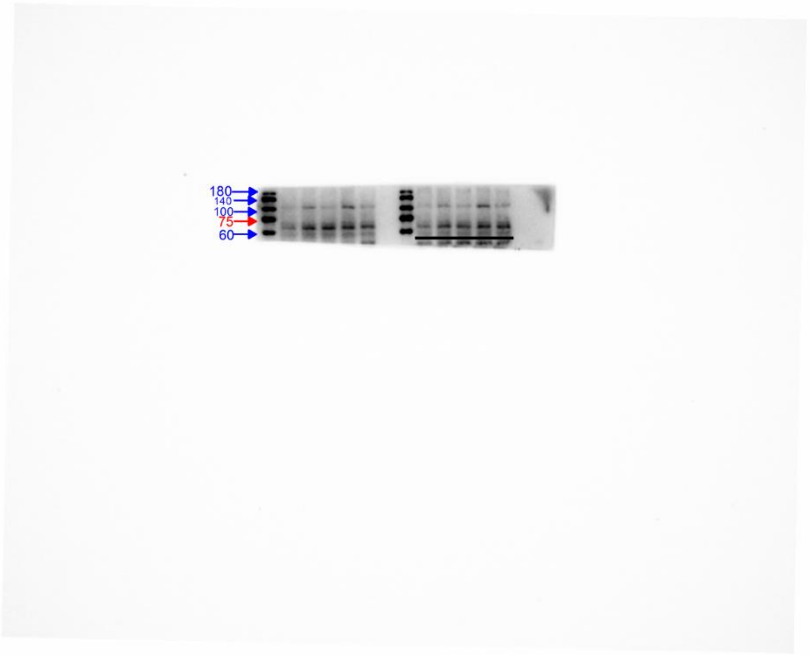

AMPK	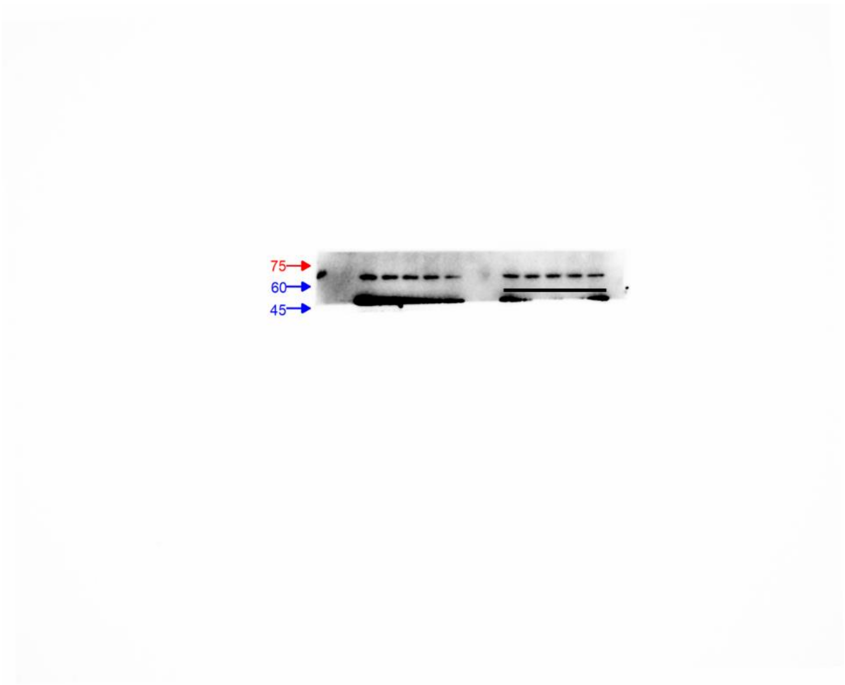
β-actin	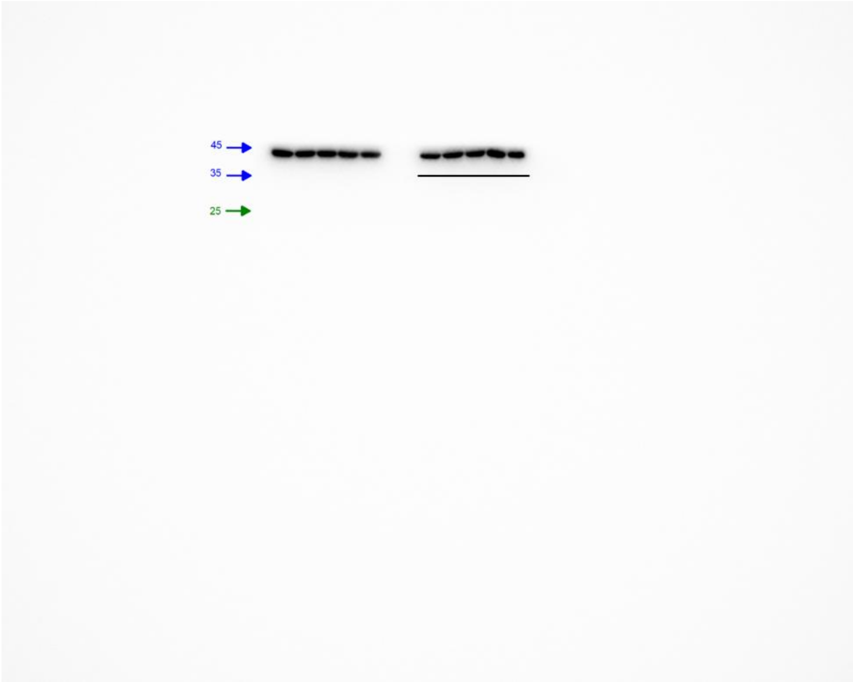

The original figures in supplementary figure 1.

D1	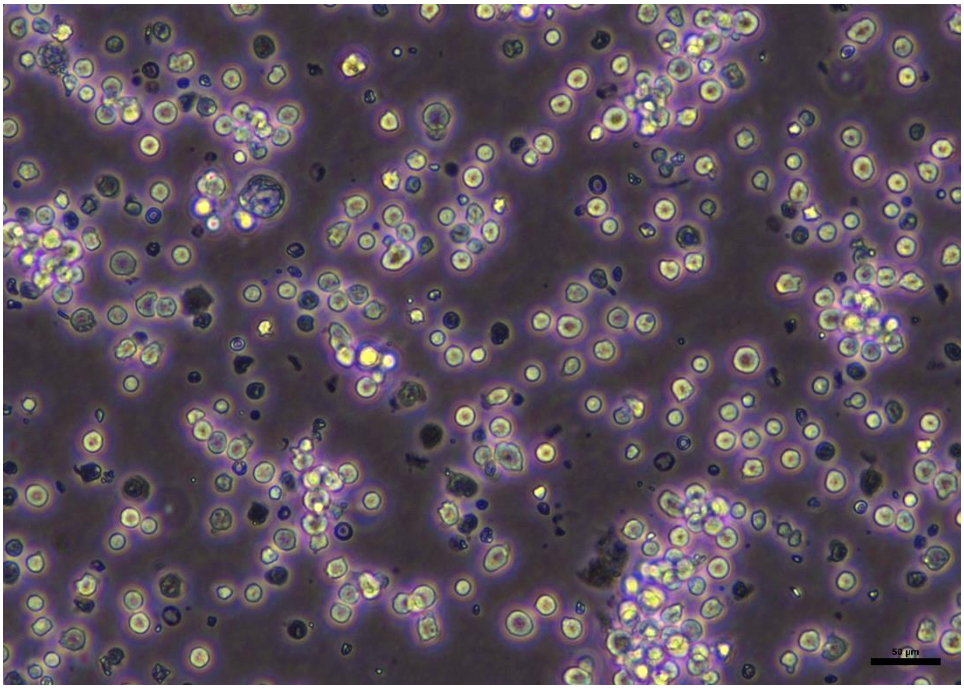
D7	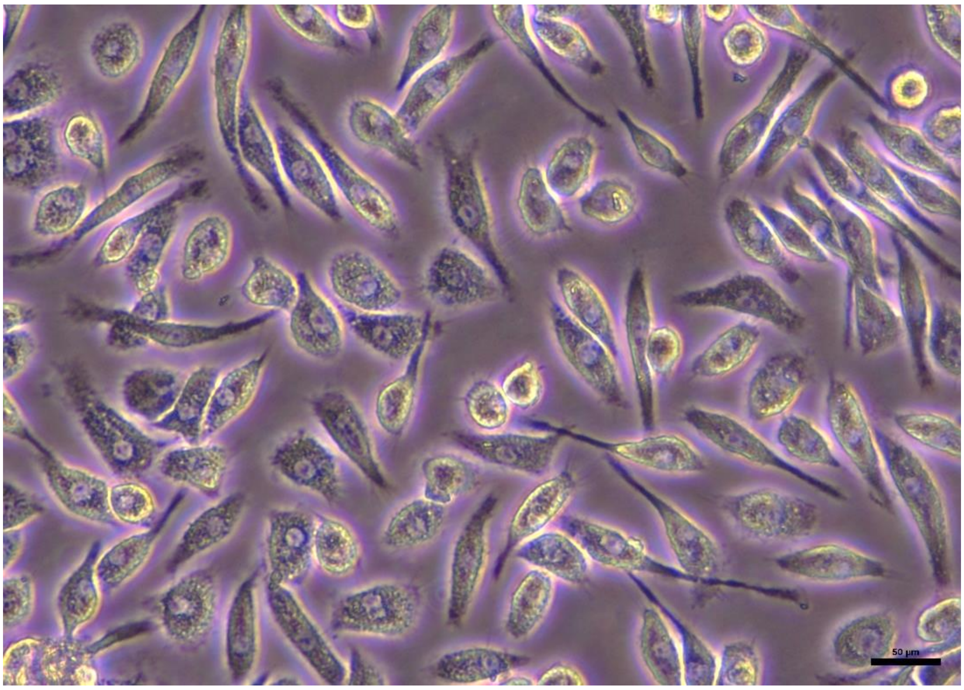

The original western blots of figure S2.

FIGURE S2A BMDM

<table>
<tr><td>NLRP3</td><td>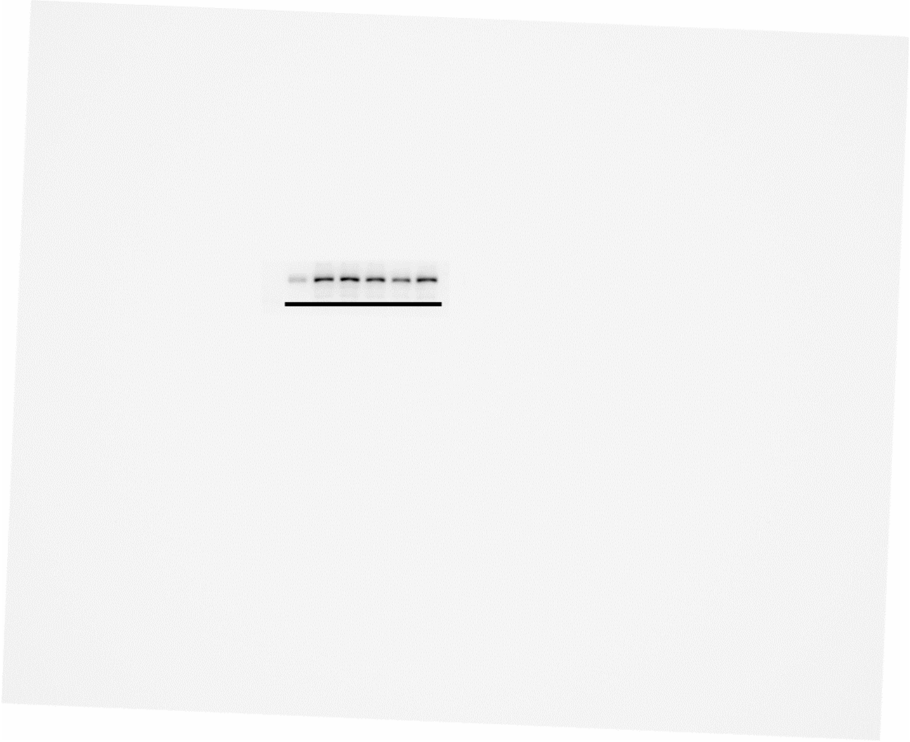</td></tr>
<tr><td>Pro-IL-1β</td><td>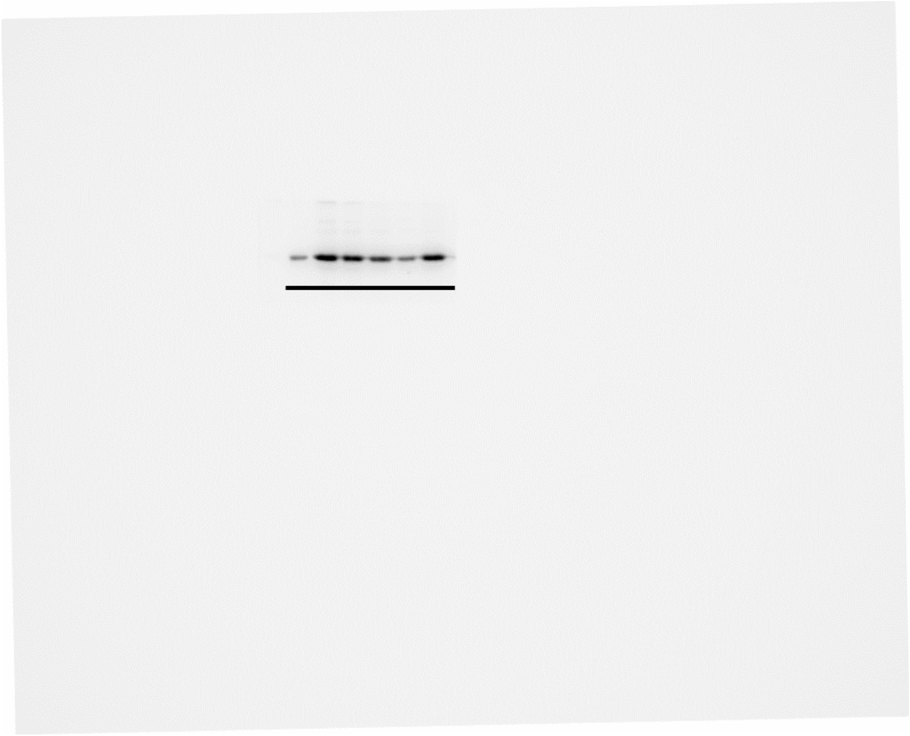</td></tr>
</table>

β-actin	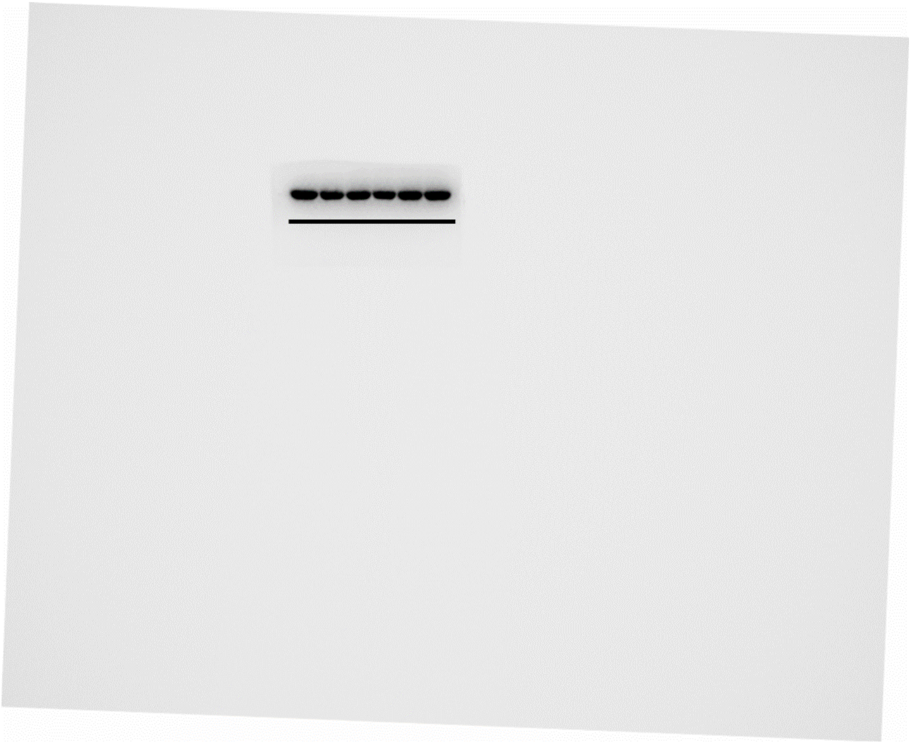

FIGURE S2B PMA-induced THP-1

NLRP3	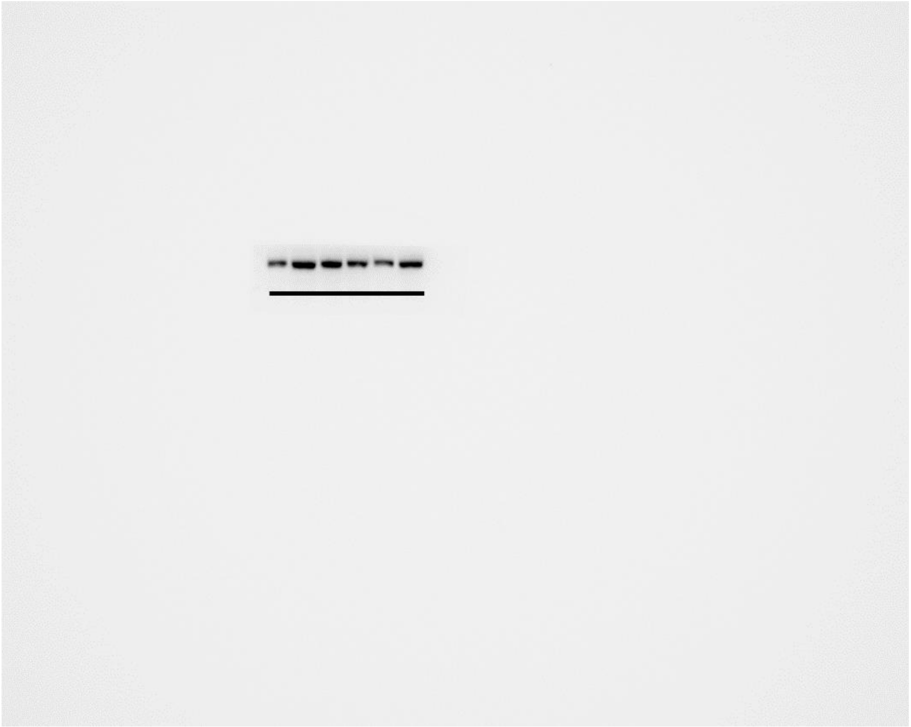

Pro-IL-1β	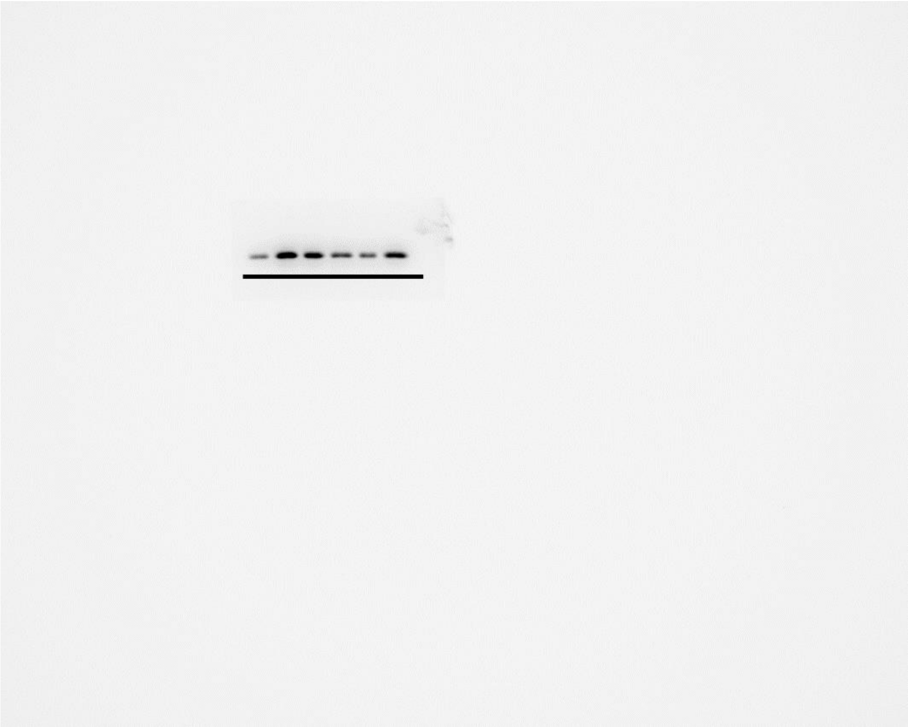
β-actin	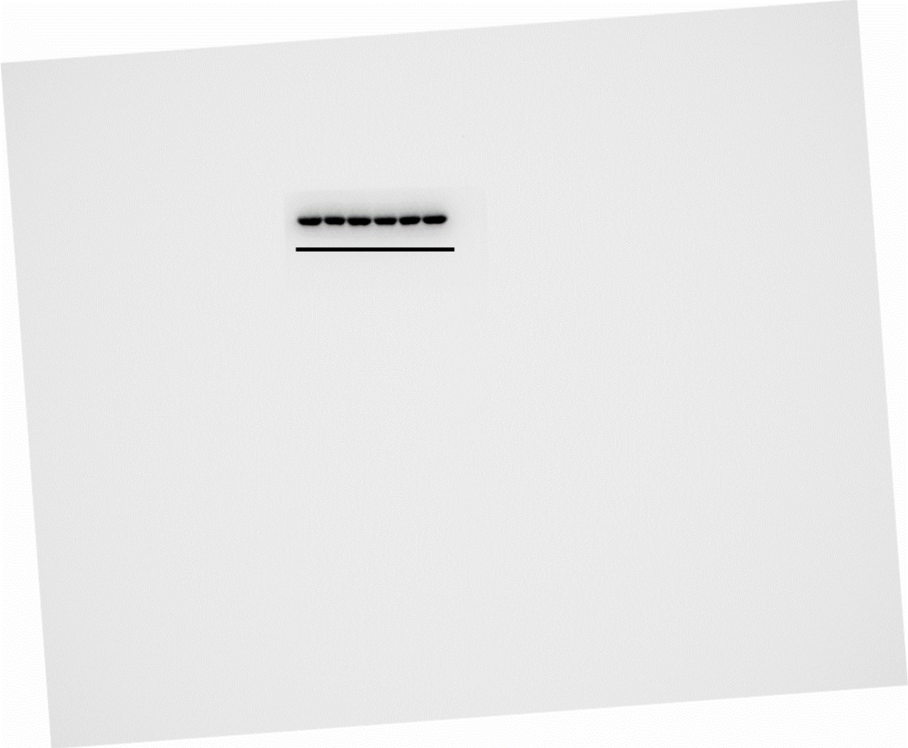

The original figures of immunofluorescent staining in figure S3.

Vehicle<br>p65	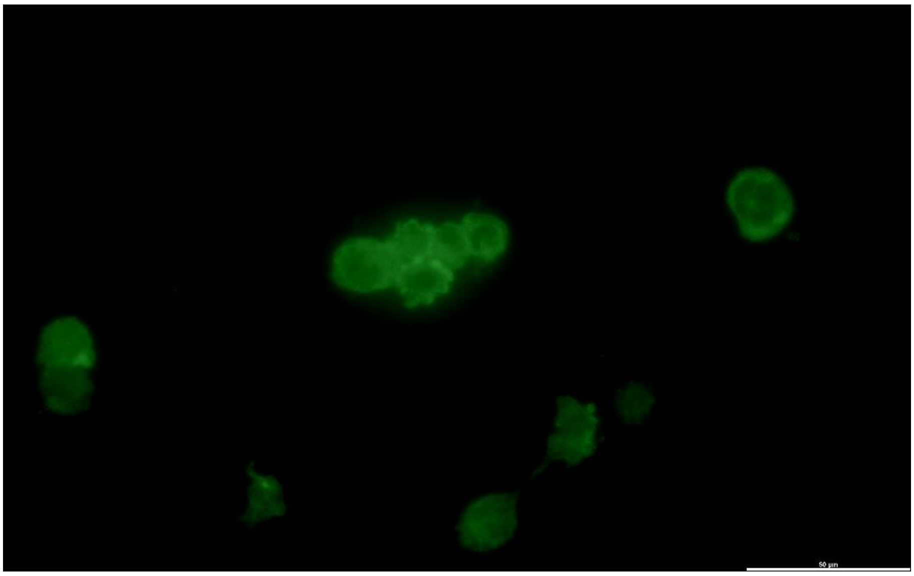
Vehicle<br>Hoechst	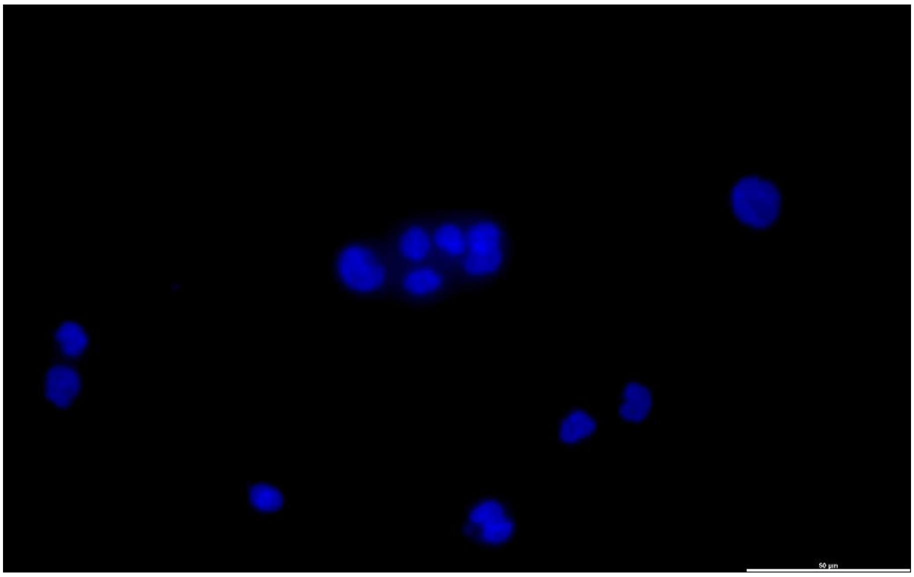
Vehicle<br>Merge	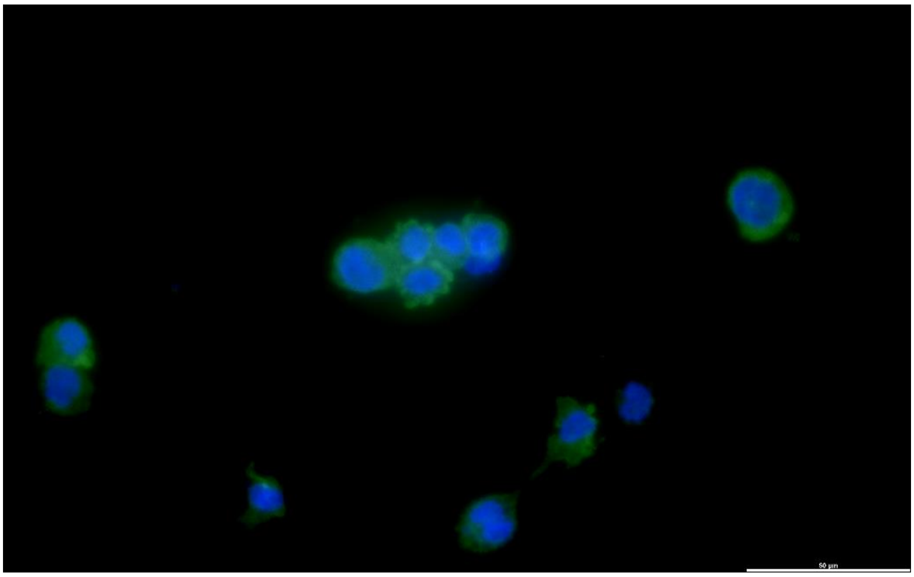

LPS P65	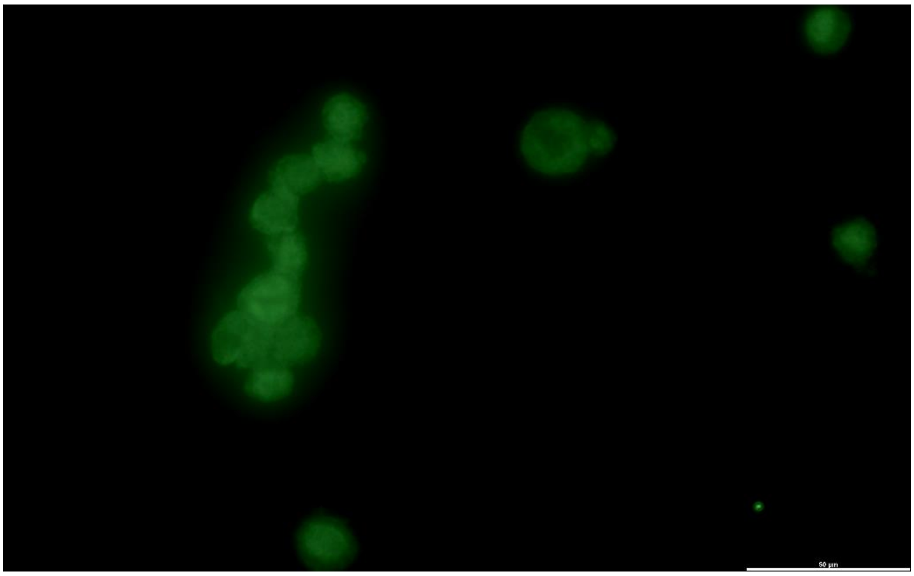
LPS Hoechst	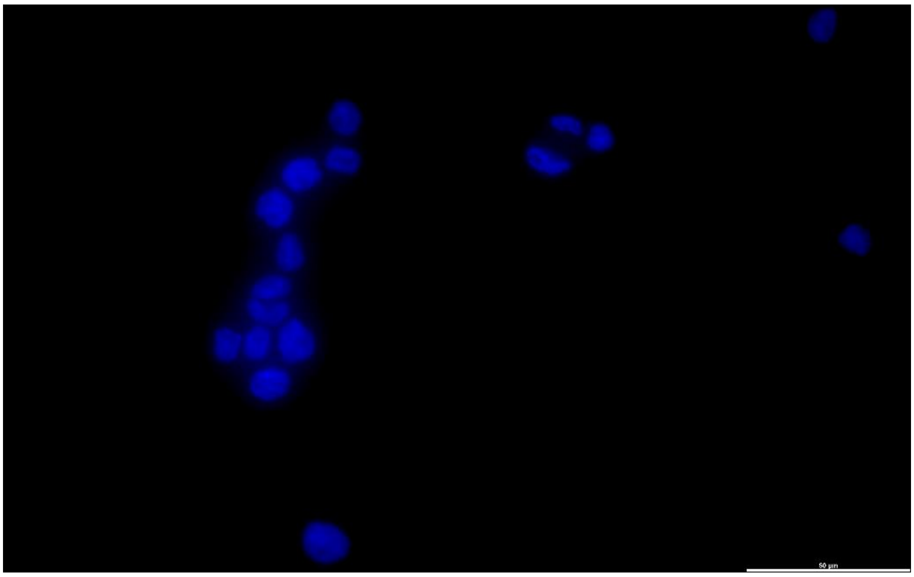
LPS Merge	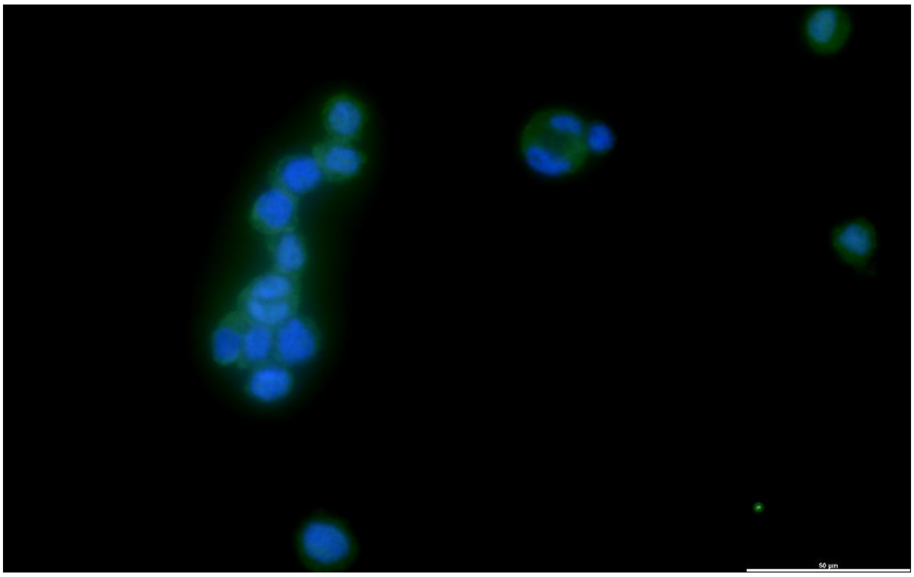

KM+LPS p65	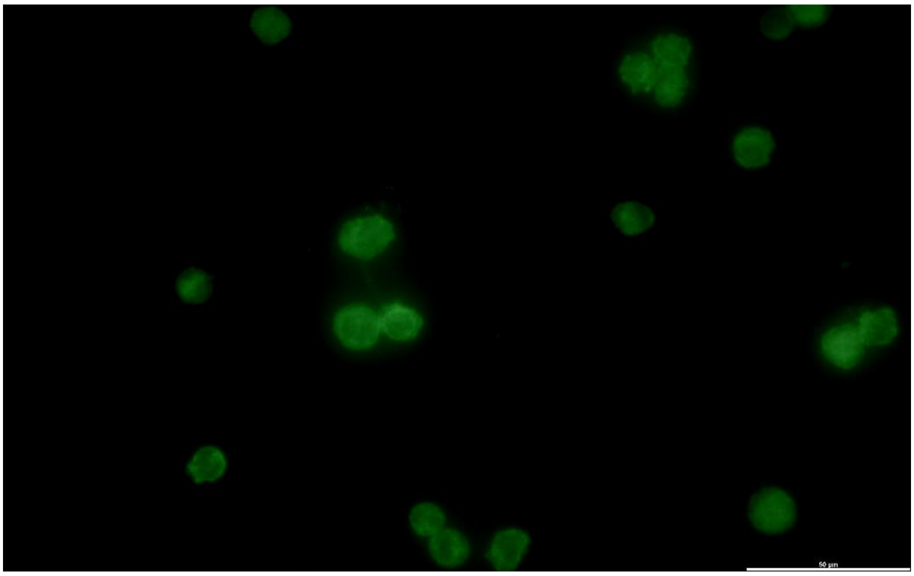
KM+LPS Hoechst	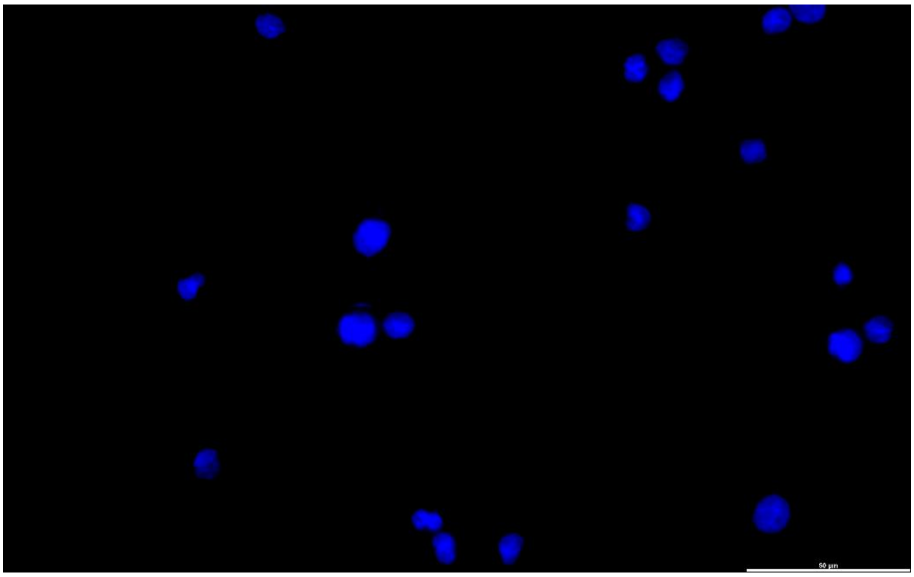
KM+LPS Merge	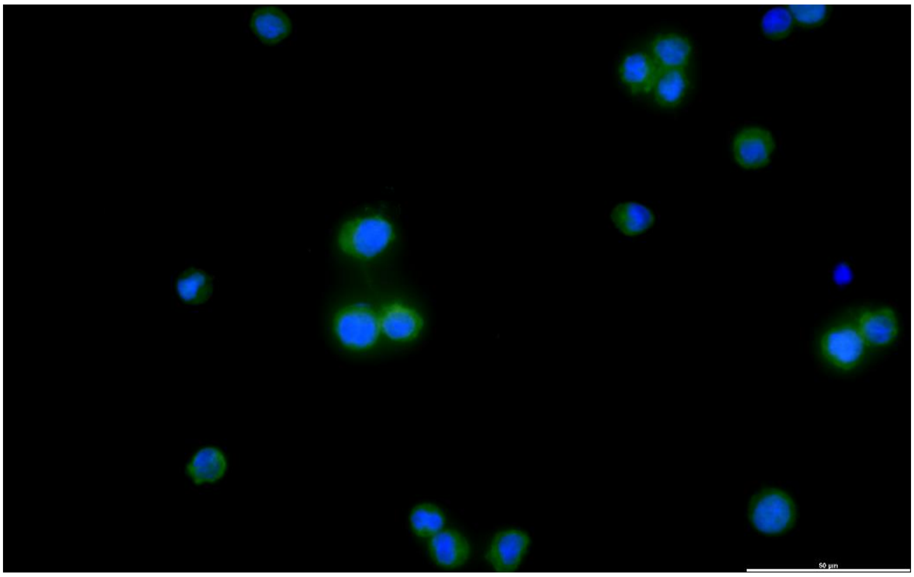

The original western blots of figure S4A.

ASC oligomerization	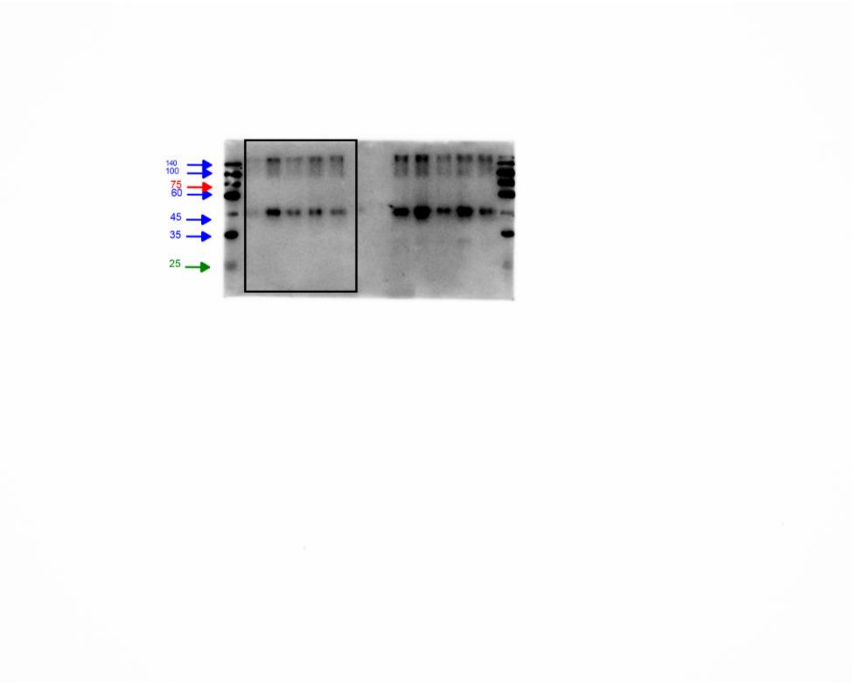
ASC	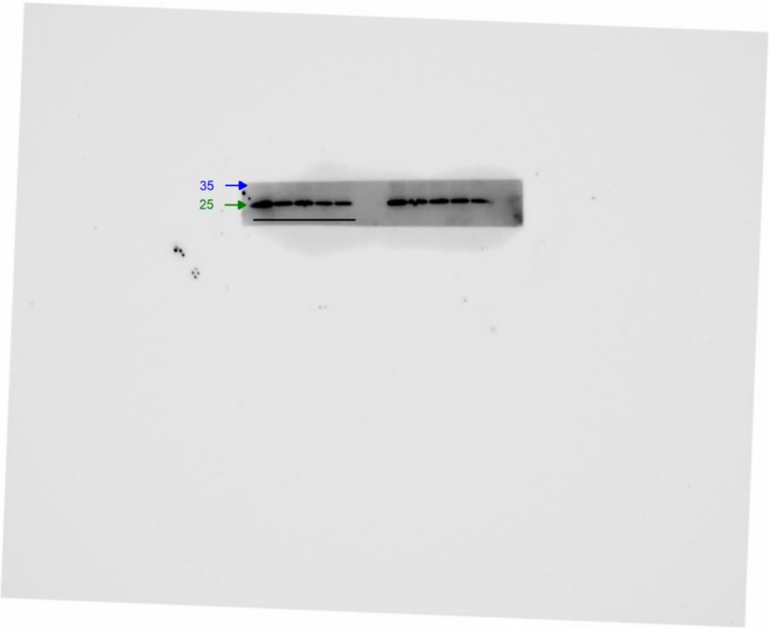

β-actin	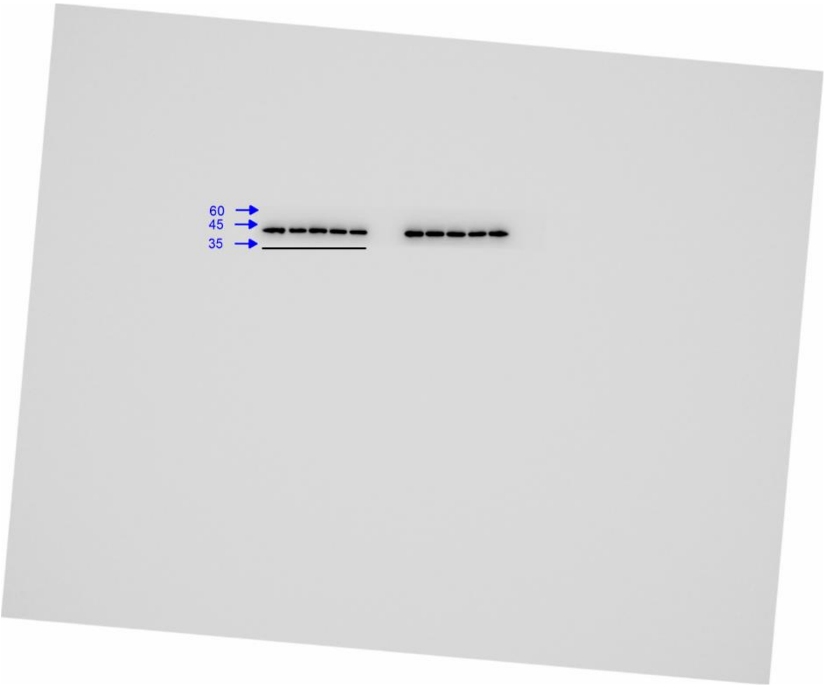

The original figures of immunofluorescent staining in figure S4B.

Vehicle<br>NLRP3	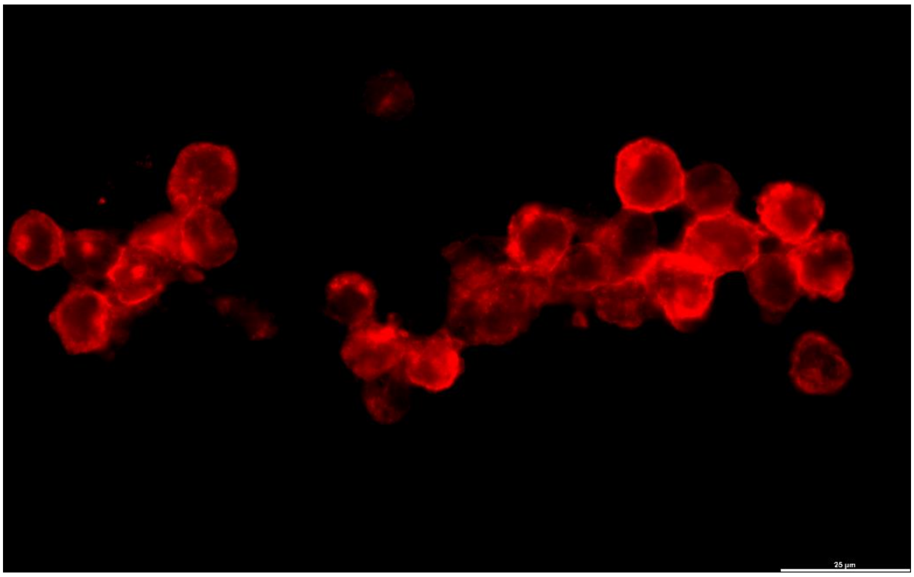

Vehicle<br>ASC	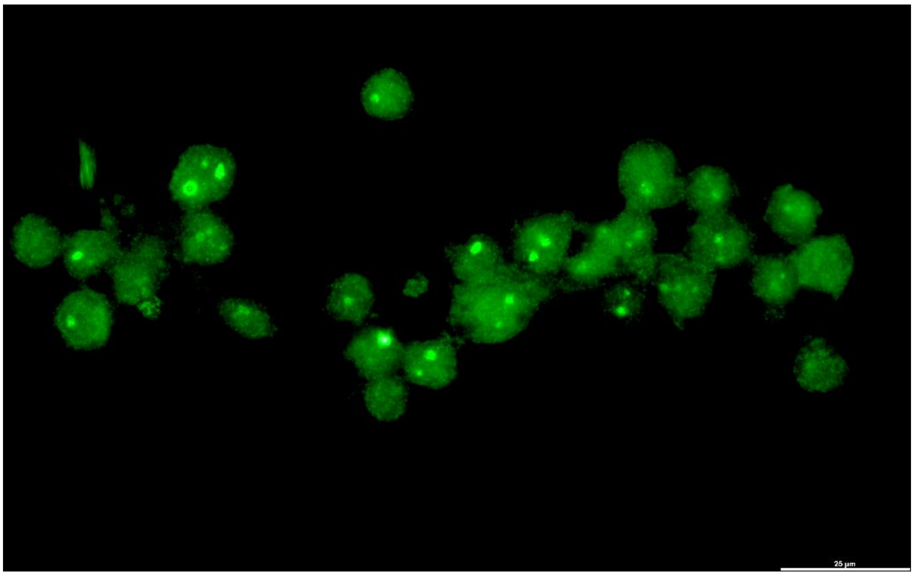
Vehicle<br>Hoechst	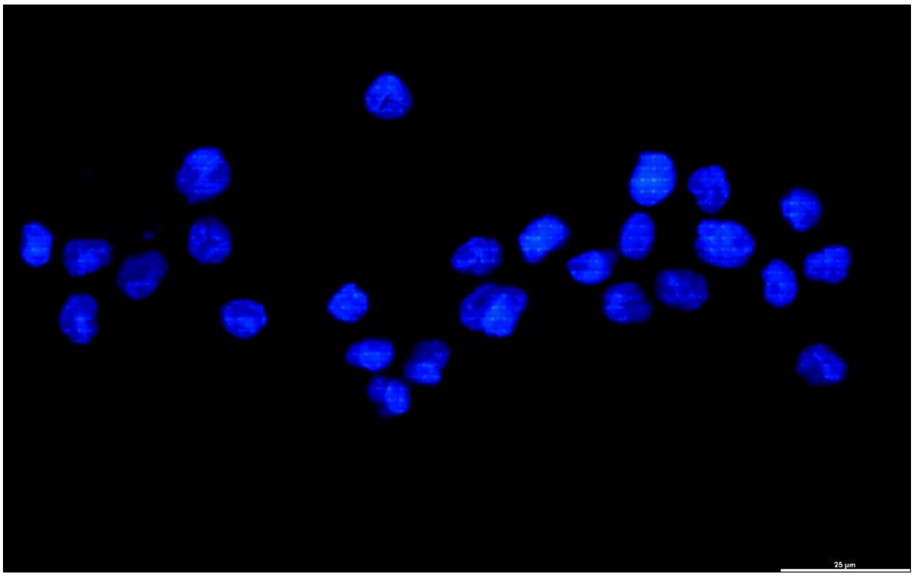
Vehicle<br>Merge	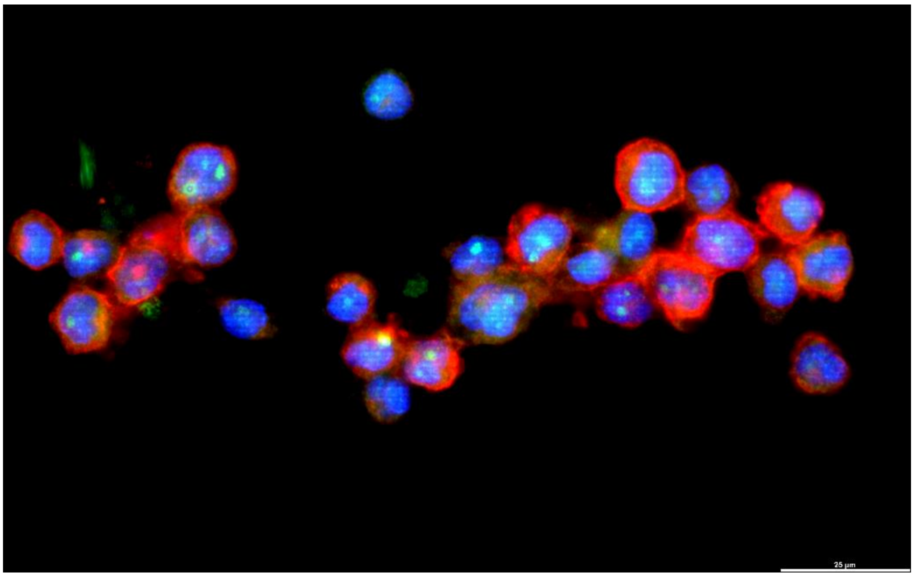

LPS+ATP<br>NLRP3	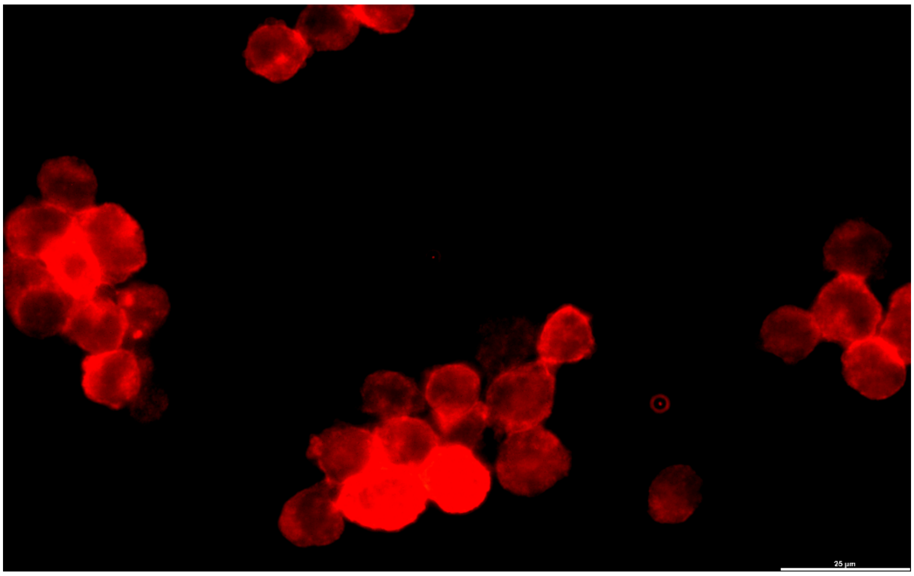
LPS+ATP<br>ASC	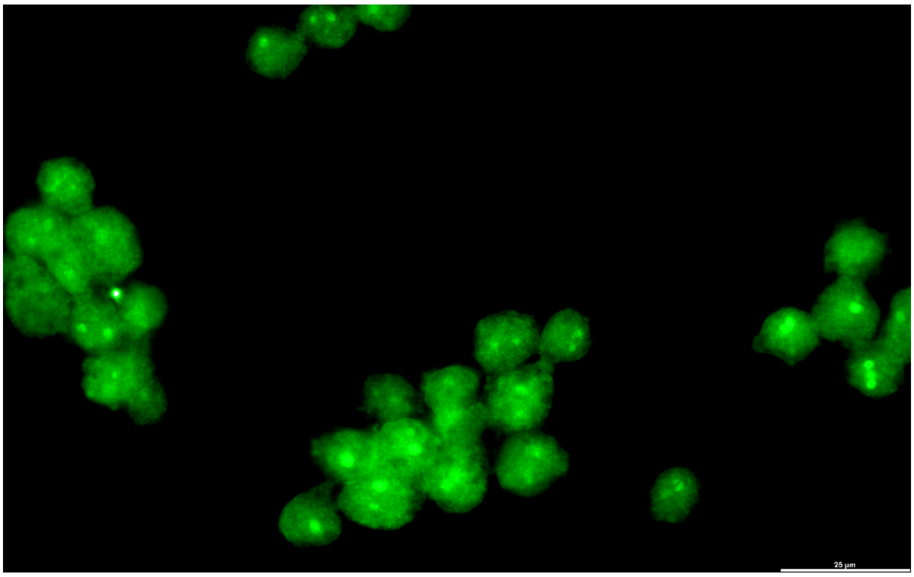
LPS+ATP<br>Hoechst	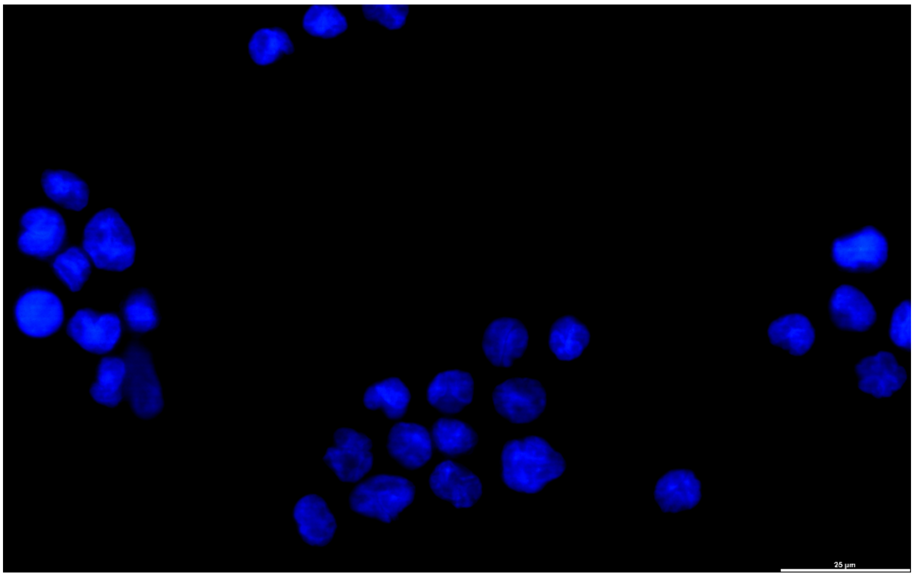

LPS+ATP<br>Merge	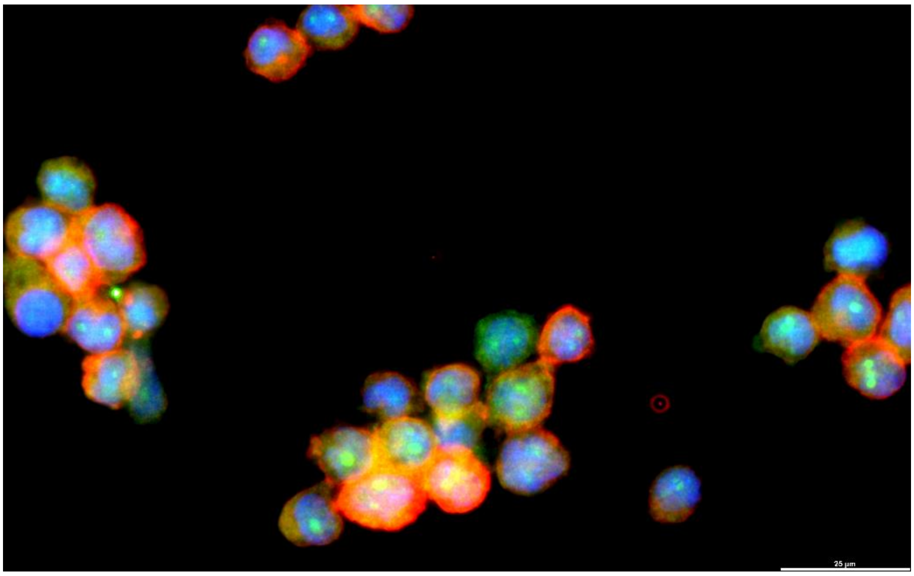
KM+<br>LPS+ATP<br>NLRP3	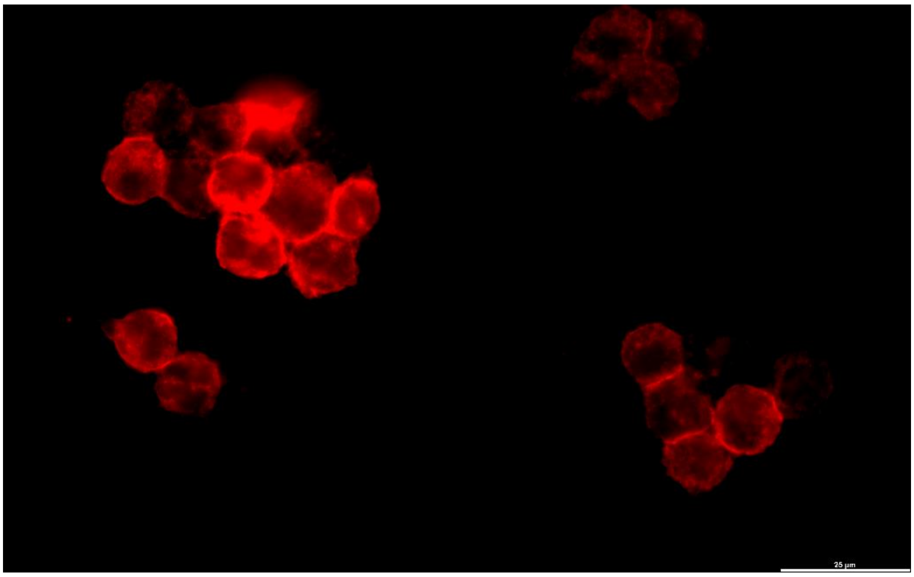
KM+<br>LPS+ATP<br>ASC	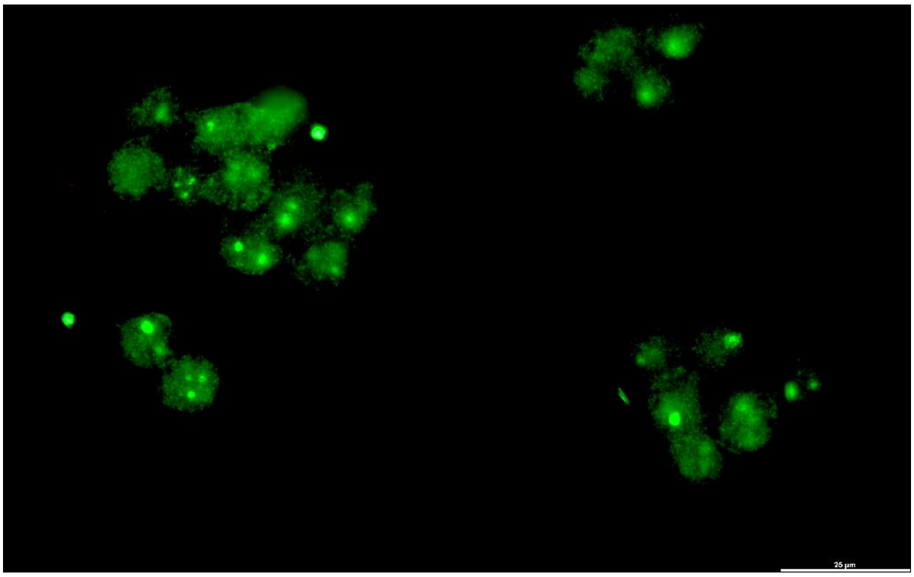

KM+ LPS+ATP Hoechst	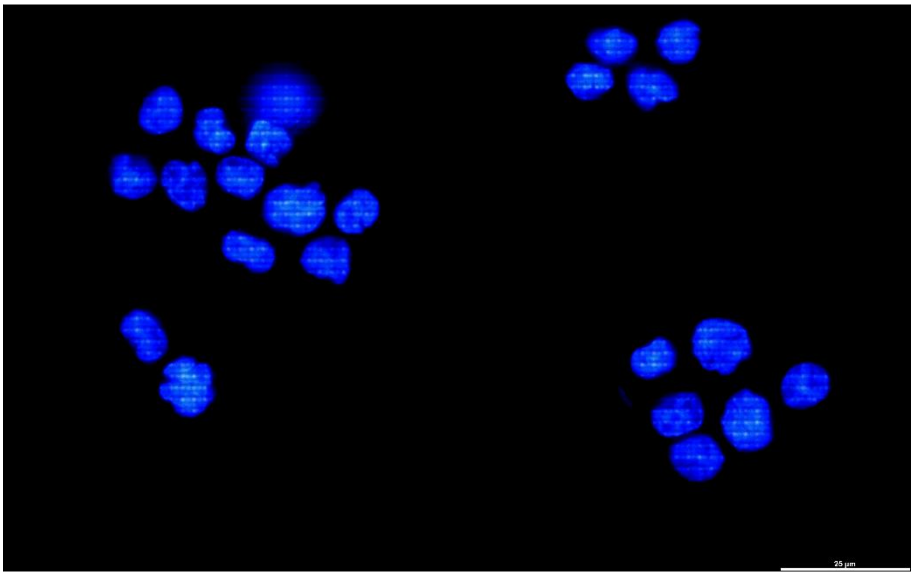
KM+ LPS+ATP Merge	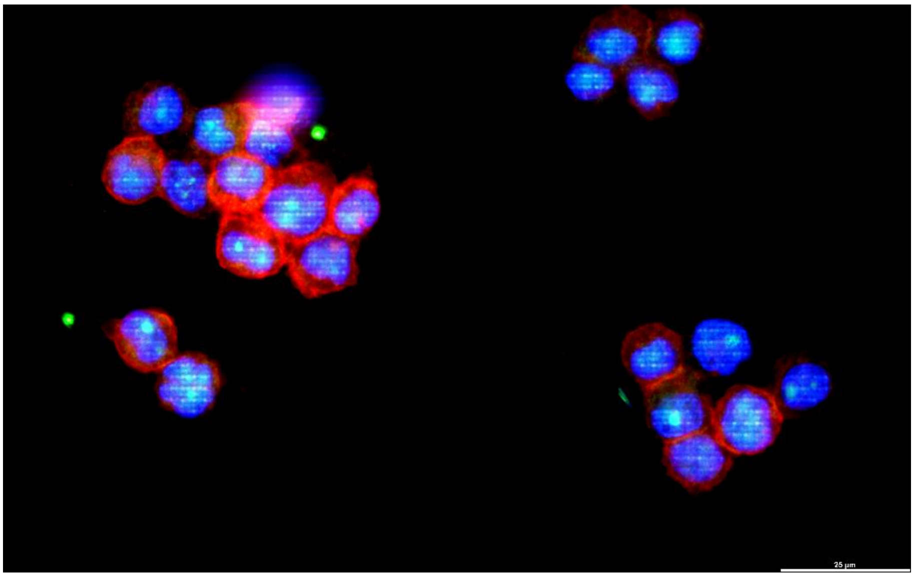

The original figures of DCFH-DA staining in figure S5.

Vehicle-DCFH-DA	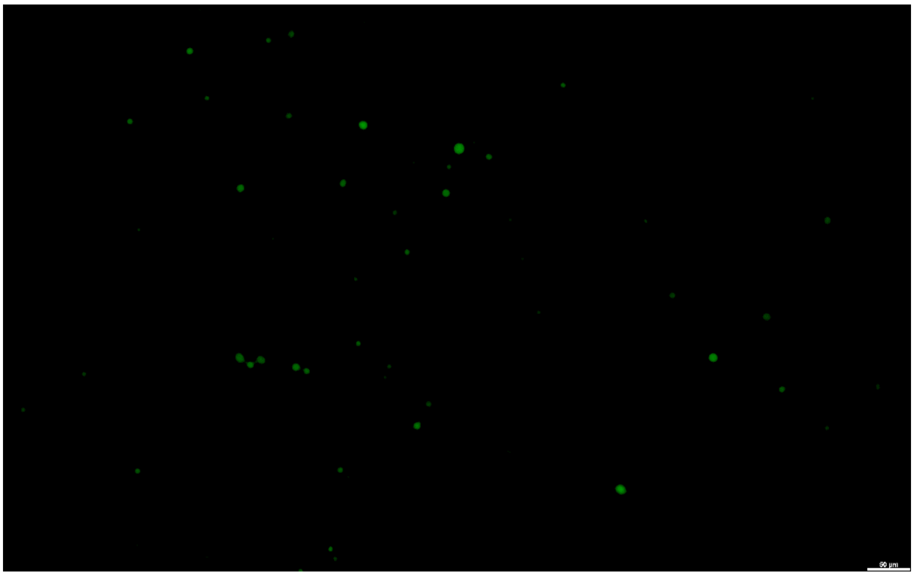
Vehicel-Hoechst	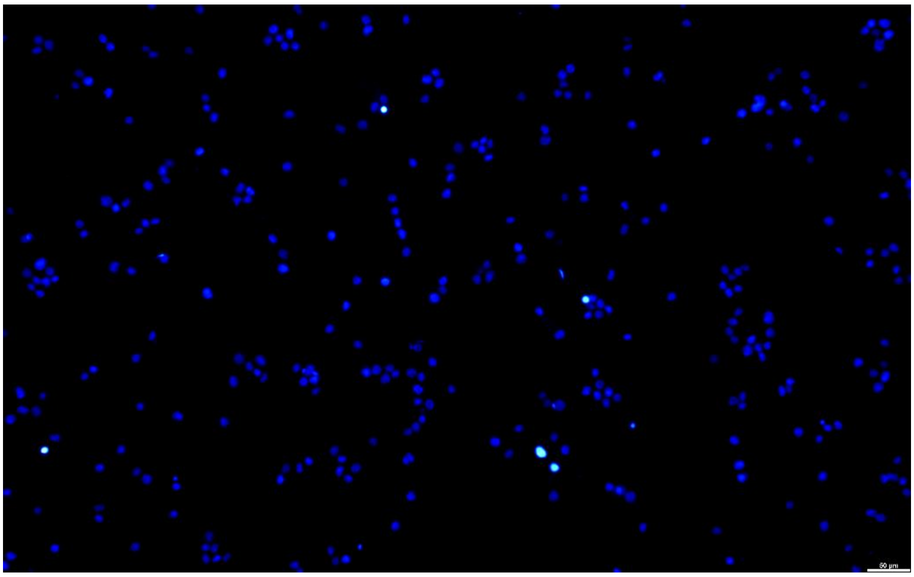
LPS+ATP-DCFH-DA	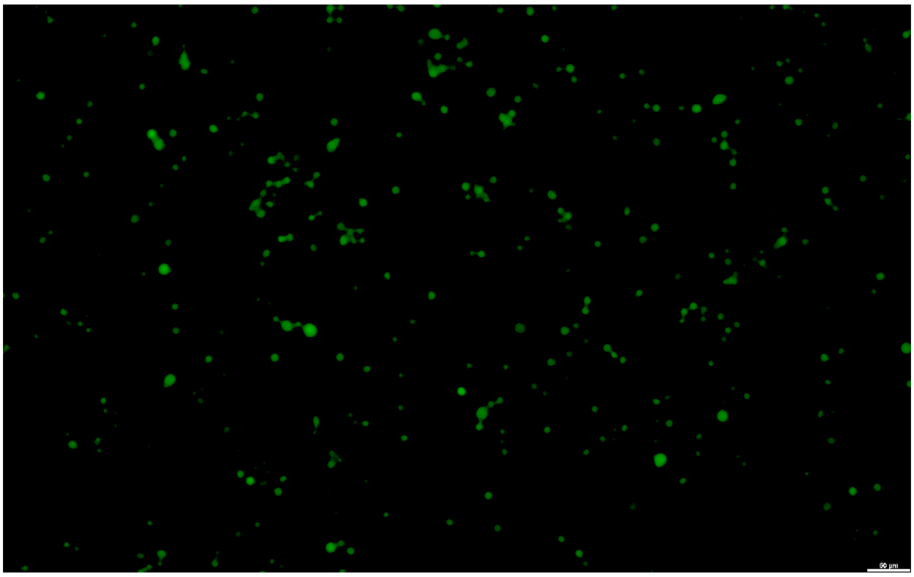

LPS+ATP -Hoechst	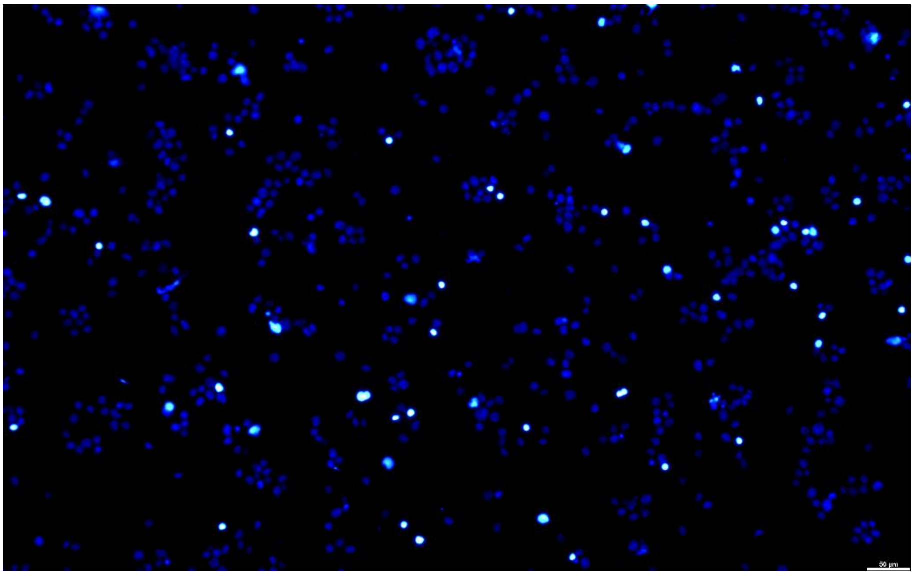
KM 200 μM-DCFH-DA	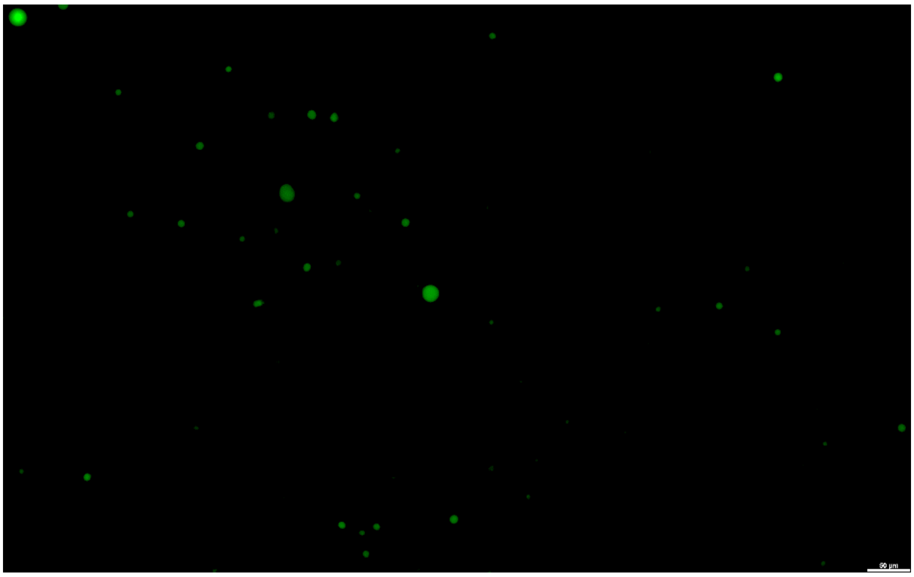
KM 200 μM-Hoechst	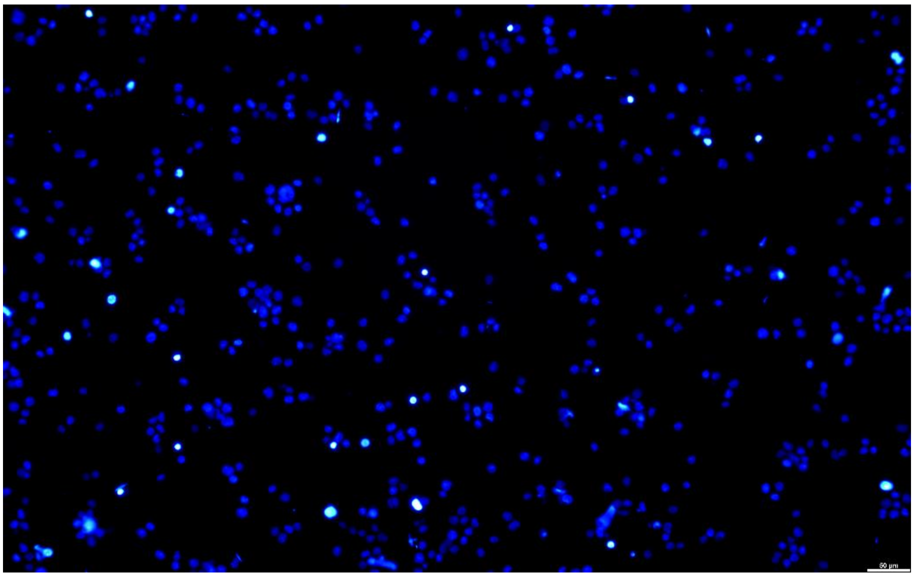
